# GIPR agonism and antagonism decrease body weight and food intake via different mechanisms in male mice

**DOI:** 10.1038/s42255-025-01294-x

**Published:** 2025-04-29

**Authors:** Robert M. Gutgesell, Ahmed Khalil, Arkadiusz Liskiewicz, Gandhari Maity-Kumar, Aaron Novikoff, Gerald Grandl, Daniela Liskiewicz, Callum Coupland, Ezgi Karaoglu, Seun Akindehin, Russell Castelino, Fabiola Curion, Xue Liu, Cristina Garcia-Caceres, Alberto Cebrian-Serrano, Jonathan D. Douros, Patrick J. Knerr, Brian Finan, Richard D. DiMarchi, Kyle W. Sloop, Ricardo J. Samms, Fabian J. Theis, Matthias H. Tschöp, Timo D. Müller

**Affiliations:** 1 Institute for Diabetes and Obesity, Helmholtz, Munich, Germany; 2 German Center for Diabetes Research, DZD, Neuherberg, Germany; 3 Institute of Computational Biology, Helmholtz Munich, Munich, Germany; 4 Department of Physiology, Faculty of Medical Sciences in Katowice, Medical University of Silesia, Katowice, Poland; 5 Department of Pharmacology, Experimental Therapy and Toxicology, Institute of Experimental and Clinical Pharmacology and Pharmacogenomics, Eberhard Karls University, Tübingen, Germany; 6 Department of Computational Health, Institute of Computational Biology, Helmholtz, Munich, Germany; 7 Department of Mathematics, School of Computation, Information and Technology, Technical University of Munich, Munich, Germany; 8 Medizinische Klinik und Poliklinik IV, Klinikum der Universität, Ludwig–Maximilians Universität München, Munich, Germany; 9 Indiana Biosciences Research Institute, Indianapolis, IN USA; 10 Diabetes, Obesity and Complications Therapeutic Area, Eli Lilly and Company, Indianapolis, IN USA; 11 Department of Chemistry, Indiana University Bloomington, Bloomington, IN USA; 12 TUM School of Life Sciences Weihenstephan, Technical University of Munich, Munich, Germany; 13 Helmholtz Munich, Munich, Germany; 14 Division of Metabolic Diseases, Department of Medicine, Technische Universität, Munich, Germany; 15 Walther-Straub Institute for Pharmacology and Toxicology, Ludwig–Maximilians University Munich, Munich, Germany

**Keywords:** Obesity, Type 2 diabetes

## Abstract

Agonists and antagonists of the glucose-dependent insulinotropic polypeptide receptor (GIPR) enhance body weight loss induced by glucagon-like peptide-1 receptor (GLP-1R) agonism. However, while GIPR agonism decreases body weight and food intake in a GLP-1R-independent manner via GABAergic GIPR^+^ neurons, it remains unclear whether GIPR antagonism affects energy metabolism via a similar mechanism. Here we show that the body weight and food intake effects of GIPR antagonism are eliminated in mice with global loss of either *Gipr* or *Glp-1r* but are preserved in mice with loss of *Gipr* in either GABAergic neurons of the central nervous system or peripherin-expressing neurons of the peripheral nervous system. Single-nucleus RNA-sequencing shows opposing effects of GIPR agonism and antagonism in the dorsal vagal complex, with antagonism, but not agonism, closely resembling GLP-1R signalling. Additionally, GIPR antagonism and GLP-1R agonism both regulate genes implicated in synaptic plasticity. Collectively, we show that GIPR agonism and antagonism decrease body weight via different mechanisms, with GIPR antagonism, unlike agonism, depending on functional GLP-1R signalling.

## Abstract (web summary)

This study, together with a companion manuscript, show that, in mice, weight loss as a result of GIP receptor antagonism requires, and potentiates, functional GLP-1 receptor signalling in the brain, explaining how both GIP receptor agonists and antagonists trigger weight loss through different mechanisms.

## Main

Co-agonism at the receptors for glucagon-like peptide-1 (GLP-1) and glucose-dependent insulinotropic polypeptide (GIP) has been established as a highly effective strategy to manage obesity^1–4^ and type 2 diabetes^5–10^. Although GIPR agonism has long been stigmatized as potentially enhancing body weight via stimulation of adipocyte lipid deposition^11,12^, long-acting GIPR agonists decrease body weight and food intake in diet-induced obese (DIO) mice^13–15^ and amplify weight loss induced by GLP-1R agonism^13–17^. We and others have shown that long-acting GIPR agonists have a preserved ability to decrease body weight and food intake in *Glp-1r-*deficient mice^15,18^, which is lost in obese mice with *Nestin-Cre*-mediated neuronal loss of *Gipr*^15^. We and others further showed a similar effect in mice with *Vgat-Cre-*mediated deletion of *Gipr* in gamma-aminobutyric acid (GABAergic) neurons^14,19^. Consistent with the demonstration that GIPR agonism decreases body weight and food intake via central GIPR signalling in rodents^14,15^, chemogenetic activation of GIPR neurons in either the hypothalamus^20,21^ or the hindbrain^20^ decreases food intake in mice. Although infusion of long-acting (acyl) GIP into the lateral ventricle decreases body weight and food intake in DIO wildtype (WT) mice, these effects vanish in mice with central nervous system (CNS) loss of *Gipr*^15^.

Superiority of the GIPR:GLP-1R co-agonist MAR709 to yield greater weight loss and further inhibition of food intake relative to GLP-1R agonism is diminished in mice with loss of *Gipr* in either the CNS^15^ or in GABAergic neurons^14^, indicating that GIPR agonism also contributes to weight loss induced by such a co-agonist. Notably, while long-acting GIPR agonists act in the brain in a GLP-1R-independent manner to decrease body weight and food intake via GABAergic GIPR neurons, GIPR antagonism also decreases body weight and food intake in DIO mice and non-human primates, particularly when used in co-therapy with GLP-1R agonism^22–28^. Thus, surprisingly, GIPR agonism and antagonism appear to have similar metabolic end points when it comes to body weight control. AMG133, a bispecific hybrid that comprises two GLP-1R agonists conjugated to a monoclonal anti-GIPR antagonist^25,26^ is currently in phase 2 clinical development for the treatment of obesity and type 2 diabetes. It shows superiority in decreasing body weight relative to targeting of each individual receptor in DIO mice and non-human primates^25^. In a recent phase 1 study, AMG133 induced more than 10% weight loss after 12 weeks of treatment in healthy humans^26^. Together, these findings support the notion that both GIPR agonism and antagonism hold therapeutic value to accelerate GLP-1-induced weight loss.

The mechanisms underlying the reduction of body weight induced by GIPR antagonism, however, are largely unknown, although some studies suggest that GIPR agonism and antagonism may decrease body weight via similar mechanisms^29^. Here, to test this hypothesis, we set out to assess the metabolic effects of two validated GIPR antagonists^22,23^ in mice with whole-body or targeted deletion of *Gipr*. Like GIPR agonism^14^, we find that the body weight and food intake reducing effects of GIPR antagonism are lost in global *Gipr*-deficient mice. However, in contrast to GIPR agonism^14^, we find that GIPR antagonism fully retains its body weight and food intake reducing effects in mice with *Vgat-Cre*-mediated deletion of *Gipr* in GABAergic neurons, as well as in mice with loss of *Gipr* in peripherin-expressing neurons of the peripheral nervous system (PNS). However, and again in contrast to GIPR agonism^14,18^, we find that the body weight and food intake inhibitory effects of GIPR antagonism are absent in global *Glp-1r*-deficient mice, suggesting dependency on GLP-1R-mediated signalling. Consistent with this finding, single-nuclei RNA-sequencing (snRNA-seq) revealed that GIPR agonism and antagonism have opposing effects in the brain, with GIPR antagonism but not agonism mimicking the transcriptional responses of GLP-1R agonism in the dorsal vagal complex of the hindbrain (DVC), and with GIPR antagonism and GLP-1R agonism both modulating DVC gene programmes implicated in synapse formation and neuronal plasticity. Collectively, we show that while GIPR agonism and antagonism have similar effects on body weight and food intake, they do so via different neuronal mechanisms, with GIPR antagonism, but not GIPR agonism, depending on GLP-1R signalling to affect energy metabolism.

## Results

### Metabolic effects of GLP-1R agonism–GIPR antagonism in DIO *Vgat-Gipr* KO mice

**Fig. 1:**
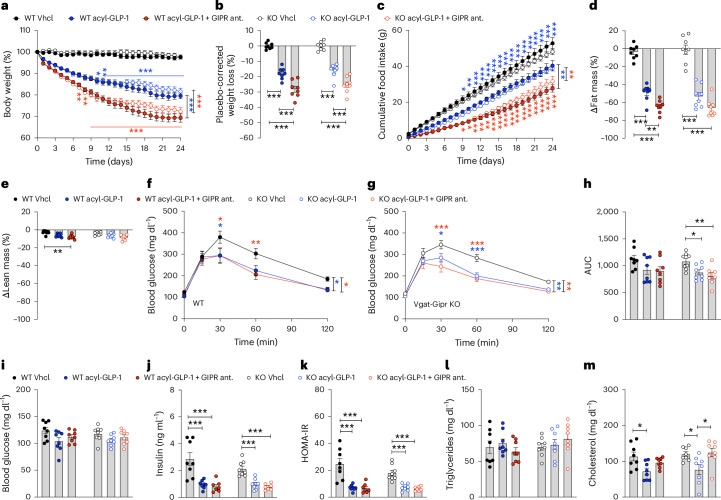
GLP-1R agonism and GIPR antagonism have similar effects on metabolism in HFD-fed *Vgat-Gipr* KO mice. **a**–**c**, Body weight development (**a**), placebo-corrected weight (**b**) and cumulative food intake (**c**) of 33-week-old male C57BL/6J WT or *Vgat-Gipr* KO mice treated daily over 24 days with either vehicle (Vhcl), acyl-GLP-1 (10 nmol kg^−1^) or the combination of acyl-GLP-1 (10 nmol kg^−1^) and a GIPR antagonist (ant.) (1,500 nmol kg^−1^) (*n* = 8 mice each group). **d**–**h**, Body composition (fat mass (**d**) and lean mass (**e**)) and i.p. glucose tolerance (**f** and **g**) with corresponding area under curve (**h**) of 36-week-old male C57BL/6J WT and *Vgat-Gipr* KO mice (*n* = 8 each group) after 24 days of treatment. **i**, Fasting plasma levels of blood glucose (*n* = 8 each group) in 36-week-old male C57BL/6J WT or *Vgat-Gipr* KO mice. **j**,**k**, Fasting plasma levels of insulin (**j**) and corresponding HOMA-IR (**k**) in in 36-week-old male C57BL/6J WT and *Vgat-Gipr* KO mice treated either with vehicle (*n* = 8 WT and *n* = 8 KO), acyl-GLP-1 (*n* = 8 WT and *n* = 8 KO) or the co-therapy of acyl-GLP-1 and the GIPR antagonist (*n* = 7 WT and n = 7 KO). **l**,**m**, Ad libitum plasma levels of triglycerides (**l**) and cholesterol (**m**) in 36-week-old male C57BL/6J WT or *Vgat-Gipr* KO mice (*n* = 8 mice each group). Data in **a**, **c**, **f** and **g** were analysed by repeated measures two-way ANOVA with Bonferroni’s post hoc test for comparison of individual timepoints. Data in **b**, **d**, **e**, **h** and **i**–**m** were analysed using one-way ANOVA. Cumulative food intake (**c**) was assessed per cage in *n* = 8 double-housed mice. Data represent mean ± s.e.m.; *P < 0.05, **P < 0.01 and ***P < 0.001. The blue asterisks in **a** and **c** correspond to the comparison of acyl-GLP-1 versus the co-therapy in WT mice, while red asterisks correspond to acyl-GLP-1 versus the co-therapy in the *Vgat-Gipr* KO mice. Individual *P* values are shown in the Source data, unless *P* < 0.0001. Source data.

**Extended Data Fig. 1:**
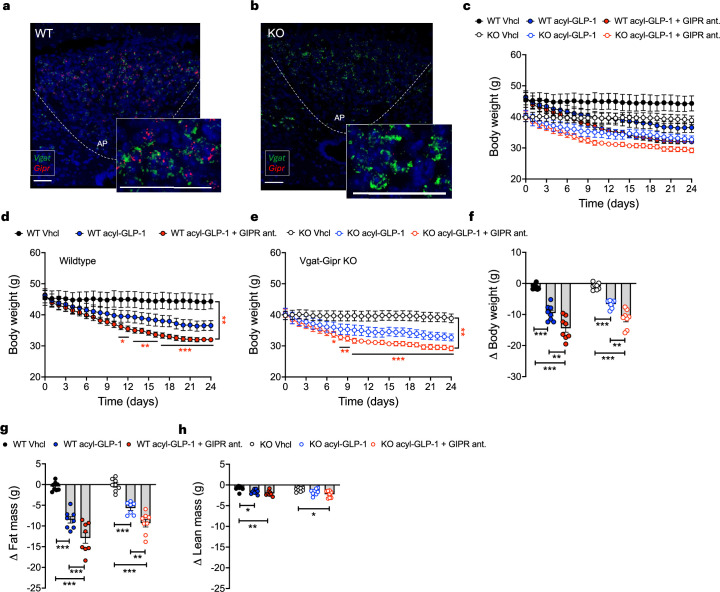
RNAscope validation of GIPR deletion and absolute drug effects corresponding to Fig. 1. RNAscope validation of GIPR deletion in 20 week-old male chow-fed Vgat cre^+^ Gipr^wt/wt^ (WT) and Vgat cre^+^ Gipr^flx/flx^ (KO) mice (pictures are representative examples of n = 3 mice per group) **(a,b)**. Absolute values of body weight development **(c–e)** and total change in body weight **(f)** of 33-wk old male C57BL/6 J wildtype (WT) or *Vgat-Gipr* knockout (KO) mice treated daily over 24 days with either vehicle, acyl-GLP-1 (10 nmol/kg), or the combination of acyl-GLP-1 (10 nmol/kg) and a GIPR antagonist (1,500 nmol/kg) (n = 8 each group). Absolute changes of body composition of 36-wk old male C57BL/6 J wildtype and *Vgat-Gipr* KO mice treated either with Vehicle (n = 8 WT and n = 8 KO), acyl-GLP-1 (n = 8 WT and n = 8 KO), or the co-therapy of acyl-GLP-1 and the GIPR antagonist (n = 8 WT and n = 7 KO) **(g,h)**. Data in panel **d** and **e** were analyzed by repeated measures 2-way ANOVA with Bonferroni’s post-hoc test for comparison of individual time points. Data in panel **f-h** were analyzed using 1-way ANOVA. Data represent mean ± SEM; asterisks indicate * p < 0.05; ** p < 0.01 and *** p < 0.001. Individual p-values are shown in the Data Source file, unless p < 0.0001. Source data

We recently showed that loss of *Gipr* in *Vgat*-expressing GABAergic neurons renders DIO mice resistant to weight loss and inhibition of food intake by GIPR agonism^14^. To test whether *Gipr* antagonism affects energy metabolism via a similar mechanism, we treated DIO *Vgat-Cre*^*+*^*Gipr*^*wt/wt*^ (WT) and *Vgat-Cre*^*+*^*Gipr*^*flx/flx*^ (*Vgat-Gipr* knockout (KO)) mice for 24 days with either vehicle, a long-acting GLP-1R agonist (acyl-GLP-1, 10 nmol kg^−1^)^13–15^ or the combination of acyl-GLP-1 (10 nmol kg^−1^) and a validated long-acting (acylated) peptide GIPR antagonist (1,500 nmol kg^−1^)^22^. *Vgat-Cre*-mediated *Gipr* KO was confirmed by RNAscope (Extended Data Fig. 1a,b). Notably, and in contrast to GIPR:GLP-1R co-agonism, which loses its superiority to GLP-1R agonism with respect to decreases in body weight and food intake in *Vgat-Gipr* KO mice^14^, the co-therapy of GLP-1R agonism and GIPR antagonism maintained the enhanced effect on weight loss (Fig. 1a,b and Extended Data Fig. 1c–f) and on inhibition of food intake (Fig. 1c) relative to treatment with acyl-GLP-1 alone in *Vgat-Gipr* KO mice, without a difference of the co-therapy on either body weight or food intake in WT and *Vgat-Gipr* KO mice. Treatment with the co-therapy decreased body fat and lean tissue mass with comparable efficacy in WT and *Vgat-Gipr* KO mice (Fig. 1d,e and Extended Data Fig. 1g,h). The co-therapy also improved glucose tolerance with comparable efficacy in WT and *Vgat-Gipr* KO mice relative to vehicle controls, albeit without superiority to acyl-GLP-1 (Fig. 1f–h). No differences were observed in fasting levels of blood glucose (Fig. 1i), but levels of insulin (Fig. 1j) and insulin sensitivity, as estimated by homeostatic model assessment for insulin resistance (HOMA-IR) (Fig. 1k), were equally improved by treatment with acyl-GLP-1 and the co-therapy, and without difference between WT and *Vgat-Gipr* KO mice. No differences were observed in plasma levels of triglycerides (Fig. 1l), but levels of cholesterol were decreased after treatment with acyl-GLP-1, but not after treatment with the co-therapy, in both WT and *Vgat-Gipr* KO mice (Fig. 1m). In summary, and in contrast to GIPR agonism^14^, these data indicate that GIPR^+^ GABAergic neurons are dispensable for GIPR antagonism to amplify GLP-1-induced weight loss and inhibition of food intake.

### Metabolic phenotype of mice PNS-deletion of *Gipr*

**Extended Data Fig. 2:**
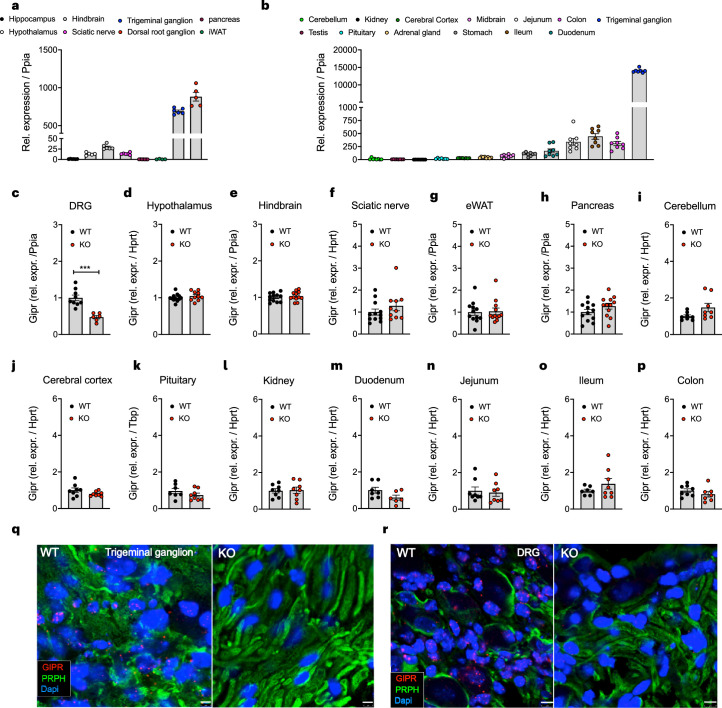
Validation of *Per-Cre*-mediated deletion of *Gipr* in Chow-fed male and female C57BL6/J mice. Expression of *peripherin* corrected by the housekeeping gene *peptidylprolyl isomerase A (Ppia)* in hippocampus (n = 6), hindbrain (n = 6), hypothalamus (n = 5), sciatic nerve (n = 6), trigeminal ganglion (n = 6), dorsal root ganglion (n = 5) and iWAT (n = 4) of 15-week old male chow-fed *Per-Cre*^*+*^*Gipr*^*wt/wt*^ mice, and in pancreas (n = 7) of 45-week old male chow-fed *Per-Cre*^*+*^*Gipr*^*wt/wt*^ mice **(a)**. Expression of *peripherin* corrected by the housekeeping gene Ppia in the cerebellum (n = 8), kidney (n = 8), cerebral cortex (n = 8), midbrain (n = 8), testis (n = 8), pituitary (n = 7), adrenal gland (n = 8), stomach (n = 8), duodenum (n = 8), jejunum (n = 8), ileum (n = 8), colon (n = 8) and trigeminal ganglion (n = 7) of 12-week old male chow-fed WT mice **(b)**. Expression of *Gipr* in dorsal root ganglion (n = 9 WT, n = 6 KO) **(c)**, hypothalamus (n = 12 WT, n = 10 KO) **(d)**, hindbrain (n = 12 each group) **(e)**, sciatic nerve (n = 12 WT, n = 10 KO) **(f)**, eWAT (n = 12 each group) **(g)** and pancreas (n = 12 each group) **(h)** of 45-week old male mice Chow-fed *Per-Cre*^*+*^*Gipr*^*wt/wt*^ (WT) and *Per-Cre*^*+*^*Gipr*^*flx/flx*^ (KO) mice. Expression of *Gipr* in the cerebellum (n = 8 each group) **(i)**, cerebral cortex (n = 8 each group) **(j)**, pituitary (n = 7 WT, n = 8 KO) **(k)**, kidney (n = 8 each group) **(l)**, duodenum (n = 7 WT, n = 6 KO) **(m)**, jejunum (n = 8 each group) **(n)**, ileum (n = 7 WT, n = 8 KO) **(o)** and colon (n = 8 each group) **(p)** of 12-week old male mice Chow-fed WT and KO mice. RNAscope validation of *Gipr* deletion in trigeminal ganglion of 44-week old male chow-fed WT and KO mice **(q)** and of the DRG of 51-week old male chow-fed WT and KO mice **(r)**. Data in panels **q** and **r** are representative examples of n = 3 mice each group. Data in panel **c-f** and **h-p** were analyzed using two-sided, two-tailed Student’s ttest, data in panel **g** were analyzed two-sided by Mann-Whitney test. Data represent mean ± SEM; asterisks indicate * p < 0.05; ** p < 0.01 and *** p < 0.001. Individual p-values are shown in the Data Source file, unless p < 0.0001. Scale bars in panel **q** and **r** are 5 μm. Source data

Expression of *Gipr* has been demonstrated in various regions of the PNS^30,31^. In light of its role in the bi-directional transfer of information between the periphery and the brain, the PNS is well positioned to control energy metabolism, not only by modulating glycaemia via regulation of sympathetic outflow to the skeletal muscle^32^, but also by promoting GIP-induced vasodilation in the mesenteric vasculature, including the adipose tissue^33,34^. Considering these effects, we next assessed whether targeted *Cre*-mediated deletion of *Gipr* in neurons of the PNS affects energy and glucose metabolism. Mice with deletion of *Gipr* in neurons of the PNS were generated by crossing C57BL/6J *Gipr*^*flx/flx*^ mice^35,36^ with C57BL/6J mice that express *Cre*-recombinase under control of the promoter for peripherin (MGI 3841120)^37^. Peripherin is a neuronal intermediate filament protein with largely restricted expression in neurons of the PNS^38^. Consistent with this, we found expression of peripherin largely absent in the hippocampus, DVC, hypothalamus, sciatic nerve, pancreas and white adipose tissue, but it had robust expression in the dorsal root ganglia (DRG) and trigeminal ganglia (Extended Data Fig. 2a). Outside the PNS, expression of peripherin was highest in the ileum, but with more than 31-fold lower expression relative to the trigeminal ganglion, and with even lower to absent expression in the cerebellum, cerebral cortex, midbrain, kidney, testis, pituitary, adrenal gland, stomach, duodenum, jejunum and colon (Extended Data Fig. 2b). Collectively, these data indicate that expression of peripherin is largely restricted to neurons of the PNS. In line with previous reports in rats showing that peripherin is expressed in only 46% of DRG neurons^39^, we find expression of *Gipr* decreased by ∽43% in the DRG of *Per-Cre*^*+*^*Gipr*^*flx/flx*^ mice (*Per-Gipr* KO) relative to *Per-Cre*^*+*^*Gipr*^*wt/wt*^ (WT) controls, and without differences in relative expression of *Gipr* in either the hypothalamus, hindbrain, sciatic nerve, epididymal white adipose tissue, pancreas, cerebellum, cerebral cortex, pituitary, kidney, duodenum, jejunum, ileum or colon (Extended Data Fig. 2c–p). Consistent with the high expression of peripherin in the trigeminal ganglia and the DRG (Extended Data Fig. 2a,b), we also confirmed *Per-Cre*-mediated deletion of *Gipr* in the trigeminal ganglion and the DRG in *Per-Gipr* KO mice using RNAscope (Extended Data Fig. 2q,r).

**Fig. 2:**
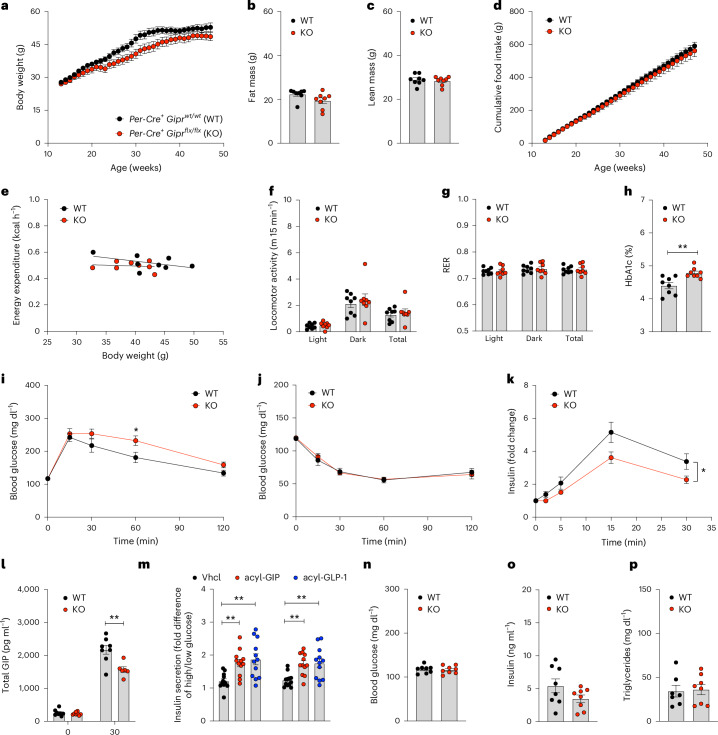
Metabolic phenotype of HFD-fed male *Per-Gipr* KO mice. **a**, Body weight development of male C57BL6/J *Per-Cre*^*+*^*Gipr*^*wt/wt*^ (WT) and *Per-Cre*^*+*^*Gipr*^*flx/flx*^ (KO) mice fed with a HFD (*n* = 8 each group). **b**,**c**, Fat (**b**) and lean (**c**) tissue mass of 44-week-old male WT and KO mice (*n* = 8 each group). **d**, Cumulative food intake of male WT and KO mice, measured per cage in double-housed mice from age 14 to 47 weeks (*n* = 8 each group). **e**–**g**, Energy expenditure (**e**), locomotor activity (**f**) and RER (**g**) of 49-week old male WT and KO mice (*n* = 8 each group). **h**,**i**, HbA1c in 46-week-old male WT and KO mice (*n* = 8 each group) (**h**), as well as glucose tolerance (**i**) after i.p. dosing with 1.5 g kg^−1^ glucose in 47-week-old male WT and KO mice (*n* = 7 each group). **j**, Insulin tolerance after i.p. dosing with 1.5 U kg^−1^ insulin (Humalog) in 48-week-old male WT and KO mice (*n* = 8 each group). **k**,**l**, Glucose-induced insulin secretion (*n* = 7 WT and *n* = 8 KO) (**k**) and corresponding levels of total GIP (*n* = 8 WT and *n* = 6 KO) (**l**) after oral glucose bolus administration of 4 g kg^−1^ glucose in 51-week-old male WT and KO mice. **m**, Insulin secretion, expressed as fold difference between high and low glucose (2.68 mM and 20 mM) in isolated islets from 46-week-old chow-fed male WT and KO mice treated with either vehicle or 50 nM of either native mouse GIP or GLP-1 (*n* = 12 independent biological samples per group). **n**–**p**, Fasting levels of blood glucose (**n**) and insulin (**o**) in 51-week-old male WT and KO mice (*n* = 8 each group), as well as triglycerides (**p**) in 52-week-old male WT (*n* = 7) and KO mice (*n* = 8). Data in **a**, **d** and **i**–**k** were analysed by two-way ANOVA with Bonferroni’s post hoc test for comparison of individual timepoints. Data in **b**, **c**, **h**, **i** and **n**–**p** were analysed using two-sided, two-tailed Student’s *t*-test. Data in **f** and **g** were analysed using a two-tailed, unpaired Mann–Whitney test. Data in **m** were analysed using a one-way ANOVA. Data in **e** were analysed using ANCOVA with body weight as the covariate. Cumulative food intake (**d**) was assessed per cage in *n* = 8 double-housed mice in each group. For data in **m**, handpicked islets of similar size were distributed per animal to achieve one well per treatment group (three wells per animal), each containing ten islets per well. Data represent mean ± s.e.m. *P < 0.05, **P < 0.01 and ***P < 0.001. Individual *P* values are shown in the Source data, unless *P* < 0.0001. Source data.

**Extended Data Fig. 3:**
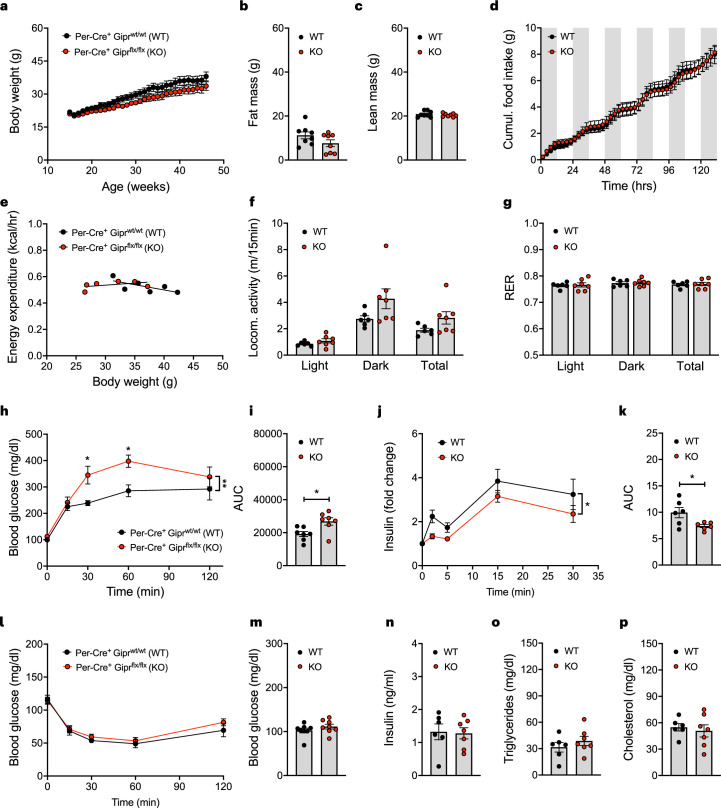
Metabolic phenotype of HFD-fed female *Per-Gipr* KO mice. Body weight development of female C57BL6/J *Per-Cre*^*+*^*Gipr*^*wt/wt*^ (WT) and *Per-Cre*^*+*^*Gipr*^*flx/flx*^ (KO) mice fed with a HFD (n = 8 each group) **(a)**. Fat and lean tissue mass of 35-week old female WT and KO mice (n = 8 each group) **(b,c)**. Cumulative food intake of 52-week old female WT (n = 6) and KO mice (n = 7) **(d)**. Energy expenditure **(e)**, locomotor activity **(f)** and respiratory exchange ratio (RER) **(g)** of 52-week old female WT (n = 6) and KO mice (n = 7). Glucose tolerance **(h)** and corresponding area under curve (AUC) **(i)** after i.p. dosing with 2 g/kg glucose in 48-week old female WT and KO mice (n = 7 each group). Glucose-induced insulin secretion **(j)** and corresponding AUC **(k)** after oral glucose bolus administration of 4 g/kg glucose in 54-week old female WT and KO mice (n = 6 each group). Insulin tolerance **(l)** after i.p. dosing with 0.75U/kg insulin (Humalog) in 50-week old female WT (n = 6) and KO mice (n = 8). Fasting levels of blood glucose (n = 8 each group) **(m)** and insulin (n = 6 WT, n = 7 KO) **(n)** in 54-week old female WT and KO mice, as well as fasting plasma levels of triglycerides (n = 6 WT, n = 7 KO) **(o)** and cholesterol (n = 6 WT, n = 7 KO) **(p)** in 55-week old female WT and KO mice. Data in panel **a,c,f** and **g** were analyzed by repeated measures 2-way ANOVA with Bonferroni’s post-hoc test for comparison of individual time points. Data in panel **f**, and **g**, were analyzed using Mann-Whitney test. Data in panel **a,d,e,h,j** and **l** were analyzed using 2-way ANOVA and with Bonferroni post-hoc comparison of individual time points. Data in panel **e** was analyzed using ANCOVA with body weight as covariate. Data in panel **b,c,i,k,m-p** were analyzed using Student’s two-tailed, two-sided ttest. Data represent mean ± SEM; asterisks indicate * p < 0.05; ** p < 0.01 and *** p < 0.001. Individual p-values are shown in the Data Source file, unless p < 0.0001. Source data

When fed a high-fat diet (HFD), male *Per-Gipr* KO mice showed no overt differences in body weight, body composition or food intake relative to WT controls (Fig. 2a–d). We further observed no differences in energy expenditure, locomotor activity or substrate utilization (Fig. 2e–g). However, we did find that DIO *Per-Gipr* KO mice had a higher glycated haemoglobin A1c (HbA1c) and slightly impaired glucose tolerance (Fig. 2h,i) with normal insulin sensitivity, but impaired secretion of insulin and GIP after oral bolus glucose administration compared with WT mice (Fig. 2j–l). The insulin secretory response to GIP and GLP-1 was, however, fully preserved in pancreatic islets isolated from WT and *Per-Gipr* KO mice (Fig. 2m), indicating that the impaired insulinotropic response observed in the *Per-Gipr* KO mice (Fig. 2k) did not result from impaired GIPR signalling in the islets. We also observed no differences in fasting levels of blood glucose, insulin or triglycerides (Fig. 2n–p). We also found that the metabolic phenotype of male DIO *Per-Gipr* KO mice was recapitulated in female DIO *Per-Gipr* KO mice, which, like male *Per-Gipr* KO mice, showed no difference in body weight, body composition, food intake, energy expenditure, locomotor activity or substrate utilization, but the females did have robust glucose intolerance with impaired glucose-induced insulin secretion, despite normal insulin tolerance and unchanged plasma levels of blood glucose, insulin, triglycerides and cholesterol (Extended Data Fig. 3a–p). Collectively, these data indicate that in both sexes, GIPR signalling in peripherin-expressing peripheral neurons is required for normal GIP and insulin responses to orally ingested glucose, but is not necessary for regulation of body weight, body composition or food intake.

### Metabolic effects of GLP-1R agonism–GIPR antagonism in DIO *Per-Gipr* KO mice

**Fig. 3:**
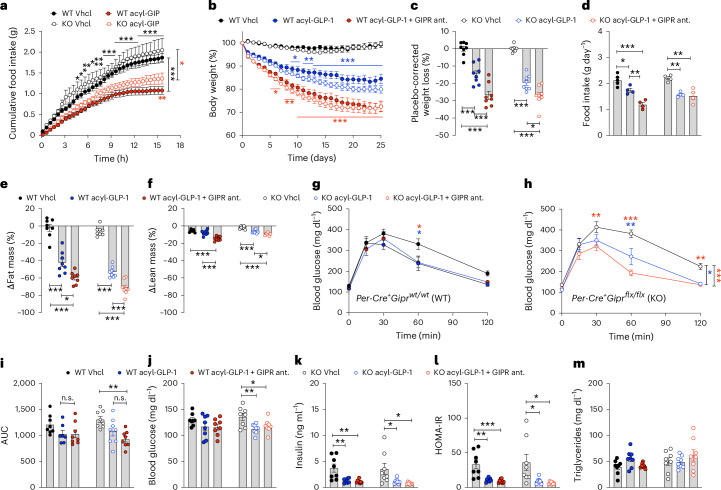
PNS-specific loss of *Gipr* has no effect on weight loss in DIO mice induced by GLP-1R agonism–GIPR antagonism co-therapy. **a**, Acute food intake of 49-week-old male C57BL/6J DIO *Per-Cre*^*+*^*Gipr*^*wt/wt*^ (WT) or *Per-Cre*^*+*^*Gipr*^*flx/flx*^ (KO) mice treated s.c. with a single dose of either vehicle (Vhcl) or acyl-GIP (100 nmol kg^−1^). **b**–**d**, Body weight development (**b**), placebo-corrected weight loss after 25 days treatment (**c**) and food intake (**d**) of 47-week-old male C57BL/6J WT and *Per-Gipr* KO mice treated daily with either vehicle, acyl-GLP-1 (10 nmol kg^−1^) or the combination of acyl-GLP-1 (10 nmol kg^−1^) and a GIPR antagonist (ant.) (1,500 nmol kg^−1^) (*n* = 8 each group). **e**,**f**, Body composition (fat mass (**e**) and lean mass (**f**), *n* = 8 each group) of 47-week-old male C57BL/6J DIO WT and *Per-Gipr* KO mice after 25 days of treatment. **g**–**i**, i.p. glucose tolerance (**g** and **h**) with corresponding area under curve (AUC) (**i**) in 47-week-old male C57BL/6J DIO WT (**g** and **i**) and *Per-Gipr* KO mice (**h** and **i**) after 25 days of treatment with either vehicle (*n* = 8 WT and *n* = 8 KO), acyl-GLP-1 (*n* = 7 WT and *n* = 8 KO) or the co-therapy of acyl-GLP-1 and the GIPR antagonist (*n* = 8 WT and *n* = 8 KO). **j**, Fasting plasma levels of blood glucose in 47-week-old male DIO WT and *Per-Gipr* KO mice treated either with vehicle, acyl-GLP-1 or the co-therapy of acyl-GLP-1 and the GIPR antagonist (*n* = 8 each group). **k**,**l**, Fasting plasma levels of insulin (**k**) and corresponding HOMA-IR (**l**) in 47-week-old male DIO WT and *Per-Gipr* KO mice treated either with vehicle (*n* = 8 WT and *n* = 8 KO), acyl-GLP-1 (*n* = 7 WT and *n* = 8 KO) or the co-therapy of acyl-GLP-1 and the GIPR antagonist (*n* = 8 WT and *n* = 8 KO). **m**, Ad libitum plasma levels of triglycerides in 47-week-old male DIO WT and *Per-Gipr* KO mice (*n* = 8 mice each group). Data in **a**, **b**, **g** and **h** were analysed by a two-way ANOVA with Bonferroni’s post hoc test for comparison of individual timepoints. Data in **c**–**f** and **i**–**m** were analysed using a one-way ANOVA. Cumulative food intake (**d**) was assessed per cage in *n* = 8 double- or single-housed mice each group. Data represent mean ± s.e.m. *P < 0.05, **P < 0.01 and ***P < 0.001. The asterisk colours in **a** correspond to the comparison of vehicle versus acyl-GIP in WT (black) and *Per-GIPR* KO (red) mice. The asterisk colours in **b** correspond to the comparison of acyl-GLP-1 versus the co-therapy in WT (blue) and *Per-GIPR* KO (red) mice. Individual *P* values are shown in the Source data, unless *P* < 0.0001. Source data.

**Extended Data Fig. 4:**
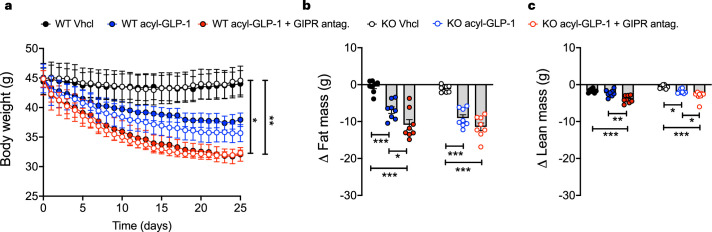
Absolute drug effects corresponding to Fig. 2. Body weight development of 47-week old male C57BL/6 J wildtype (WT) and *Per-Gipr* knockout (KO) mice treated daily with either vehicle, acyl-GLP-1 (10 nmolkg), or the combination of acyl-GLP-1 (10 nmol/kg) and a GIPR antagonist (1,500 nmol/kg) (n = 8 each group) **(a)**. Body composition (n = 8 each group) 47-wk old male C57BL/6 J DIO wildtype and *Per-Gipr* KO mice after 25 days of treatment **(b,c)**. Data in panel **a** was analyzed by 2-way ANOVA with Bonferroni’s post-hoc test for comparison of individual time points. Data in panel **b,c** were were analyzed using 1-way ANOVA. Data represent mean ± SEM; asterisks indicate * p < 0.05; ** p < 0.01 and *** p < 0.001. Individual p-values are shown in the Data Source file, unless p < 0.0001. Source data

Consistent with previous data showing that GIPR agonism acts in the CNS^15^ to decrease food intake via GABAergic GIPR neurons^14^, we found that the inhibition of food intake following single subcutaneous (s.c.) administration of acyl-GIP (100 nmol kg^−1^) was fully preserved in DIO *Per-Gipr* KO mice (Fig. 3a). Notably, however, we found that the co-therapy of acyl-GLP-1 (10 nmol kg^−1^) and the acylated GIPR antagonist (1,500 nmol kg^−1^) equally decreased body weight in DIO WT and *Per-Gipr* KO mice, with superiority of the co-therapy relative to treatment with acyl-GLP-1 alone (Fig. 3b,c). Expectedly, this effect is more clearly pronounced when expressing the data as per cent relative to absolute changes (Fig. 3b,c and Extended Data Fig. 4a).The co-therapy decreased food intake with comparable efficacy in WT and *Per-Gipr* KO mice, but with significance of the co-therapy over acyl-GLP-1 reached only in the WT mice (Fig. 3d). Mice treated with the co-therapy exhibited a greater decrease in fat and lean tissue mass relative to treatment with acyl-GLP-1, without an overt difference between WT and *Per-Gipr* KO mice (Fig. 3e,f and Extended Data Fig. 4b,c). In both WT and *Per-Gipr* KO mice, we found that the co-therapy improved glucose tolerance without superiority to GLP-1R agonism alone (Fig. 3g–i). Fasting levels of blood glucose were comparably decreased in mice treated with the co-therapy or acyl-GLP-1, but with significance reached only in the *Per-Gipr* KO mice (Fig. 3j). In both WT and *Per-Gipr* KO mice, we found that the fasting levels of insulin were decreased and insulin sensitivity increased after treatment with the co-therapy, but without superiority of the co-therapy to GLP-1R agonism alone (Fig. 3k,l). We observed no differences in either treatment or genotype regarding plasma levels of triglycerides (Fig. 3m). Collectively, these data show that the ability of GIPR antagonism to enhance GLP-1-induced weight loss is not mediated by GIPR signal inhibition in peripherin-expressing peripheral neurons. Furthermore, and consistent with our data in the *Vgat-Gipr* KO group (Fig. 1f–h), we found no major additional glycaemic benefits of the co-therapy relative to GLP-1R agonism alone (Fig. 3g–i).

### GIPR antagonist effects in mice global loss of *Gipr* or *Glp-1r*

**Fig. 4:**
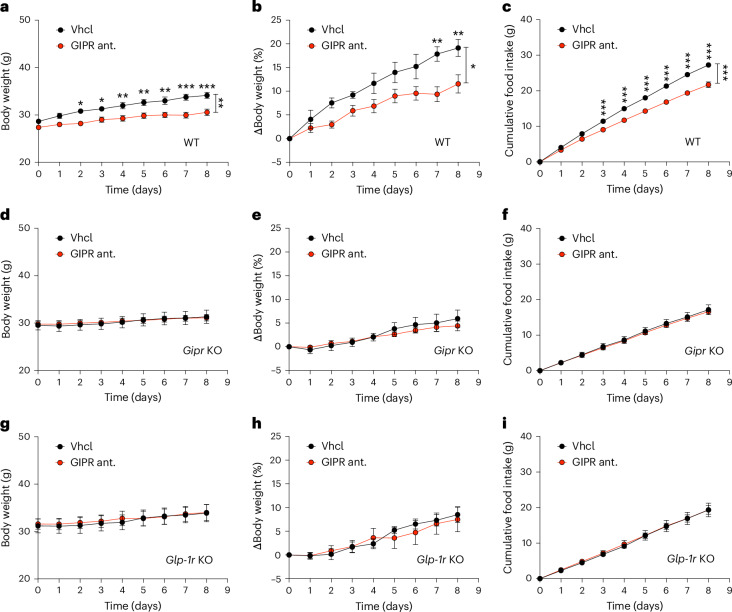
Global germline loss of either *Gipr* or *Glp-1r* blocks GIPR antagonism-mediated effects on body weight and food intake. **a**–**c**, Body weight in grams (**a**) and percent (**b**) and food intake (**c**) of HFD-fed 14–16-week-old male C57BL6/J WT mice treated s.c. with a single dose (30 mg kg^−1^) of either a control mAb (vehicle; *n* = 5) or an anti-GIPR antagonist (ant.) antibody (*n* = 6). **d**–**f**, Body weight in grams (**d**) and percent (**e**) and food intake (**f**) of HFD-fed 14–16-week-old male C57BL6/J global *Gipr* KO mice treated s.c. with a single dose (30 mg kg^−1^) of either a control mAb (vehicle) or an anti-GIPR antagonist antibody (*n* = 6 each group). **g**–**i**, Body weight in grams (**g**) and percent (**h**) and food intake (**i**) of HFD-fed 14–16-week-old male C57BL6/J global *Glp-1r* KO mice treated s.c. with a single dose (30 mg kg^−1^) of either a control mAb (vehicle) or an anti-GIPR antibody (*n* = 6 each group). Data represent mean ± s.e.m. *P < 0.05, **P < 0.01 and ***P < 0.001. Data in **a**–**i** were analysed by a two-way ANOVA with Bonferroni’s post hoc test for comparison of individual timepoints. Source data.

We next assessed the ability of a mouse GIPR neutralizing antibody^23^ (Kb of 5 nmol l^−1^, potency for antagonism of GIP-induced cAMP accumulation) to affect HFD-induced weight gain and food intake in lean mice kept at thermoneutrality (28 °C), an environmental temperature where potential confounding effects due to differences in metabolic rate are lowest. Interestingly, single s.c. treatment with the anti-GIPR antibody (30 mg kg^−1^) attenuated body weight gain and decreased food intake in lean WT mice (Fig. 4a–c), but not in mice with global loss of either *Gipr* (Fig. 4d–f) or *Glp-1r* (Fig. 4g–i). Of note, these data demonstrate that the body weight and food intake reducing effects of GIPR antagonism not only depend on functional GIPR signalling, but also on GLP-1R signalling. The latter contrasts with GIPR agonism, which we and others showed to exhibit a fully preserved ability to decrease body weight and food intake in *Glp-1r*-deficient mice^15,18^.

### GIPR agonism and antagonism have opposing effects in the DVC

**Fig. 5:**
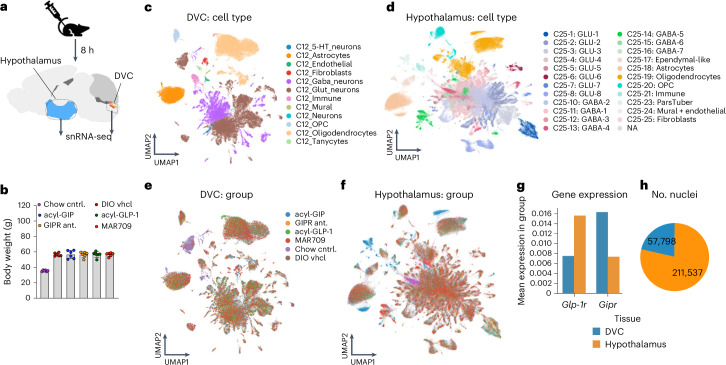
snRNA-seq of DVC and hypothalamus of mice treated with incretin mimetics. **a**,**b**, A schematic of the experimental design (**a**) and the body weight (**b**) of 36-week-old male C57BL/6J WT mice fed either a standard chow diet (cntrl.) or a HFD and treated s.c. with a single dose of either vehicle (vhcl), acyl-GIP (150 nmol kg^−1^), acyl-GLP-1 (50 nmol kg^−1^), an acylated peptide GIPR antagonist (1,500 nmol kg^−1^) or the GIPR:GLP-1R co-agonist MAR709 (50 nmol kg^−1^) (*n* = 6 each group). **c**–**f**, UMAP representations of gene expression coloured by C12-level cell type in the DVC (**c**) and C25-level cell type in hypothalamus (**d**), as well as by experimental group in the DVC (**e**) and hypothalamus (**f**). **g**, A bar graph showing mean expression of *Glp-1r* and *Gipr* in the DVC and hypothalamus. **h**, The number of nuclei isolated from each brain region. The colours correspond to log-normalized expression values scaled to the maximum of each gene. Data in **b** represent mean ± s.e.m. OPC, oligodendrocyte progenitor cell. Source data.

In contrast to our observation in *Per-Gipr* KO mice (Fig. 2a), we recently showed that body weight is decreased in HFD-fed mice with CNS-targeted loss of *Gipr*^15^, suggesting that the decrease in body weight that is induced by GIPR antagonism is mediated via neurons of the central rather than the peripheral nervous system. This is consistent with our observation that weight loss induced by GIPR antagonism depends on GLP-1R signalling (Fig. 4g–i), which also decreases body weight via central, rather than peripheral mechanisms^40^. To delineate the similarities and differences of GIPR (ant)agonism in the brain, we next performed snRNA-seq in the hypothalamus and the DVC, two regions implicated in regulation of food intake by GIPR agonism^20,21^, after single s.c. treatment of DIO mice with either vehicle, acyl-GIP^13–15^ (150 nmol kg^−1^), acyl-GLP-1 (50 nmol kg^−1^)^13–15^, the acylated peptide GIPR antagonist (1,500 nmol kg^−1^)^22^ or the GIPR:GLP-1R co-agonist MAR709 (50 nmol kg^−1^)^13–15^ (Fig. 5a,b). The rationale for assessing drug responses after acute treatment was to minimize confounding effects arising from differences in body weight after chronic drug treatment. Treatment groups largely overlapped across tissues, with comparable distribution of cell types, and with neurons constituting most of the captured nuclei across the treatment groups (Fig. 5c–f). We further found higher expression of *Gipr* in the DVC relative to the hypothalamus, while the opposite was found for the expression of *Glp-1r* (Fig. 5g). After exclusion of low-quality cells, we notably obtained RNA transcriptomes from 57,798 DVC and 211,537 hypothalamic nuclei (Fig. 5h).

**Fig. 6:**
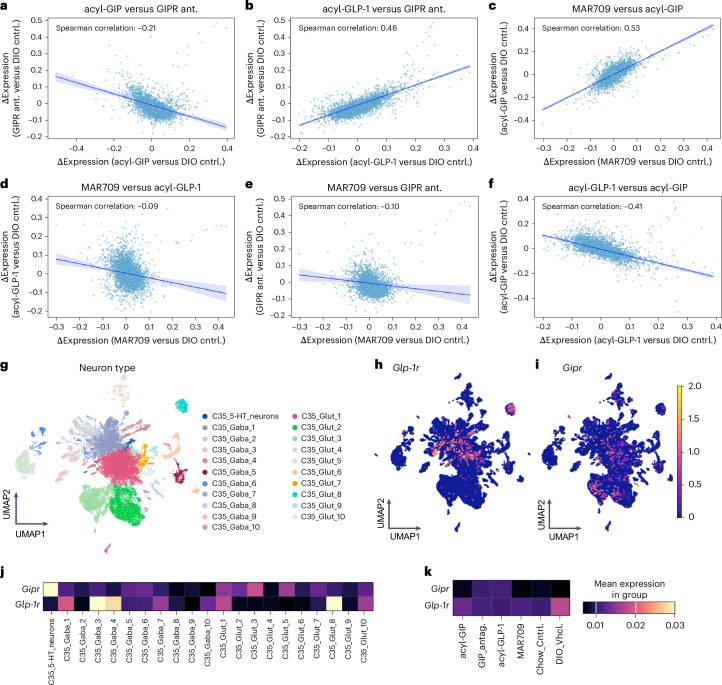
Correlation of drug-induced effects on DVC neuron gene expression differences. **a**–**f**, A comparison of log fold-change differences in gene expression in DVC neurons between male DIO C57BL/6J WT mice (DIO cntrl.) treated with acyl-GIP or the GIPR antagonist (ant.) (**a**), GIPR antagonist versus acyl-GLP-1 (**b**), acyl-GIP versus MAR709 (**c**), acyl-GLP-1 versus MAR709 (**d**), GIPR antagonist versus MAR709 (**e**) or acyl-GLP-1 versus acyl-GIP (**f**) (*n* = 6 mice per group, from which *n* = 3 mice were pooled to receive *n* = 2 independent biological replicates per group). **g**–**i**, UMAP representation of gene expression of DVC neurons coloured by neuron type (**g**), and with expression of *Glp-1r* (**h**) and *Gipr* (**i**). **j**,**k**, Heat maps showing mean gene expression of *Glp-1r* and *Gipr* in DVC neuronal populations (**j**) and experimental group (**k**), and with the colour corresponding to log-normalized expression values scaled to the maximum of each gene. Source data.

We found that DVC gene expression changes correlated negatively between mice treated with acyl-GIP or the GIPR antagonist (Fig. 6a), but positively between mice treated with acyl-GLP-1 versus the GIPR antagonist (Fig. 6b). These data indicate that GIPR antagonism triggers DVC transcriptional responses like those of GLP-1R agonism, and further corroborate that GIPR agonism and antagonism decrease body weight and food intake via different mechanisms. These data are further in agreement with our observation in vivo showing that GIPR antagonism, unlike GIPR agonism^15,18^, depends on functional GLP-1R signalling to decrease body weight and food intake (Fig. 4g–i). Expectedly, a strong positive correlation in gene expression changes was observed in mice treated with MAR709 versus acyl-GIP (Fig. 6c), but notably not with MAR709 versus acyl-GLP-1 (Fig. 6d) or MAR709 versus GIPR antagonism (Fig. 6e). These data are consistent with the established role of MAR709 as a potent GIPR agonist^13–15,41^, and indicate that neither acyl-GIP nor the GIPR:GLP-1R co-agonist MAR709 works as a functional GIPR antagonist. In line with this is our further observation that gene expression changes correlate positively between mice treated with acyl-GLP-1 versus the GIPR antagonist (Fig. 6b), but negatively between mice treated with acyl-GLP-1 versus acyl-GIP (Fig. 6f). Notably, the observation that DVC gene expression changes are stronger in mice treated with MAR709 versus acyl-GIP (Fig. 6c) relative to mice treated with MAR709 versus acyl-GLP-1 (Fig. 6d) indicates that GIPR is the primary target of MAR709 in the DVC, with fewer transcriptional changes induced by MAR709 via GLP-1R. In agreement with this notion, we found that expression of *Glp-1r* concentrated in specific neuronal populations, which include two GABAergic neuronal clusters (C35 GABA3 and C35 GABA4) and one glutamatergic neuronal cluster (C35 Glut 8) (Fig. 6g), while expression of *Gipr* is more broadly distributed across DVC neuronal populations, with particularly high expression in a small population of 5-HT-positive neurons (Fig. 6g–j). We observed no large differences in *Gipr* and *Glp-1r* expression across experimental groups (Fig. 6k).

### GIPR antagonism and GLP-1R agonism both modulate DVC gene programmes implicated in synaptic plasticity

**Fig. 7:**
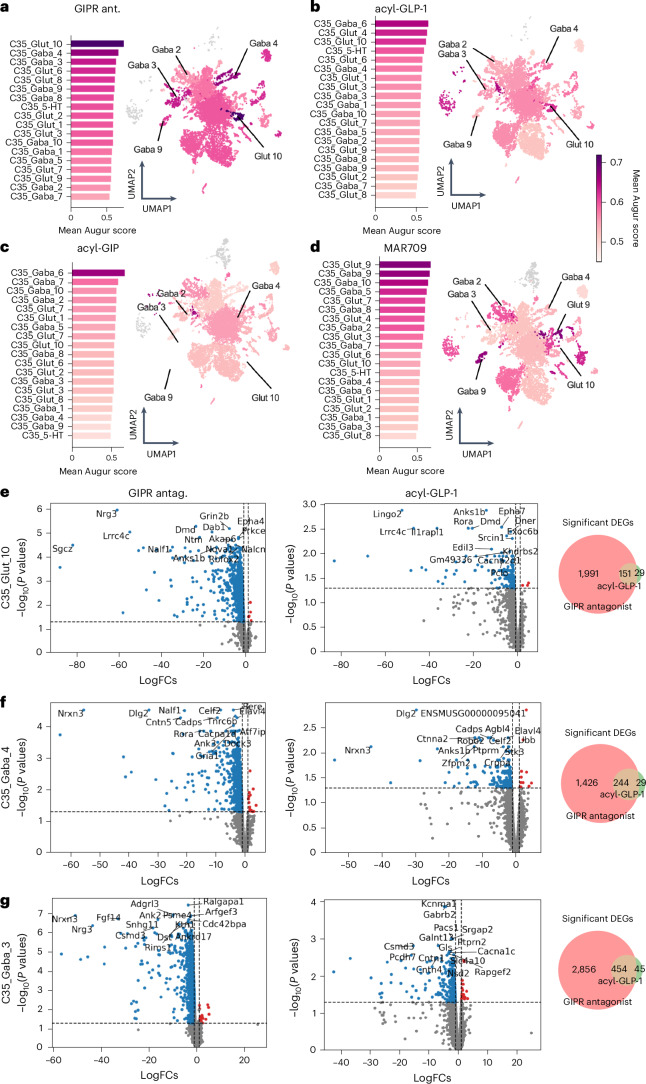
Differential effects of GIPR (ant)agonism on DVC neuronal cell types. **a**–**d**, Bar plots and UMAP representations of gene expression in DVC neurons of male DIO mice treated with a GIPR antagonist (ant.) (**a**), acyl-GLP-1 (**b**), acyl-GIP (**c**) or MAR709 (**d**) (*n* = 6 mice per group, from which *n* = 3 mice were pooled to receive *n* = 2 independent biological replicates per group). The bar plots and UMAPs are coloured by Augur score, representing cell type-specific changes in gene expression of the treatment group relative to DIO vehicle controls. **e**–**g**, Volcano plots (log_2_ fold change (FC) versus adjusted *P* values from a two-sided Wilcoxon rank-sum test, corrected for multiple comparison) of differentially expressed genes (DEGs) following treatment with either the GIPR antagonist or acyl-GLP-1 in the top GIPR antagonist affected neuronal clusters Glut10 (**e**), GABA4 (**f**) and GABA3 (**g**). Only the top 15 DEGs are highlighted. Venn diagrams show the overlap of significant DEGs (adjusted *P* < 0.05) from GIPR antagonist and acyl-GLP-1 groups. *P* values of DEGs were obtained by Wilcoxon rank-sum tests and were adjusted for multiple comparisons using the Benjamini–Hochberg method. Source data.

We next performed cell-type prioritization analysis using Augur^42^ to determine which types of neurons were most affected by individual drug treatment in the DVC (Fig. 7a–d). The higher the Augur score, the more information about group identity is embedded in its gene expression profile, indicating a greater change in gene expression in response to drug treatment. Notably, we found that among the three neuronal populations that were most affected by GIPR antagonism are the two main *Glp-1r*-expressing clusters C35 GABA4 and C35 GABA3, but with C35 Glut10 neurons being the most affected (Fig. 7a). These same neuronal populations also ranked high (sixth, ninth and third, respectively) after treatment with acyl-GLP-1 (Fig. 7b), but ranked low following treatment with acyl-GIP or MAR709 (Fig. 7c,d). Collectively, these data again suggest that GIPR antagonism, unlike agonism, mimics GLP-1R agonism in the DVC and that GIPR, unlike GLP-1R, is the primary target for MAR709 in the DVC.

We next compared the differentially expressed genes induced by either GIPR antagonism or GLP-1R agonism in the C35 Glut10, C35 GABA4 and C35 GABA3 clusters (Fig. 7e–g), that is, in the three neuronal populations most affected by GIPR antagonism (Fig. 7a). In all three neuronal clusters, we found a similar pattern of gene expression changes after treatment with the GIPR antagonist and acyl-GLP-1, with 29 genes in the C35 Glut10 cluster, 29 genes in the C35 GABA4 cluster and 45 genes in the C35 GABA3 cluster affected by both GLP-1R agonism and GIPR antagonism (Fig. 7e–g). Most of the genes downregulated by GLP-1R agonism and GIPR antagonism were associated with neural plasticity and synapse formation, including neuregulin 3 (*Nrg3*), neurexin 3 (*Nrxn3*), discs large MAGUK scaffold protein 2 (*Dlg2*), sodium leak channel, non-selective (*Nalcn*), neurotrimin (*Ntm*), leucine rich repeat and Ig domain containing 2 (*Lingo2*), leucine rich repeat containing 4C (*Lrrc4c*), interleukin 1 receptor accessory protein like 1 (*Il1rapl1*) and glutamate ionotropic receptor NMDA type subunit 2B (*Grin2b*) (Fig. 7e–g). Notably, *Nrxn3*, which encodes for a synaptic adhesion protein critical for maintaining synaptic function^43^, was downregulated by GIPR antagonism and GLP-1R agonism in both the C35 GABA4 and the C35 GABA3 cluster (Fig. 7f,g), while *Nrg3*, which regulates excitatory synapse formation^44^, was strongly downregulated in C35 Glut10 and C35 GABA3 neurons by GIPR antagonism but not by GLP-1R agonism (Fig. 7e,g). Furthermore, we found that *Lrrc4c* and *Il1rapl1*, both of which encode for factors that are involved in excitatory synapse formation^45–47^, were downregulated by GIPR antagonism and by GLP-1R agonism in the C35 Glut10 cluster (Fig. 7e). In summary, these data not only show that GIPR antagonism and GLP-1R agonism act on the same neuronal populations in the DVC, but also that they similarly downregulate gene programmes implicated in synaptic plasticity and synapse formation.

### GIPR antagonism does not mimic GLP-1R agonism in the hypothalamus

**Fig. 8:**
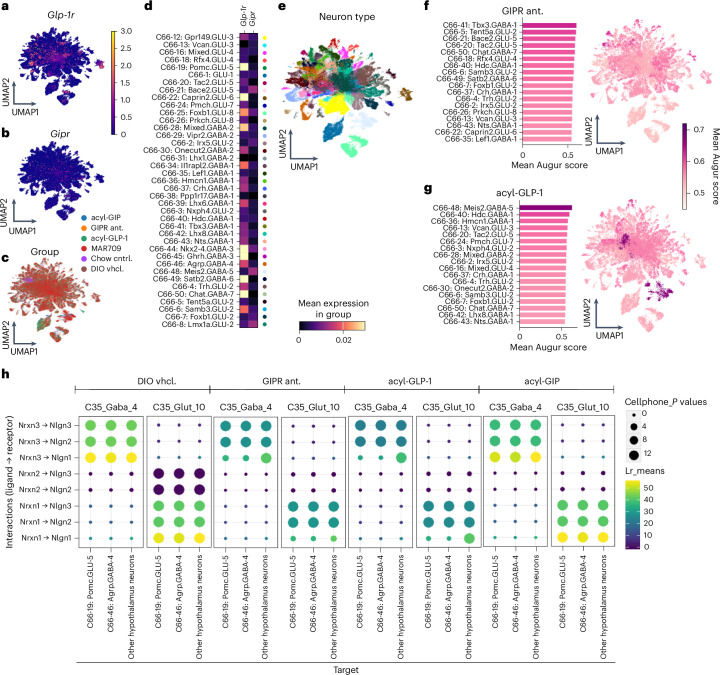
Effect of GIPR antagonism versus acyl-GLP-1 on hypothalamic gene expression and signalling from the DVC. **a**–**d**, UMAP representation of hypothalamic neuronal gene expression coloured by expression of *Glp-1r* (**a**), *Gipr* (**b**), experimental group (**c**) or C66-level neuron type (**d**). **e**, Heat maps showing *Glp-1r* and *Gipr* mean gene expression in hypothalamic neuronal types. **f**,**g**, Bar plots and UMAP representations of gene expression in DVC neurons of DIO mice treated with a GIPR antagonist (**f**) or acyl-GLP-1 (**g**). The bar plots are ranked by, and UMAPs and bar plots are coloured by Augur score, representing cell-specific change in gene expression of the experimental group versus vehicle DIO controls (**f** and **g**). **h**, The top ten most likely cell–cell communication events between DVC GABA4 or Glut 10 neurons and hypothalamic C66-19: Pomc.GLU-4; C66-46: Agrp.GABA-4 or all other hypothalamic neurons in DIO mice treated with vehicle, the GIPR antagonist, acyl-GLP-1 or acyl-GIP. Cellphone *P* values are permutation-based *P* values. Lr, ligand–receptor expression. Source data.

**Extended Data Fig. 5:**
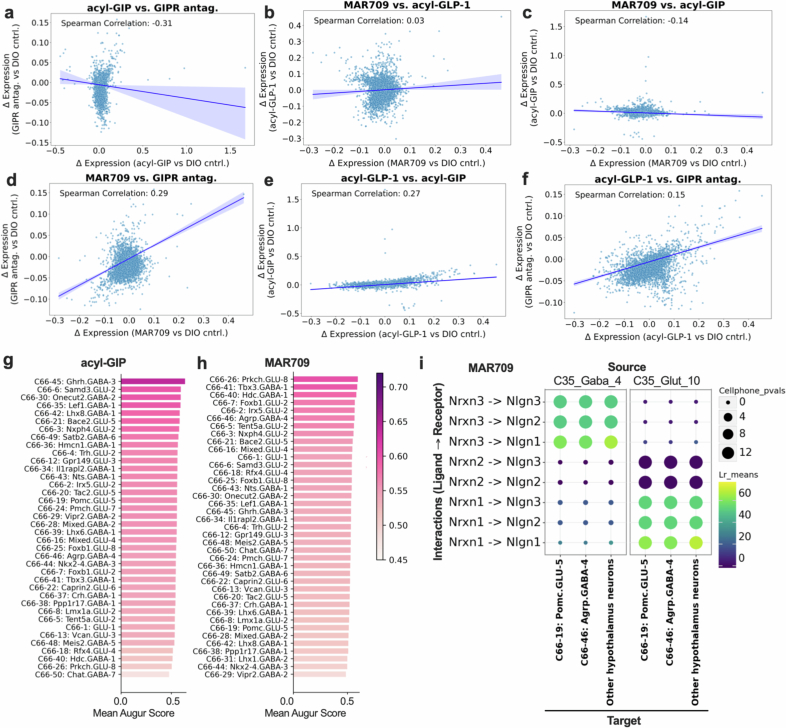
Correlation of drug-induced effects on hypothalamic gene expression differences. Comparison of Log fold change differences in gene expression between male DIO C57BL/6 J wildtype mice treated with acyl-GIP or the GIPR antagonist **(a)**, MAR709 vs. acyl-GLP-1 **(b)**, MAR709 vs. acyl-GIP **(c)**, MAR709 vs. the GIPR antagonist **(d)**, acyl-GLP-1 vs. acyl-GIP **(e)**, or acyl-GLP-1 vs. the GIPR antagonist **(f)** (n = 6 mice per group, from which n = 3 mice were pooled to receive n = 2 independent biological replicates per group). Bar plots are ranked Augur score in mice treated with either acyl-GIP **(g)** or MAR709 **(h)**, representing cell-specific change in gene expression of the respective groups relative to Vehicle-treated DIO controls. The top 10 most likely cell-cell communication events between DVC GABA4 or Glut 10 neurons and hypothalamic C66-19: Pomc.GLU-4, C66-46: Agrp.GABA-4, or all other hypothalamic neurons in mice treated with MAR709 **(i)**. Cellphone p-values are permutation-based p-values, Lr means are mean ligand-receptor expression. Source data

We next turned our attention to the hypothalamus. Here, we found only low expression of *Gipr* across all neuronal types, while *Glp-1r* was more robustly expressed; particularly, and keeping with the HypoMap^48^ annotations, in C66-19: Pomc.GLU-5; C66–22: Caprin2.GLU-6; C66–41: Nkx2-4.GABA-3; C66–45: Ghrh.GABA-3; C66–49: Satb2.GABA-6 and C66–50: Chat.GABA-7 neurons (Fig. 8a–e). In contrast to our observations in the DVC (Fig. 7a,b), cell type prioritization analysis revealed that neuron types with high *Glp-1r* expression do not consistently have the largest changes in gene expression after treatment with either the GIPR antagonist or acyl-GLP-1 (Fig. 8f,g). This observation aligns also with a generally lower correlation in gene expression in hypothalamic neurons of both mice treated with the GIPR antagonist or acyl-GLP-1, and further in mice treated with acyl-GIP or MAR709 (Extended Data Fig. 5a–f). Notably, we found that C66–48: Meis2.GABA-5 neurons are the most affected by GLP-1R agonism, and C66–45: Ghrh.GABA-3 neurons are most affected by GIPR agonism, but both of these populations were less affected after treatment with the GIPR:GLP-1R co-agonist MAR709 (Extended Data Fig. 5g,h).

To infer whether the observed changes in transcriptional gene programmes indicative of reduced synaptic plasticity by GIPR antagonism and GLP-1R agonism in the DVC translate to altered signalling in the hypothalamus, we performed a cell–cell communication analysis using the LIANA+^49^ implementation of the CellPhoneDB^50^ algorithm, and the receptor–ligand database from NeuronChat^51^ (Fig. 8h). Cell–cell communication analysis in dissociated single-cell data infers likely communication events from the expression of known ligand–receptor pairs across different cell types to predict interactions based on transcriptomic profiles. We found similar alterations in the probability of cell–cell communication events between C35 GABA4 and C35 Glut10 sender neurons and hypothalamic receiver neurons after treatment with acyl-GLP-1 or the GIPR antagonist compared with vehicle DIO controls, which clearly diverge from that of acyl-GIP (Fig. 8h). Treatment with the GIPR antagonist and acyl-GLP-1 both led to a reduction in the likelihood of *Nrxn3-Nlgn1* signalling between C35 GABA4 neurons and Pomc.GLU-5 and Agrp.GABA-4 neurons, a change that was specific to these feeding-related neurons and absent in other hypothalamic neurons (Fig. 8h). Similarly, we observed a decrease in the likelihood of *Nrxn1-Nlgn1* signalling from C35 Glut10 neurons to Pomc.GLU-5 and Agrp.GABA-4 neurons (Fig. 8h). We did not observe a difference between Pomc.GLU-5 and Agrp.GABA-4 neurons and all other hypothalamic neurons in these signalling pathways in mice treated with acyl-GIP or MAR709 (Fig. 8h and Extended Data Fig. 5i). Together, these data suggest that GLP-1R agonism and GIPR antagonism may exert their effects on energy balance by downregulating signalling from DVC C35 GABA4 and C35 Glut10 neurons to hypothalamic feeding circuits.

**Extended Data Fig. 6:**
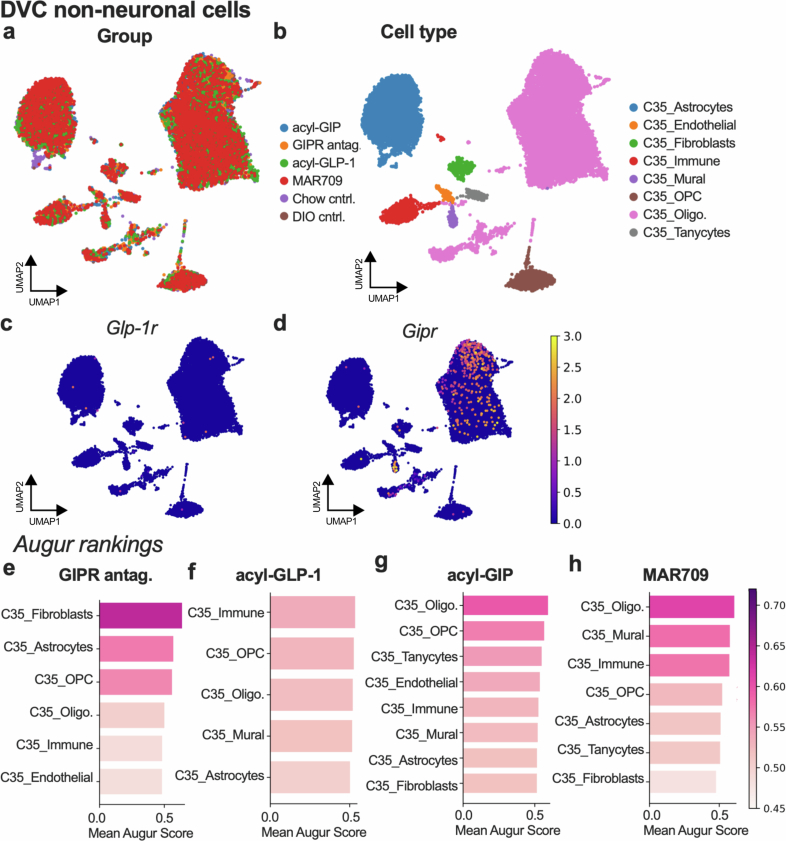
Drug-induced effects on DVC non-neuronal cells. UMAP representations of gene expression of DVC non-neuronal cells colored by experimental group **(a)**, cell type **(b)**, or expression of either *Glp-1r* **(c)** or *Gipr* **(d)**. Bar plots with Augur scores of DVC non-neuronal cells from mice treated with either the GIPR antagonist **(e)**, acyl-GLP-1 **(f)**, acyl-GIP **(g)**, or MAR709 **(h)**. Bar plots are ranked and colored by Augur score, representing cell-specific change in gene expression of the respective group relative to vehicle-treated DIO controls. Source data

**Extended Data Fig. 7:**
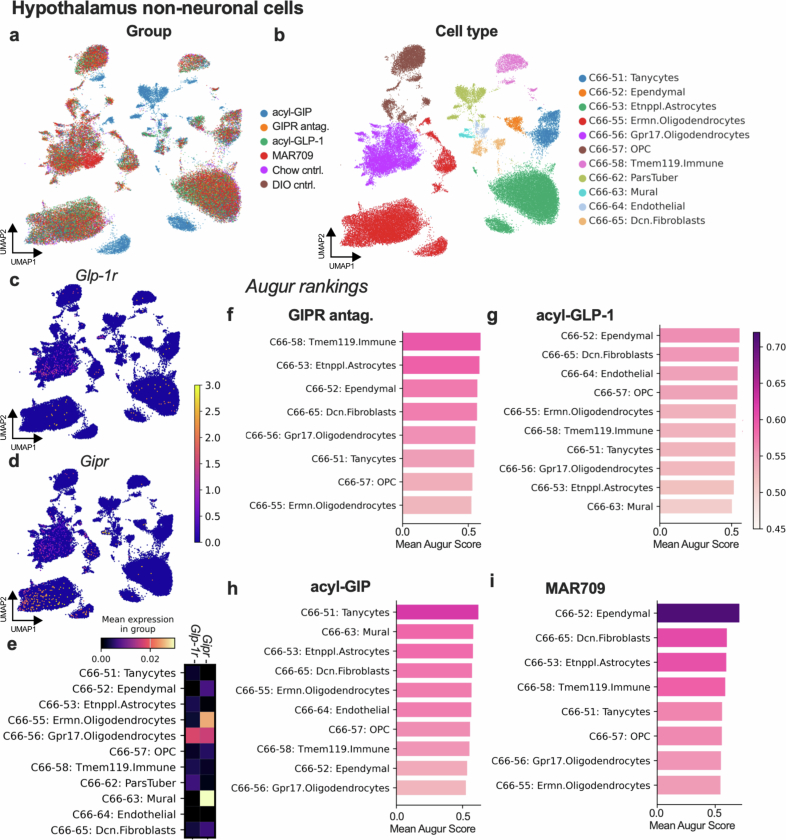
Drug-induced effects on hypothalamus non-neuronal cells. UMAP representations of gene expression of hypothalamus non-neuronal cells colored by experimental group **(a)**, cell type **(b)**, or expression of *Glp-1r* **(c)**, or Gipr **(d)**. **(e)** Heatmap showing *Glp-1r* and *Gipr* mean gene expression in hypothalamic non-neuronal cell types. Color corresponds to log-normalized expression values scaled to the maximum of each gene. Bar plots showing Augur scores of hypothalamic non-neuronal cells from mice treated with the GIPR antagonist **(f)**, acyl-GLP-1 **(g)**, acyl-GIP **(h)**, or MAR709 **(i)**. Bar plots are ranked and colored by Augur score, representing cell-specific change in gene expression of the respective group relative to vehicle-treated DIO controls. Source data

In non-neuronal cells, we found in the DVC the highest expression of *Gipr* in oligodendrocytes (Extended Data Fig. 6a–d), which further constituted the most affected cell type in this area after treatment with either acyl-GIP or MAR709 (Extended Data Fig. 6e–h). In contrast to this, while non-neuronal *Gipr* expression was also in the hypothalamus highest in oligodendrocytes (Extended Data Fig. 7a–e), this cell type was, in this area, among the least affected after treatment with either acyl-GIP or MAR709 (Extended Data Fig. 7f–i). We also found tanycytes and ependymal cells among the most affected cell types in all treatment groups in the hypothalamus, that is, cell types with privileged access to the third ventricle and which have previously been implicated in the food intake inhibitory effects of the GLP-1R agonist liraglutide^52^.

**Extended Data Fig. 8:**
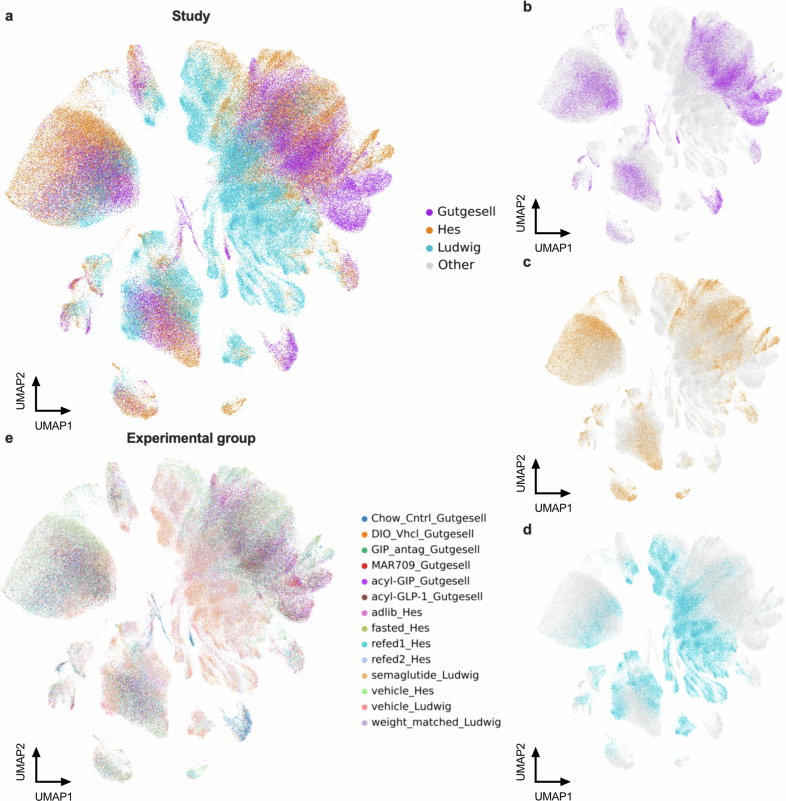
Integration with public snRNAseq DVC datasets. UMAP representation of an scVI joint embedding showing all DVC nuclei from this study (Gutgesell), with all nuclei from the Hes et al.^53^ and Ludwig et al.^54^ datasets **(a)**, and individual UMAPs showing each individual study: Gutgesell **(b)**, Hes et al. **(c)**, Ludwig et al.^54^ **(d)**, and by the experimental group from each study **(e)**. Source data

To compare cell types with the neuronal populations we identified, we integrated our DVC data with two publicly available murine DVC datasets from Hes et al.^53^ and from Ludwig et al.^54^. This presented a particular challenge as, in addition to the expected variation from different laboratories, each dataset had been produced using different experimental groups. As each dataset has multiple experimental groups, to correct for the variance between studies while preserving variance between cell types and experimental groups, we trained the single-cell variational inference (scVI) model^55^ on the control groups from each study with the study as the batch key, and then integrated the experimental groups (Extended Data Fig. 8a–d). Most experimental groups from all three datasets integrated well, however, neurons from mice treated once daily with semaglutide in the Ludwig^54^ dataset did not integrate well, suggesting that longer-term administration of GLP-1R agonists continue to have a large impact on DVC neuron cell state after the initial dose, although this difference may be confounded by the reduction in body weight (Extended Data Fig. 8e).

**Extended Data Fig. 9:**
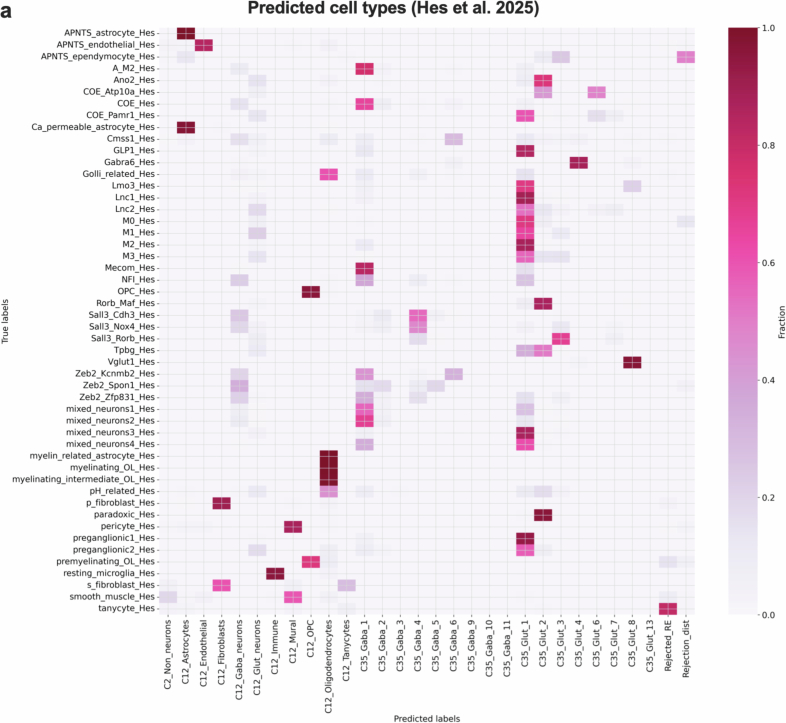
Predicted cell types from Hes et al.^53^. Pairwise heatmap showing the proportion of cells labeled by Hes et al. (y-axis), predicted to belong to each DVC cell-type from this study (x-axis) using scHPL. Source data

**Extended Data Fig. 10:**
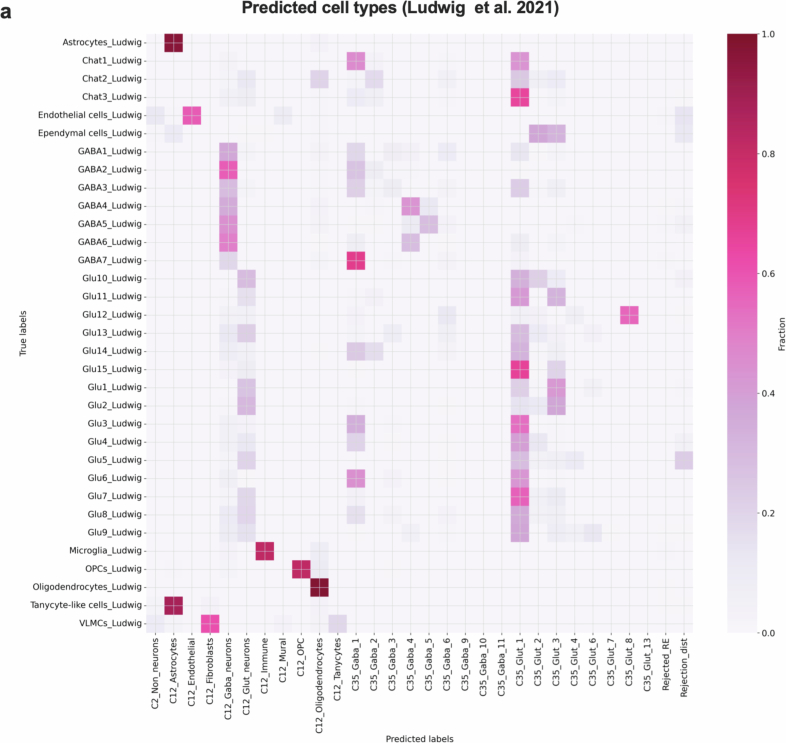
Predicted cell types from Ludwig et al.^54^. Pairwise heatmap showing the proportion of cells labeled by Ludwig et al. (y-axis), predicted to belong to each DVC cell-type from this study (x-axis) using scHPL. Source data

To validate our cell typology framework, we predicted the cell-type labels from our framework using progressive learning through scHPL^56^ and compared them to the author-provided cell types for both the Hes^53^ and Ludwig^54^ datasets (Extended Data Figs. 9a and 10a). We observed good concordance of major cell types at the C12 annotation level between our data and both the Hes^53^ and Ludwig^54^ datasets; however, at the C35 level, most neuron subclusters mapped to the largest glutamatergic or GABAergic neuron cluster, probably owing to the persistent differences between cells from the different experimental conditions.

## Discussion

In this study, we assessed the effect on energy metabolism by GIPR antagonism in several mouse lines with global or targeted deficiency of *Gipr* or *Glp-1r*. We further delineated the transcriptional similarities and differences of GIPR (ant)agonism, GLP-1R agonism and GIPR:GLP-1R co-agonism in the hypothalamus and DVC of DIO mice using snRNA-seq analysis. Similar to GIPR agonism^15,18^, we found the reduction of body weight and food intake caused by GIPR antagonism was eliminated in mice with global loss of *Gipr*. However, while we and others previously showed that GIPR agonism remains fully efficacious to decrease body weight and food intake in mice deficient for *Glp-1r*^15,18^, here we found that loss of *Glp-1r* renders mice resistant to weight loss and inhibition of food intake by GIPR antagonism. Furthermore, while we and others previously showed that GIPR agonism decreases body weight and food intake via *Gip*r signalling in GABAergic neurons^14,19^, here we found that the ability of GIPR antagonism to amplify GLP-1-induced weight loss does not depend on the presence of GIPR in GABAergic neurons. Likewise, we show that the ability of GIPR antagonism to enhance GLP-1-induced weight loss is also preserved in mice with peripherin-*Cre*-mediated loss of *Gipr* in the PNS. We should note here that the preservation of weight loss and food intake inhibition by GIPR antagonism in mice with disturbed GIPR signalling in the PNS is not unexpected, given that mice with CNS loss of *Gipr* (thus, mimicking the use of an antagonist) show decreased body weight and food intake when fed a HFD^15^, suggesting that the reduction in body weight by GIPR antagonism is mediated by neurons of the central rather than peripheral nervous system. Consistent with this is also our observation that the body weight-lowering effects of GIPR antagonism depend on GLP-1R signalling, which likewise are mediated via central rather than peripheral, mechanisms^40^. Relevant brain areas implicated in GLP-1 control of body weight and food intake include the the hypothalamic arcuate nucleus^57–59^ and the hindbrain DVC^59,60^, hence the same brain regions that are targeted by long-acting GIPR agonists^15,20,21^. Notably, the same brain regions are also targeted by the bispecific GIPR antagonist-GLP-1R agonist antibody, GIPR-Ab/GLP-1, as shown in an accompanying manuscript by Liu et al.^61^. In that study, the authors further utilized pharmacology and mouse genetics to provide complementary evidence supporting a role for attenuation of CNS GIPR signalling in the enhancement of the weight loss effects induced by the GLP-1R agonist dulaglutide^61^. Furthermore, weight loss achieved using GIPR-Ab/GLP-1 was attenuated in mice with CNS loss of either *Gipr* or *Glp-1r*^61^. Collectively, these findings are consistent with the major experimental findings described herein, and further corroborate that GIPR antagonism acts centrally to amplify GLP-1-induced weight loss. Nonetheless, although mice with GIPR signal inhibition in the PNS do not show alterations in body weight and remain fully sensitive to weight loss induced by GIPR antagonism, these mice develop glucose intolerance with impaired glucose-induced secretion of insulin and GIP when fed a HFD. We hence establish a crucial role of GIPR signalling in peripheral neurons in the regulation of glucose homeostasis, but not body weight, under conditions of diet-induced obesity. Collectively, our data show that GIPR agonism and antagonism decrease body weight and food intake via different neuronal mechanisms, with GIPR antagonism, unlike agonism, depending on GLP-1R signalling but not GIPR signalling in either GABAergic or peripheral neurons.

In agreement with this finding, our snRNA-seq analysis revealed that GIPR antagonism, but not agonism, mimics GLP-1R agonism in the DVC. DVC neuronal gene expression changes correlate negatively in mice after treatment with GIPR agonism versus antagonism, but positively in mice treated with GIPR antagonism versus GLP-1R agonism. We observed the greatest transcriptional changes induced by GIPR antagonism in the C35 GABA4, C35 GABA3 and C35 Glut10 neurons, which were also among the highest affected neuronal populations targeted by GLP-1R agonism, but not by GIPR agonism. Interestingly, within these neuronal clusters, GIPR antagonism and GLP-1R agonism are separated from GIPR agonism in that they both similarly downregulate gene programmes indicative of neuronal plasticity and synapse formation. These findings further support the notion that GIPR antagonism and GLP-1R agonism are functionally related and act similarly on DVC neurons, and in a clearly distinct manner from GIPR agonism. In summary, we show here that GIPR agonism and antagonism affect body weight and food intake via different, rather than similar mechanisms, with GIPR antagonism affecting body weight and food intake via modulation of GLP-1R signalling. The observation that gene expression changes induced by GIPR agonism versus its antagonism correlate negatively further argues that our GIPR agonist is not a functional antagonist.

It warrants clarification as to how GIPR antagonism decreases body weight in a GLP-1R-dependent manner. The observation that the body weight-lowering effect of GIPR antagonism vanishes in mice with global deletion of both *Gipr* and *Glp-1r* potentially points to an inhibitory mechanism by which non-GABAergic GIPR^+^ neurons partially silence GLP-1R^+^ neurons so that the latter are less than maximally efficacious. Antagonization of these GIPR^+^ neurons may thus either directly or indirectly derepress the action of downstream GLP-1R^+^ neurons to further decrease body weight and food intake. Notably, like previous studies^21^, we here find expression of *Gipr* enriched in 5-HT neurons. Given their established role in regulating hunger and satiety^62,63^ and the recent demonstration that the 5-HT2C receptor agonist lorcaserin acts on brainstem GLP-1 neurons to augment food intake suppression by GLP-1R agonism^64^, it seems plausible to hypothesize that weight loss induced by GIPR signal modification may involve modulation of the hypothalamic and/or hindbrain serotonergic system.

Limitations of our study include that peripherin*-Cre* does not target all neurons of the PNS. We hence cannot exclude the possibility that peripherin-negative neurons of the PNS play a functional role in the metabolic effects of GIPR antagonism. Since expression of peripherin is not fully exclusive for the PNS, we further cannot exclude the possibility that GIPR was also deleted in our studies in peripherin-expressing neurons outside the PNS. Different molecules with GIPR (ant)agonism may further differ in their pharmacokinetics, including their biodistribution and brain penetrance, which may affect their mode of action in the brain and the periphery. The lack of commonly available and sufficiently selective antibodies to detect GIPR further remains a notable limitation that hinders in-depth immunohistochemical analysis of GIPR in the brain. Notably, expression of drug effects appears generally more robust when comparing relative as compared with absolute values, which is a common problem in biomedical sciences that resides in the typically observed greater data variability in absolute versus relative data. Another limitation of our study is that we only compared drug effects using snRNA-seq after single acute drug treatment, hence not allowing conclusions on transcriptomic changes after more chronic treatment. Further limitations are that the Vgat-Gipr KO and WT mice differ in their starting body weight, which urges caution when comparing drug-induced effects across genotypes. We further only demonstrated the GLP-1R-dependent body weight-lowering effect of GIPR antagonism in mice with global deletion of GLP-1R and GIPR. It warrants clarification whether this effect holds true also in mice with more CNS-targeted deletion of GIPR and GLP-1R. We further only tested drug effects in DIO and glucose intolerant male mice, since female mice are largely resistant to development of diet-induced obesity and glucose intolerance^65^. It should further be noted that measures of drug effects on body weight are generally more robust than changes in food intake, since mice often have a tendency to shred their food, which if unnoticed, may contribute to a certain degree of bias in the analysis. To not interfere with drug-induced body weight effects, we could further only measure glucose tolerance at the end of the study. Since instant assessment of insulin tolerance using an intraperitoneal (i.p.) insulin tolerance test was not possible due to animal ethics reasons, we were further only able to measure insulin sensitivity using the HOMA-IR, which nonetheless correlates well with direct measures of insulin sensitivity using either i.p. insulin tolerance test or clamps^66–69^.

## Methods

### Animals and housing conditions

Experiments were performed in accordance with the Animal Protection Law of the European Union after permission by the Government of Upper Bavaria, or the Eli Lilly and Company Institutional Animal Care and Use Committee. Mice were double or single housed and, unless otherwise indicated, fed ad libitum with either a regular chow (1314, Altromin) or a HFD (58% fat, D12331, Research Diets) diet under constant ambient conditions of 22 ± 2 °C with constant humidity (45–65%) and a 12 h/12 h light/dark cycle. C57BL/6J *Vgat-ires-cre* knock-in mice were purchased from The Jackson Laboratory (028862) and crossed with C57BL6/J *Gipr*^flx/flx^ mice^35,36^ to generate *Vgat-cre*^+/−^Gipr^flx/flx^ (*Vgat-Gipr* KO) mice and *Vgat-cre*^+/−^Gipr^wt/wt^ (WT) controls. *Per-Cre* mice^37^ (MGI ID:3841120) were crossed with C57BL/6J mice for >10 generations before pairing with C57BL6/J *Gipr*^*flx/flx*^ mice^35,36^ to receive *Per-cre*^*+/−*^*Gipr*^*flx/flx*^ (*Per-Gipr* KO) mice and *Per-cre*^+/−^Gipr^wt/wt^ (WT) controls. Body composition was analysed using a magnetic resonance whole-body composition analyser (EchoMRI).

### Pharmacological studies

For assessment of drug effects under room temperature (22 ± 2 °C), male age-matched mice were double housed and fed with a 58% HFD (D12331, Research Diets) for approximately 20 weeks, followed by random assignment into groups of matched genotype, body weight and body composition. Mice were treated at the indicated doses with either long-acting acyl-GIP (IUB0271)^13–15^, acyl-GLP-1 (IUB1746)^13–15^, the GIPR:GLP-1R co-agonist MAR709 (refs. ^13–15^) or an acylated peptide GIPR antagonist ([N^α^-Ac,L^14^,R^18^,E^21^]hGIP_(5–31)_-K^11^(γE-C16))^22^. All peptides were provided by the Novo Nordisk Research Center Indianapolis, and have been previously validated in vitro and in vivo for receptor specificity and their ability to decrease body weight in DIO mice^13–15,22,41^. All sequences of the used peptides are published elsewhere^13–15,22^. For assessment of drug effects under thermoneutrality (28 °C), 12–14-week-old male age-matched mice were acclimatized to the housing temperature 2 weeks before start of the studies. At study start, male C57BL6J WT, as well as global *Glp-1r*^−*/*−^ and *Gipr*^−*/*−^ deficient mice were continued to be housed at thermoneutrality (28 °C) and given ad libitum access to a HFD (60% fat, D12492; Research Diets) and treated with a single dose (30 mg kg^−1^) of either a control mAb or a GIPR antagonist mAb^23^ (synthesized and provided by Eli Lilly and Company).

### Plasma analysis and glucose or insulin tolerance tests

Plasma levels of glucose and insulin were measured after 6 h fasting. For assessment of glucose tolerance, glucose was administered i.p. at a dose of 1.5–2 g kg^−1^. For assessment of insulin tolerance, insulin (Humalog; Eli Lilly) was injected i.p. at a dose of 0.75–1.5 U kg^−1^. HbA1c was assessed from fresh blood using the DCA Vantage Analyzer (Siemens). For assessment of glucose-induced insulin secretion, glucose was given orally at a dose of 4 g kg^−1^ in 6 h fasted mice, followed by blood sampling at timepoints 0, 2, 5, 15 and 30 min after glucose administration. Commercially available enzyme-linked immunosorbent assays (ELISAs) were used according to the manufacturer’s instruction to measure insulin (Crystal Chem Zaandam, 90080), total GIP (Sigma-Aldrich, EZRMGIP-55K), triglycerides (Wako Chemicals, 290-63701 or Abcam, ab65336) or total cholesterol (Thermo Fisher Scientific, 10178058).

### Indirect calorimetry

Energy expenditure, food intake, respiratory exchange ratio (RER) and locomotor activity were assessed for 96–132 h, and after 24 h of acclimatization, in single-housed mice using the Promethion climate-controlled indirect calorimetric system (Sabel Systems). For assessment of acute food intake, mice were treated with either vehicle or acyl-GIP (IUB0271)^13–15^ at the indicated doses, followed by measurement of food intake for 16 h. Data for energy expenditure were analysed using analysis of covariance (ANCOVA) with body weight as a covariate^70,71^.

### RNA extraction and gene expression analysis

Total RNA was isolated using the RNeasy Kit (Qiagen) according to the manufacturer’s instructions. cDNA synthesis was performed using the QuantiTect Reverse Transcription kit (Qiagen) or the High-Capacity cDNA Reverse Transcription kit (Thermo Fisher Scientific), according to the manufacturer’s instructions. Gene expression was profiled using SYBR green (Thermo Fisher Scientific) and the Quantstudio 7 flex cycler (Applied Biosystems). The relative expression levels of each gene were normalized to the housekeeping gene peptidylprolyl isomerase A (*Ppia*), hypoxanthin-phosphoribosyl-transferase 1 (*Hprt*) or the TATA-binding protein (*Tbp*). The decision to use either *Ppia*, *Hprt* or *Tbp* was made based on the lowest variability of the housekeeper across the samples in the given tissue. Primer sequences were *Ppia*-F: 5′-GAG CTG TTT GCA GAC AAA GTT C-3′; *Ppia*-R: 5′-CCC TGG CAC ATG AAT CCT GG-3′; *Hprt*-F: 5′-AAG CTT GCT GGT GAA AAG GA-3′; *Hprt*-R: 5′-TTG CGC TCA TCT TAG GCT TT-3′; *Gipr*-F: 5′-GGC CCA GAT CAT GAC CCA AT-3′; *Gipr*-R: 5′-AGC CAA GAA GCA GGT AGC AG-3′; *Prph*-F: 5′-AAG TTT AAA GAC GAC TGT GCC TG-3′; *Prph*-R: 5′-TGC TGT TCC TTC TGG GAC TCT-3′; *Tbp*-F: 5′-GAA GCT GCG GTA CAA TTC CAG-3′; *Tbp*-R: 5′-CCC CTT GTA CCC TTC ACC AAT-3′. All raw CT values are stated in the Source data.

### RNAscope and immunostaining

For brain isolation, mice were perfused with PBS, followed by 4% paraformaldehyde (PFA). Brains were then fixed for 24 h at 4 °C in 4% PFA and then transferred to 15% sucrose for 24 h, followed by 24 h in 30% sucrose at 4 °C. For DRG, trigeminal ganglion and nodose ganglion, tissues were extracted and fixed for 1–2 h in 4% PFA and transferred to 30% sucrose overnight at 4 °C. All tissues were frozen in Tissue-Tek O.C.T (Sakura Finetek, 4583), cut in 12–14 µm sections and placed on microscopic slides (Thermo Fisher Scientific, 10149870). The slides were heated for 30 min at 60 °C followed by antigen retrieval using a steamer, then processed by the RNAscope Multiplex Fluorescent Reagent kit v2 (Advanced Cell Diagnostics, 323270) according to the manufacturer’s instructions. In brief, a custom-made probe was designed to bind to the deleted exons of mouse *Gipr* (Advanced Cell Diagnostics, 1138821-C1) and Vgat (Advanced Cell Diagnostics, 319191-C2) hybridized to the RNA, before preamplifiers, amplifiers and dyes were added for visualization of GIPR and Vgat. The slides were incubated with rabbit anti-peripherin antibody (Thermo Fisher Scientific, PA316723; 1:200) for 1 h at room temperature, followed by 30 min incubation with goat anti-rabbit-HRP (Thermo Fisher Scientific, A16096, 1:1,000) at room temperature. TSA vivid dyes 650 and 520 (Advanced Cell Diagnostics, 323271 and 323273, both 1:500 dilution) were added to detect GIPR, peripherin or vesicular GABA transporter (VGAT), respectively. Slides were counterstained with 4,6-diamidino-2-phenylindole (DAPI) (Advanced Cell Diagnostics, 320858) and imaged using Leica SP8 Laser Confocal Microscope using LAS X (version 3.5.7.23225).

### Pancreatic islets isolation

Mice were killed by cervical dislocation, followed immediately by clamping of the bile duct and perfusion with collagenase P (Roche Diagnostics, 11249002001). Tissues were incubated in a 15 ml Falcon tube with 1 ml of collagenase P solution for 15 min at 37 °C, followed by the addition of 12 ml of cold G-solution (Sigma-Aldrich) and centrifugation at 586*g* at room temperature. The pellet was subsequently washed with 10 ml of G-solution (500 ml HBSS (Life Technologies, BE10-508F) with 10% BSA (Sigma-Aldrich, 126615-25 ml) and 1% penicillin–streptomycin (Life Technologies, 15140122)) and resuspended in 5.5 ml of gradient solution comprising 15% Optiprep (5 ml 10% RPMI (Life Technologies, 11875093) + 3 ml of 40% Optiprep that was diluted from 60% Optiprep with G-solution (Sigma-Aldrich, D1556)) per sample, and placed on top of 2.5 ml of the gradient solution. To form a three-layer gradient, 6 ml of the G-solution was added on the top. Samples were then incubated for 10 min at room temperature and centrifuged at 630*g*. The interphase was then collected and filtered through a 70 μm nylon filter (BD Falcon, 352350), before washing with G-solution. Islets were handpicked by a micropipette under the microscope and cultured in RPMI 1640 medium (Life Technologies, 11875093) overnight.

### Ex vivo glucose-stimulated insulin secretion from pancreatic islets

Culture medium was removed and islet microtissues were equilibrated for 1 h with Krebs Ringer HEPES buffer (131 mM NaCl, 4.8 mM KCl, 1.3 mM CaCl_2_, 25 mM HEPES, 1.2 mM KH_2_PO_4_, 1.2 mM MgSO_4_ and 2% BSA) containing 2.8 mM glucose. The supernatant was collected as a sample under the low glucose condition for 45 min incubation, and islets were incubated for another 45 min at 37 °C with Krebs Ringer HEPES buffer containing 16.7 mM glucose and supplements as above. The supernatant was collected as a sample under the high glucose condition and stored at −20 °C. For drug-induced insulin secretion, native mouse GIP or GLP-1 (provided by Novo Nordisk) were diluted in 1× KRK buffer with 20 mM glucose to reach a concentration of 50 nM. Cells were subsequently treated with either mouse GIP or GLP-1 for 45 min. Insulin concentrations were determined using a Mouse Insulin ELISA (Crystal Chem, 90082).

### snRNA-seq

For snRNA-seq, 35-week-old DIO mice were treated 2 h before the end of the light phase with a single s.c. injection of either vehicle (PBS), acyl-GIP (150 nmol kg^−1^)^13–15^, acyl-GLP-1 (50 nmol kg^−1^)^13–15^, the GIPR:GLP-1R co-agonist MAR709 (50 nmol kg^−1^)^13–15^ or an acylated peptide GIPR antagonist (1,500 nmol kg^−1^)^22^. The hypothalamus and DVC were collected 8 h after drug administration and stored in liquid nitrogen. Mice were euthanized followed by immediate decapitation and then the skull was removed. An earlier alignment of the brain was determined using a brain matrix and the entire hypothalamus (includes all the nuclei) was collected by microdissection. The hindbrain DVC was microdissected in an area postrema-centric manner after removal of cerebellar cortex. Tissue samples were flash frozen into liquid nitrogen and frozen tissues were stored in liquid nitrogen vapour phase for further processing to single-nuclei isolation. Nuclei were isolated using the 10X Genomics Chromium Nuclei Isolation kit including RNase Inhibitor (10X Genomics, PN-1000494), and using the 10X Genomics protocol for Single Cell Multiome ATAC + Gene Expression (10X Genomics, CG000505 Rev A). Nuclei concentration was determined using a Luna-II Automated Cell Counter (Logos biosystems, L40002) and adjusted to 6,250 nuclei per microlitre after pooling of *n* = 3 mice per sample. Nuclei were then processed using the 10X Genomics Chromium Next GEM Single Cell Multiome ATAC + Gene Expression (Rev. E) according to the manufacturer’s instructions. Pooled samples were loaded into two lanes per group for a total of 24 lanes across three 10X Chromium chips. Equal numbers of cells per sample were loaded on a 10X Genomics Chromium controller instrument to generate single-cell gel beads in emulsion at the Helmholtz Munich Genomics Core Facility. Single-nucleus multimodal libraries were sequenced using the Illumina NovaSeq 6000. FASTQ files were generated from base calls with bcl2fastq software v2.20 (Illumina). Reads were mapped to the pre-built mm10 mouse reference (University of California Santa Cruz mm10 reference genome) using Cell Ranger ARC (v2.0.2, 10X Genomics) with default parameters. The resulting cell-by-peak and cell-by-gene matrices (ATAC and gene expression assays, respectively) from the 24 samples were integrated using Cellranger aggr (10X Genomics).

### Single-nucleus RNA data preprocessing, clustering and annotation

The raw gene expression matrix was filtered after removal of cells with either more than five mean absolute deviations more mitochondrial gene expression unique molecular identified counts, fewer than 500 detected genes or with more than 5 mean absolute deviations in total unique molecular identified counts. Scrublet^72^ was used to identify likely doublets, Leiden clustering was performed and clusters containing majority likely doublets were removed. After filtering, 211,537 nuclei from the hypothalamus and 57,798 nuclei from the DVC were used for further analysis. The processed gene expression matrix was imported into Scanpy (v1.9.8)^73^ and normalized using Scran^74^. The 4,000 most-variable genes were used for principal component analysis and the top 50 principal components were used for the Uniform Manifold Approximation and Projection (UMAP) visualization. We built a *k*-nearest neighbour graph for clustering using *k* = 50 nearest neighbours. Then, the Leiden clustering algorithm was used to group the cells into different clusters. To annotate hypothalamic cells, we used scArches^75^ to transfer labels from the HypoMap^48^ at the C66 cell annotation level. Then the expression of marker genes from the HypoMap was evaluated in each Leiden cluster, and then was manually annotated informed by marker gene expression and the majority cell type from scArches label transfer. The HypoMap hierarchical cell-type annotation framework was then used to map C25, C7 and C2 cell-type annotations. To annotate DVC cell types, as there is no comparable atlas and annotation framework to the hypomap for the DVC, each Leiden cluster was manually annotated into 35 fine-grained cell types (C35 cell type), and were then mapped to the coarser-grained C12 and C2 levels of cell type based on the expression of marker genes. DVC neurons were further subdivided into individual clusters labelled by major neurotransmitter expression.

### Single-nucleus RNA downstream analysis

For comparison of gene expression differences in DVC and hypothalamic neurons, the mean log fold difference in normalized expression of each gene was compared between the treatment groups and the DIO control group. Linear regression and Spearman correlation coefficients were calculated between treatment groups. Cell type prioritization was done using the Pertpy^76^ implementation of Augur^42^, which uses a random forest classifier to assess how accurately the experimental condition of cells within a given cell type can be predicted based on their gene expression profiles. The Augur score is given by the performance of the classifier, measured as the area under the receiver operating characteristic curve. Cell type prioritization comparisons are always made between the experimental group and the DIO vehicle control. Differential gene expression analysis was performed using Scanpy’s tl.rank_genes_groups function to identify genes that were differentially expressed between two experimental groups within a given cell type, genes with fewer than 30 counts were filtered for each comparison. The Wilcoxon rank-sum test was applied to assess differences in gene expression between groups. Default parameters were used, and multiple testing was accounted for by adjusted *P* values using the Benjamini–Hochberg method. Only genes with an adjusted *P* value <0.05 were considered statistically significant. Cell–cell communication between DVC and hypothalamic cells was performed using LIANA+^49^ implementation of the CellPhoneDB^50^ algorithm combined with the receptor–ligand database from NeuronChat^51^. DVC neurons were specified as sender cell types and hypothalamic neurons as receiver cell types.

### Integration and comparison with public datasets

We selected the 2,500 most variable genes in our DVC snRNA-seq data and used scVI^55^ to integrate snRNA-seq data from Ludwig et al.^54^ and Hes et al.^53^, subset to the same 2,500 genes. We used treeArches to create a manual tree with three layers of granularity in cell type in our data. We trained the scVI model on the control groups from each dataset using the study as the batch variable, then updated the model with the experimental groups. We mapped our own data with the Hes^53^ and Ludwig^54^ datasets into a joint latent space using scArches^75^, and then mapped the parameters for hierarchical progressive learning from scHPL v1.0.5 (ref. ^56^) to predict the cell type from our annotation framework each cell type from the Hes^53^ and Ludwig^54^ datasets correspond to.

### Replicates, randomization and blinding

In vivo studies were performed in male or female age-matched mice that were randomly distributed to achieve groups of equal body weight and body composition. The number of independent biological samples per group is indicated in the figure legends and Source data. No animals were excluded from the studies unless health issues demanded exclusion of single mice (for example, due to fighting injuries) as indicated in the Source data. For in vivo studies, drugs were aliquoted by a lead scientist in number-coded vials and most, but not all, handling investigators were blinded to the treatment condition. Analyses of glucose and insulin tolerance were performed by experienced research assistants who did not know prior treatment conditions.

### Statistical analysis

For animal studies, sample sizes were calculated based on a power analysis assuming that a body weight difference of ≥5 g between the treatment groups can be captured with a power of ≥75% when using a two-sided, two-tailed statistical test under the assumption of a s.d. of 3.5 and an α level of 0.05. Statistical analyses were performed using the statistical tools implemented in GraphPad Prism10 (version 10.0.3) and after testing of data for normal distribution using the Kolmogorov–Smirnov test, D’Agostino and Person test, Anderson–Darling test or Shapiro–Wilk test implemented in GraphPad Prism (version 10.0.3). Nonparametric tests such as the Mann–Whitney *U* test or the Kruskal–Wallis test were used to analyse data that were not normally distributed. Normally distributed data were analysed with the following parametric tests: two-tailed Student’s *t*-test, one-way analysis of variance (ANOVA) or two-way ANOVA with time and genotype as co-variants followed by Bonferroni’s post hoc multiple comparison test for individual timepoints. All data met the assumption of the statistical tests used. All results are given as mean ± s.e.m. *P* < 0.05 was considered statistically significant, with asterisks indicating significance at *P < 0.05, **P < 0.01 and ***P < 0.001. Differences in energy expenditure were calculated using ANCOVA with body weight as co-variate using SPSS (version 24). No data were excluded from the analysis unless for animal welfare reasons (for example, injury due to fighting) or identification of singular outlier using Grubbs test. Individual *P* values and outliers are shown in the Source data, unless *P* < 0.0001.

### Reporting summary

Further information on research design is available in the Nature Portfolio Reporting Summary linked to this article.

## Supplementary information

Supplementary Information RNAscope analysis of GIPR in the nodose ganglion of 59-week-old male chow-fed WT and Per-GIPR KO mice. Data are representative examples of *n* = 3 mice each group.

Reporting Summary

Supplementary Data 5 Original pictures for Supplementary Fig. 1.

## Source data

Source data Fig. 1 Statistical source data for Fig. 1.

Source data Fig. 2 Statistical source data for Fig. 2.

Source data Fig. 3 Statistical Source Data for Fig. 3.

Source data Fig. 4 Statistical source data for Fig. 4.

Source data Fig. 5 Statistical source data for Fig. 5.

Source data Fig. 6 Statistical source data for Fig. 6.

Source data Fig. 7 Statistical source data for Fig. 7.

Source data Fig. 8 Statistical source data for Fig. 8.

Source data Extended Data Fig. 1 Statistical source data for Extended Data Fig. 1.

Source data Extended Data Fig. 2 Statistical source data for Extended Data Fig. 2.

Source data Extended Data Fig. 3 Statistical source data for Extended Data Fig. 3.

Source data Extended Data Fig. 4 Statistical source data for Extended Data Fig. 4.

Source data Extended Data Fig. 5 Statistical source data for Extended Data Fig. 5.

Source data Extended Data Fig. 6 Statistical source data for Extended Data Fig. 6.

Source data Extended Data Fig. 7 Statistical source data for Extended Data Fig. 7.

Source data Extended Data Fig. 8 Statistical source data for Extended Data Fig. 8.

Source data Extended Data Fig. 9 Statistical source data for Extended Data Fig. 9.

Source data Extended Data Fig. 10 Statistical source data for Extended Data Fig. 10.

Source data Extended Data Figs. 1 and 2 Original pictures for Extended Data Figs. 1a,b and 2q,r.

## Data Availability

The snRNA-seq data are available in the GEO under SuperSeries accession number GSE288514. All data used for the statistical analysis are available in the data source file, along with the GraphPad Prism-derived report on the statistical analysis. The statistical report contains the mean difference between the treatment groups, the 95% confidence intervals, the significance summary and the exact p values (unless *P* < 0.0001). Source data are provided with this paper.
