# Publisher Correction: GIPR agonism and antagonism decrease body weight and food intake via different mechanisms in male mice

**DOI:** 10.1038/s42255-025-01308-8

**Published:** 2025-05-13

**Authors:** Robert M. Gutgesell, Ahmed Khalil, Arkadiusz Liskiewicz, Gandhari Maity-Kumar, Aaron Novikoff, Gerald Grandl, Daniela Liskiewicz, Callum Coupland, Ezgi Karaoglu, Seun Akindehin, Russell Castelino, Fabiola Curion, Xue Liu, Cristina Garcia-Caceres, Alberto Cebrian-Serrano, Jonathan D. Douros, Patrick J. Knerr, Brian Finan, Richard D. DiMarchi, Kyle W. Sloop, Ricardo J. Samms, Fabian J. Theis, Matthias H. Tschöp, Timo D. Müller

**Affiliations:** 1 Institute for Diabetes and Obesity, Helmholtz, Munich, Germany; 2 German Center for Diabetes Research, DZD, Neuherberg, Germany; 3 Institute of Computational Biology, Helmholtz Munich, Munich, Germany; 4 Department of Physiology, Faculty of Medical Sciences in Katowice, Medical University of Silesia, Katowice, Poland; 5 Department of Pharmacology, Experimental Therapy and Toxicology, Institute of Experimental and Clinical Pharmacology and Pharmacogenomics, Eberhard Karls University, Tübingen, Germany; 6 Department of Computational Health, Institute of Computational Biology, Helmholtz, Munich, Germany; 7 Department of Mathematics, School of Computation, Information and Technology, Technical University of Munich, Munich, Germany; 8 Medizinische Klinik und Poliklinik IV, Klinikum der Universität, Ludwig–Maximilians Universität München, Munich, Germany; 9 Indiana Biosciences Research Institute, Indianapolis, IN USA; 10 Diabetes, Obesity and Complications Therapeutic Area, Eli Lilly and Company, Indianapolis, IN USA; 11 Department of Chemistry, Indiana University Bloomington, Bloomington, IN USA; 12 TUM School of Life Sciences Weihenstephan, Technical University of Munich, Munich, Germany; 13 Helmholtz Munich, Munich, Germany; 14 Division of Metabolic Diseases, Department of Medicine, Technische Universität, Munich, Germany; 15 Walther-Straub Institute for Pharmacology and Toxicology, Ludwig–Maximilians University Munich, Munich, Germany

**Keywords:** Obesity, Type 2 diabetes

Correction to: *Nature Metabolism* 10.1038/s42255-025-01294-x, published online 29 April 2025.

In the version of the article initially published, in Fig. 2k the WT (black) line was mistakenly shifted to the right and did not align with the data points. In the bottom key in Fig. 3, “KO acyl-GLP-1 + GIPR ant.” was incorrectly listed as a full red circle and has been corrected to an open red circle. In Extended Data Fig. 3f and g, the labels “Light”, “Dark” and “Total” were missing and have now been added. These corrections have been made to the HTML and PDF versions of the article.

