# Supplementary material for: GIPR agonism and antagonism decrease body weight and food intake via different mechanisms in male mice
Source: Nat Metab. 2025 Apr 29;7(6):1282–98. doi: 10.1038/s42255-025-01294-x (PMC12198009; doi:10.1038/s42255-025-01294-x)
Supplement: Supplementary file 1 — RNAscope analysis of GIPR in the nodose ganglion of 59-week-old male chow-fed WT and Per-GIPR KO mice. Data are representative examples of n = 3 mice each group. [file 42255_2025_1294_MOESM1_ESM.pdf]

nature metabolism

Supplementary information https://doi.org/10.1038/s42255-025-01294-x

GIPR agonism and antagonism decrease body weight and food intake via different mechanisms in male mice

In the format provided by the authors and unedited

a

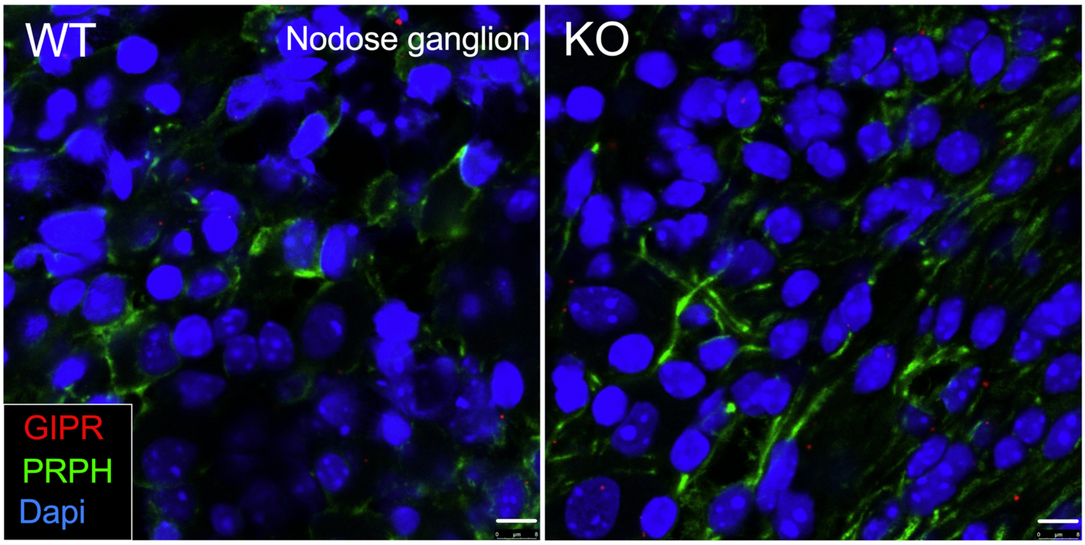

Supplementary Figure 1. Analysis of GIPR expression in the nodose ganglion of Per GIPR KO mice. RNAscope analysis of GIPR in 59-week old male chow-fed WT and Per GIPR KO mice **(a).** Data in panel **a** are representative examples of n=3 mice each group.
