# Supplementary material for: GIPR agonism and antagonism decrease body weight and food intake via different mechanisms in male mice
Source: Nat Metab. 2025 Apr 29;7(6):1282–98. doi: 10.1038/s42255-025-01294-x (PMC12198009; doi:10.1038/s42255-025-01294-x)
Supplement: Supplementary file 2 — Reporting Summary [file 42255_2025_1294_MOESM2_ESM.pdf]

nature portfolio

Corresponding author(s): Timo D. Müller

Last updated by author(s): Mar 12, 2025

Reporting Summary

Nature Portfolio wishes to improve the reproducibility of the work that we publish. This form provides structure for consistency and transparency in reporting. For further information on Nature Portfolio policies, see our Editorial Policies and the Editorial Policy Checklist.

Statistics

For all statistical analyses, confirm that the following items are present in the figure legend, table legend, main text, or Methods section.

n/a	Confirmed	
☐	☒	The exact sample size (n) for each experimental group/condition, given as a discrete number and unit of measurement
☐	☒	A statement on whether measurements were taken from distinct samples or whether the same sample was measured repeatedly
☐	☒	The statistical test(s) used AND whether they are one- or two-sided<br>*Only common tests should be described solely by name; describe more complex techniques in the Methods section.*
☐	☒	A description of all covariates tested
☐	☒	A description of any assumptions or corrections, such as tests of normality and adjustment for multiple comparisons
☐	☒	A full description of the statistical parameters including central tendency (e.g. means) or other basic estimates (e.g. regression coefficient) AND variation (e.g. standard deviation) or associated estimates of uncertainty (e.g. confidence intervals)
☐	☒	For null hypothesis testing, the test statistic (e.g. F, t, r) with confidence intervals, effect sizes, degrees of freedom and P value noted<br>*Give P values as exact values whenever suitable.*
☒	☐	For Bayesian analysis, information on the choice of priors and Markov chain Monte Carlo settings
☒	☐	For hierarchical and complex designs, identification of the appropriate level for tests and full reporting of outcomes
☐	☒	Estimates of effect sizes (e.g. Cohen's d, Pearson's r), indicating how they were calculated

Our web collection on statistics for biologists contains articles on many of the points above.

Software and code

Policy information about availability of computer code

Data collection	Immunofluorescence was imaged using LAS X (version 3.5.7.23225, Leica Microsystems CMS GmbH)
Data analysis	Statistical analyses were performed using the statistical tools implemented in GraphPad Prism10 (version 10.0.3). Differences in energy expenditure were calculated using ANCOVA with body weight as co-variate using SPSS (version 24). Analysis of snRNAseq data were performed using Augur (version 26.0.0), CellPhoneDB (version 5.0.0), scArches (version 0.6.1), Scrublet (v.02), Scanpy (v1.9.8) and Scran (v.3.20) after processing the files using bcl2fastq software v2.20 (Illumina, San Diego, USA) and Cell Ranger ARC (v2.0.2, 10X Genomics; Pleasanton, CA, USA). snRNAseq downstream analysis were performed using Augur (version 26.0.0), CellPhoneDB (v. 2.0), NeuronChat (RRID:SCR_018020) and scHPL (v1.0.5)

For manuscripts utilizing custom algorithms or software that are central to the research but not yet described in published literature, software must be made available to editors and reviewers. We strongly encourage code deposition in a community repository (e.g. GitHub). See the Nature Portfolio guidelines for submitting code & software for further information.

Data

Policy information about availability of data

All manuscripts must include a data availability statement. This statement should provide the following information, where applicable:

- Accession codes, unique identifiers, or web links for publicly available datasets
- A description of any restrictions on data availability
- For clinical datasets or third party data, please ensure that the statement adheres to our policy

The snRNAseq data are available in the GEO under SuperSeries accession number GSE288514. All data used for the statistical analysis are available in the data

source file, along with the GraphPad Prism-derived report on the statistical analysis. The statistical report contains the mean difference between the treatment groups, the 95% confidence intervals, the significance summary, and the exact p-values (unless p<0.0001).

Field-specific reporting

Please select the one below that is the best fit for your research. If you are not sure, read the appropriate sections before making your selection.

☒ Life sciences ☐ Behavioural & social sciences ☐ Ecological, evolutionary & environmental sciences

For a reference copy of the document with all sections, see nature.com/documents/nr-reporting-summary-flat.pdf

Life sciences study design

All studies must disclose on these points even when the disclosure is negative.

Sample size	For animal studies, sample sizes were calculated based on a power analysis assuming that a greater or equal (>/=) 5 g difference in body weight between genotypes can be assessed with a power of >/= 75% when using a 2-sided statistical test under the assumption of a standard deviation of 3.5 and an alpha level of 0.05.
Data exclusions	No data were excluded from the analysis unless scientific (e.g. significant outlier identified by the Grubbs test for outlier) or animal welfare reasons (e.g. injury due to fighting) demanded exclusion. Outliers are stated in the data source file.
Replication	In vivo and ex vivo data were obtained in independent biological replicates as indicated in the figure legends.
Randomization	Animals were either randomly assigned into treatment groups, or were grouped based on their genotype (WT or KO). At study start, only age-matched mice were included in the studies. There were no other covariats controlled.
Blinding	For in vivo studies, drugs were aliquoted by a lead scientist in number-coded vials and most, but not all, handling investigators were blinded to the treatment condition. Analyses of glucose and insulin tolerance were performed by experienced research assistants who did not know prior treatment conditions. Ex vivo studies were performed in ID coded vials without statement of treatment on the vials. Ex vivo studies were performed in ID coded vials, and with with most, but not all investigators, being blinded to the underlying genotypes and treatment conditions.

Reporting for specific materials, systems and methods

We require information from authors about some types of materials, experimental systems and methods used in many studies. Here, indicate whether each material, system or method listed is relevant to your study. If you are not sure if a list item applies to your research, read the appropriate section before selecting a response.

Materials & experimental systems

n/a	Involved in the study	
☐	☒	Antibodies
☒	☐	Eukaryotic cell lines
☒	☐	Palaeontology and archaeology
☐	☒	Animals and other organisms
☒	☐	Human research participants
☒	☐	Clinical data
☒	☐	Dual use research of concern

Methods

n/a	Involved in the study	
☒	☐	ChIP-seq
☒	☐	Flow cytometry
☒	☐	MRI-based neuroimaging

Antibodies

Antibodies used	Rabbit anti-peripherin antibody (Thermo Fisher Scientific, Erlangen, Germany; #PA316723; 1:200)<br>Goat anti-rabbit-HRP (Thermo Fisher Scientific Erlangen, Germany; #A16096, 1:1000)
Validation	Rabbit anti-peripherin antibody (Thermo Fisher Scientific, Erlangen, Germany; #PA316723; 1:200) is a polyclonal antibody with validated reactivity in bovine, human, mammal, mouse, pig and rat tissues. The antibody is certified to work in applications such as immunhistochemistry, immuncytochemistry and western blot. Immunocytochemistry analysis of Peripherin was demonstrated in rat pheochromocytoma PC12 cell line. Samples were incubated in Peripherin polyclonal antibody (Product # PA3-16723) using a dilution of 1:2000. Periphren antibody (Green). Hoechst staining of nuclear DNA (Blue). Peripherin, one of the Class III family of intermediate filament subunit proteins, is a major component of the PC12 cell forming a perinuclear cap, with some filaments visible in the cytoplasm. Western blot validation of Peripherin was performed in tissue and cell lysates using Peripherin . Samples were incubated in Peripherin polyclonal antibody (Product # PA3-16723 using a dilution of 1:10000. Antibody in green: [1] protein standard, [2] rat spinal cord, [3] mouse spinal cord, [4] pig spinal cord, [5] cow spinal cord, [6] SH-SY5Y cells and [7] PC12 cells. The major band at ~57 kDa corresponds to the major peripherin protein isoform, while other bands presumably represent protein products of alternate transcripts of the peripherin gene.

Goat anti-rabbit-HRP (Thermo Fisher Scientific Erlangen, Germany; #A16096, 1:1000) is a polyclonal 2nd antibody suitable for western blot, immunhistochemistry and ELISA. Western blot validation was performed on whole cell extracts (30 µg lysate) of HeLa (Lane 1) and K-562 (Lane 2). The blots were probed with Anti-PRDX6 Recombinant Rabbit Monoclonal Antibody (Product # 702211, 2 µg/mL) and detected by chemiluminescence using Goat anti-Rabbit IgG (H+L) Secondary Antibody, HRP Conjugate (Product # A16096) at dilutions 1:2,000 (Fig. 1), 1:5,000 (Fig. 2) and 1:10,000 (Fig. 3). A 25 kDa band corresponding to PRDX6 was observed. Known quantity of protein samples were electrophoresed using Novex® NuPAGE®12 % Bis-Tris gel (Product # NP0342BOX), XCell SureLock Electrophoresis System (Product # EI0002) and Novex® Sharp Pre-Stained Protein Standard (Product # LC5800). Resolved proteins were then transferred onto a nitrocellulose membrane with iBlot® 2 Dry Blotting System (Product # IB21001). The membrane was probed with the relevant primary and secondary antibody after blocking with 5 % skimmed milk. Chemiluminescent detection was performed using Pierce™ ECL Western blotting Substrate (Product # 32106).

Animals and other organisms

Policy information about studies involving animals; ARRIVE guidelines recommended for reporting animal research

Laboratory animals	Figure 1A-C: 33-wk old male HFD-fed C57BL/6J Vgat Cre+/- Gipr wt/wt (WT) and Vgat Cre+/- Gipr flx/flx (KO) mice<br>Figure 1D-M: 36-wk old male HFD-fed C57BL/6J Vgat Cre+/- Gipr wt/wt (WT) and Vgat Cre+/- Gipr flx/flx (KO) mice<br>Figure 2A-D: 14-47-wk old male chow-fed C57BL/6J Per Cre+/- Gipr wt/wt (WT) and Per Cre+/- Gipr flx/flx (KO) mice<br>Figure 2E-G: 49-wk old male chow-fed C57BL/6J Per Cre+/- Gipr wt/wt (WT) and Per Cre+/- Gipr flx/flx (KO) mice<br>Figure 2H and I: 47-wk old male chow-fed C57BL/6J Per Cre+/- Gipr wt/wt (WT) and Per Cre+/- Gipr flx/flx (KO) mice<br>Figure 2J: 48-wk old male chow-fed C57BL/6J Per Cre+/- Gipr wt/wt (WT) and Per Cre+/- Gipr flx/flx (KO) mice<br>Figure 2K-O: 51-wk old male chow-fed C57BL/6J Per Cre+/- Gipr wt/wt (WT) and Per Cre+/- Gipr flx/flx (KO) mice<br>Figure 2P: 52-wk old male chow-fed C57BL/6J Per Cre+/- Gipr wt/wt (WT) and Per Cre+/- Gipr flx/flx (KO) mice<br>Figure 3A: 49-wk old male HFD-fed C57BL/6J Per Cre+/- Gipr wt/wt (WT) and Per Cre+/- Gipr flx/flx (KO) mice<br>Figure 3B-M: 47-wk old male HFD-fed C57BL/6J Per Cre+/- Gipr wt/wt (WT) and Per Cre+/- Gipr flx/flx (KO) mice<br>Figure 4A and B: 14-16-wk old male HFD-fed C57BL/6J wildtype mice<br>Figure 4C and D: 14-16-wk old male HFD-fed C57BL/6J global germline Gipr KO mice<br>Figure 4E and F: 14-16-wk old male HFD-fed C57BL/6J global germline Glp-1r KO mice<br>Figure 5A-H: 36-wk old male HFD-fed C57BL/6J wildtype mice<br>Figure 6A-K: 36-wk old male HFD-fed C57BL/6J wildtype mice<br>Figure 7A-E: 36-wk old male HFD-fed C57BL/6J wildtype mice<br>Figure 8A-H: 36-wk old male HFD-fed C57BL/6J wildtype mice<br>Extended Data Figure 1A,B: 20-wk old male chow-fed c57BL/6J Vgat Cre+/- Gipr wt/wt (WT) and Vgat Cre+/- Gipr flx/flx (KO) mice<br>Extended Data Figure 1C-E: 33-wk old male HFD-fed C57BL/6J Vgat Cre+/- Gipr wt/wt (WT) and Vgat Cre+/- Gipr flx/flx (KO) mice<br>Extended Data Figure 1F-H: 36-wk old male HFD-fed C57BL/6J Vgat Cre+/- Gipr wt/wt (WT) and Vgat Cre+/- Gipr flx/flx (KO) mice<br>Extended Data Figure 2A: 15-wk old male chow-fed C57BL/6J Per Cre+/- Gipr wt/wt (WT) mice and 45-wk old male C57BL/6J Per Cre +/- Gipr wt/wt (WT)<br>Extended Data Figure 2B: 12-wk old male chow-fed C57BL/6J Per Cre+/- Gipr wt/wt (WT)<br>Extended Data Figure 2C-G: 45-wk old male chow-fed C57BL/6J Per Cre+/- Gipr wt/wt (WT) and Per Cre+/- Gipr flx/flx (KO) mice<br>Extended Data Figure 2H: 44-wk old male chow-fed C57BL/6J Per Cre+/- Gipr wt/wt (WT) and Per Cre+/- Gipr flx/flx (KO) mice<br>Extended Data Figure 2I-P: 12-wk old male chow-fed C57BL/6J Per Cre+/- Gipr wt/wt (WT) and Per Cre+/- Gipr flx/flx (KO) mice<br>Extended Data Figure 2Q,R: 51-wk old male chow-fed C57BL/6J Per Cre+/- Gipr wt/wt (WT) and Per Cre+/- Gipr flx/flx (KO) mice<br>Extended Data Figure 3A: 15-46-wk old female HFD-fed C57BL/6J Per Cre+/- Gipr wt/wt (WT) and Per Cre+/- Gipr flx/flx (KO) mice<br>Extended Data Figure 3B and C: 35-wk old female HFD-fed C57BL/6J Per Cre+/- Gipr wt/wt (WT) and Per Cre+/- Gipr flx/flx (KO) mice<br>Extended Data Figure 3D-G: 52-wk old female HFD-fed C57BL/6J Per Cre+/- Gipr wt/wt (WT) and Per Cre+/- Gipr flx/flx (KO) mice<br>Extended Data Figure 3H and I: 48-wk old female HFD-fed C57BL/6J Per Cre+/- Gipr wt/wt (WT) and Per Cre+/- Gipr flx/flx (KO) mice<br>Extended Data Figure 3J and K: 54-wk old female HFD-fed C57BL/6J Per Cre+/- Gipr wt/wt (WT) and Per Cre+/- Gipr flx/flx (KO) mice<br>Extended Data Figure 3L: 50-wk old female HFD-fed C57BL/6J Per Cre+/- Gipr wt/wt (WT) and Per Cre+/- Gipr flx/flx (KO) mice<br>Extended Data Figure 3M and N: 54-wk old female HFD-fed C57BL/6J Per Cre+/- Gipr wt/wt (WT) and Per Cre+/- Gipr flx/flx (KO) mice<br>Extended Data Figure 3O and P: 55-wk old female HFD-fed C57BL/6J Per Cre+/- Gipr wt/wt (WT) and Per Cre+/- Gipr flx/flx (KO) mice<br>Extended Data Figure 4A-C: 47-wk old male HFD-fed C57BL/6J Per Cre+/- Gipr wt/wt (WT) and Per Cre+/- Gipr flx/flx (KO) mice<br>Extended Data Figure 5A-I: 36-wk old male HFD-fed C57BL/6J wildtype mice<br>Extended Data Figure 6A-H: 36-wk old male HFD-fed C57BL/6J wildtype mice<br>Extended Data Figure 7A-I: 36-wk old male HFD-fed C57BL/6J wildtype mice<br>Extended Data Figure 8A-D: 36-wk old male HFD-fed C57BL/6J wildtype mice<br>Extended Data Figure 9A: 36-wk old male HFD-fed C57BL/6J wildtype mice<br>Extended Data Figure 10A: 36-wk old male HFD-fed C57BL/6J wildtype mice<br>Supplementary Figure 1A: 59-wk old male chow-fed C57BL/6J Per Cre+/- Gipr wt/wt (WT) and Per Cre+/- Gipr flx/flx (KO) mice
Wild animals	no wild animals were used in the study
Field-collected samples	no field collected animals were used in the study
Ethics oversight	Experiments were performed in accordance with the Animal Protection Law of the European Union after permission by the Government of Upper Bavaria, Germany, or the Eli Lilly and Company Institutional Animal Care and Use Committee, Indianapolis, IN, USA.

Note that full information on the approval of the study protocol must also be provided in the manuscript.
