# Supplementary figures and images for: GIPR agonism and antagonism decrease body weight and food intake via different mechanisms in male mice
Source: Nat Metab. 2025 Apr 29;7(6):1282–98. doi: 10.1038/s42255-025-01294-x (PMC12198009; doi:10.1038/s42255-025-01294-x)

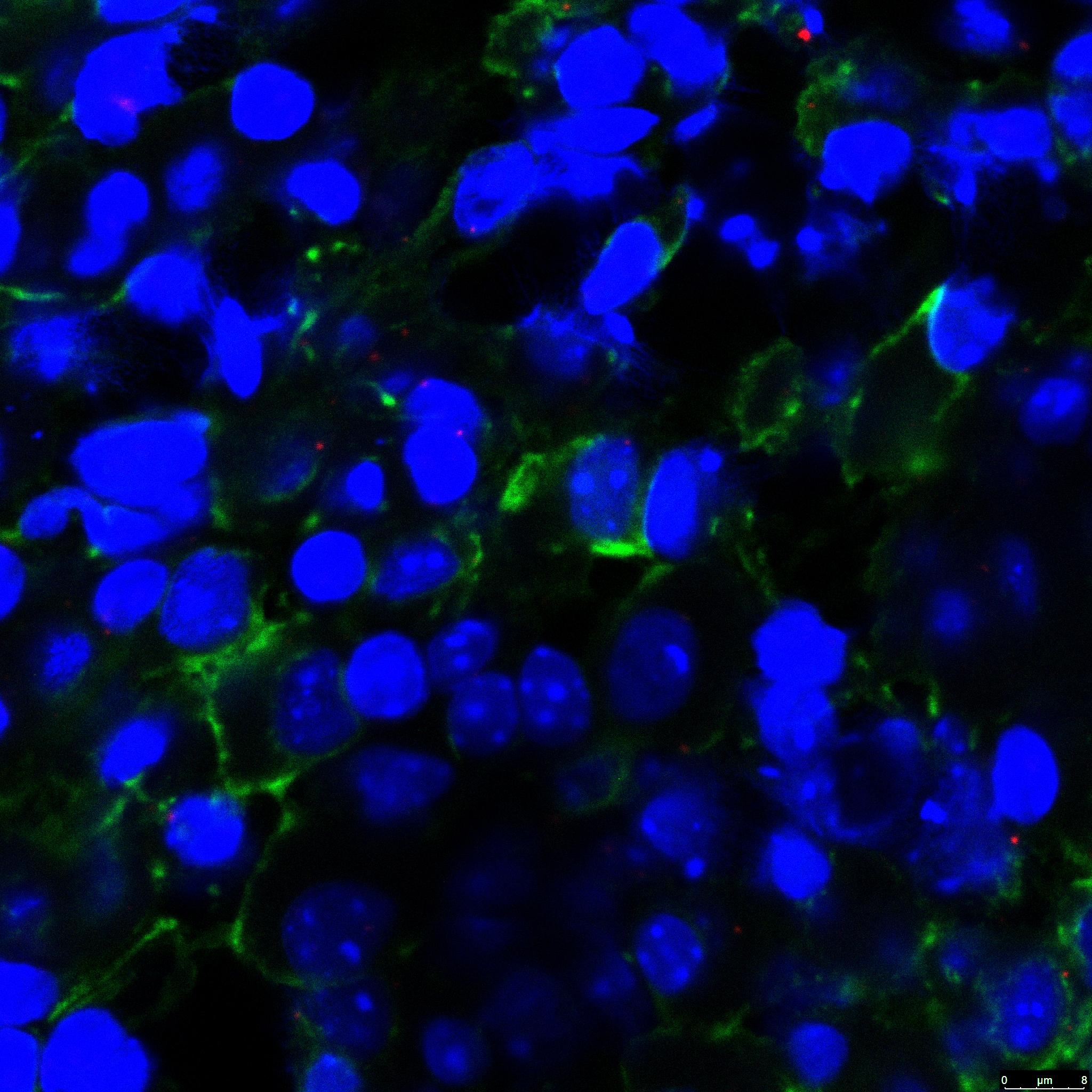

Supplement: Supplementary file 3 — Original pictures for Supplementary Fig. 1. [file 42255_2025_1294_MOESM3_ESM.zip › Original pictures Suppl Fig 1/Nodose ganglion_WT1 NG 63x.jpg]

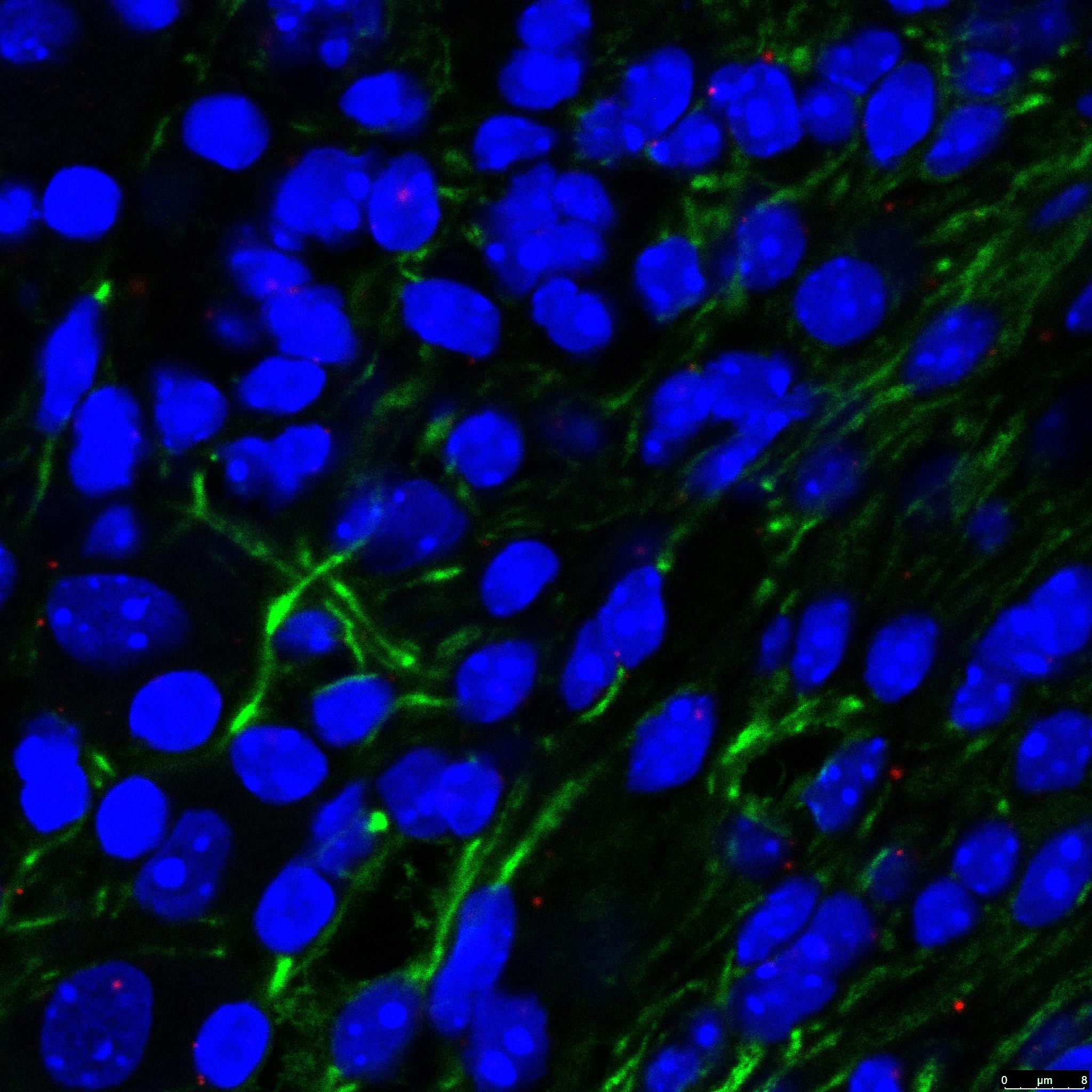

Supplement: Supplementary file 3 — Original pictures for Supplementary Fig. 1. [file 42255_2025_1294_MOESM3_ESM.zip › Original pictures Suppl Fig 1/Nodose ganglion_KO2 MG 63X.jpg]

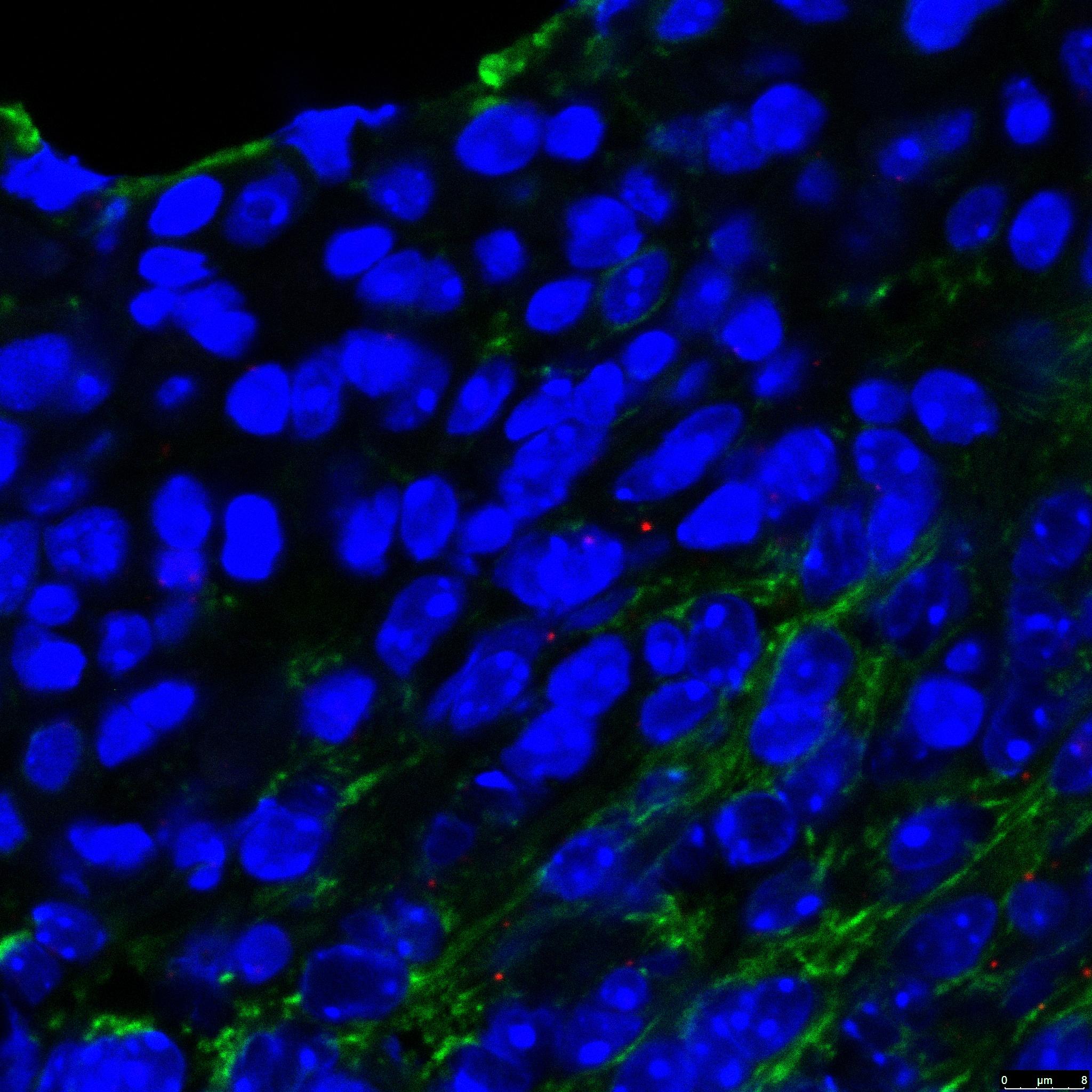

Supplement: Supplementary file 3 — Original pictures for Supplementary Fig. 1. [file 42255_2025_1294_MOESM3_ESM.zip › Original pictures Suppl Fig 1/Nodose ganglion_KO3 NG 63X.jpg]

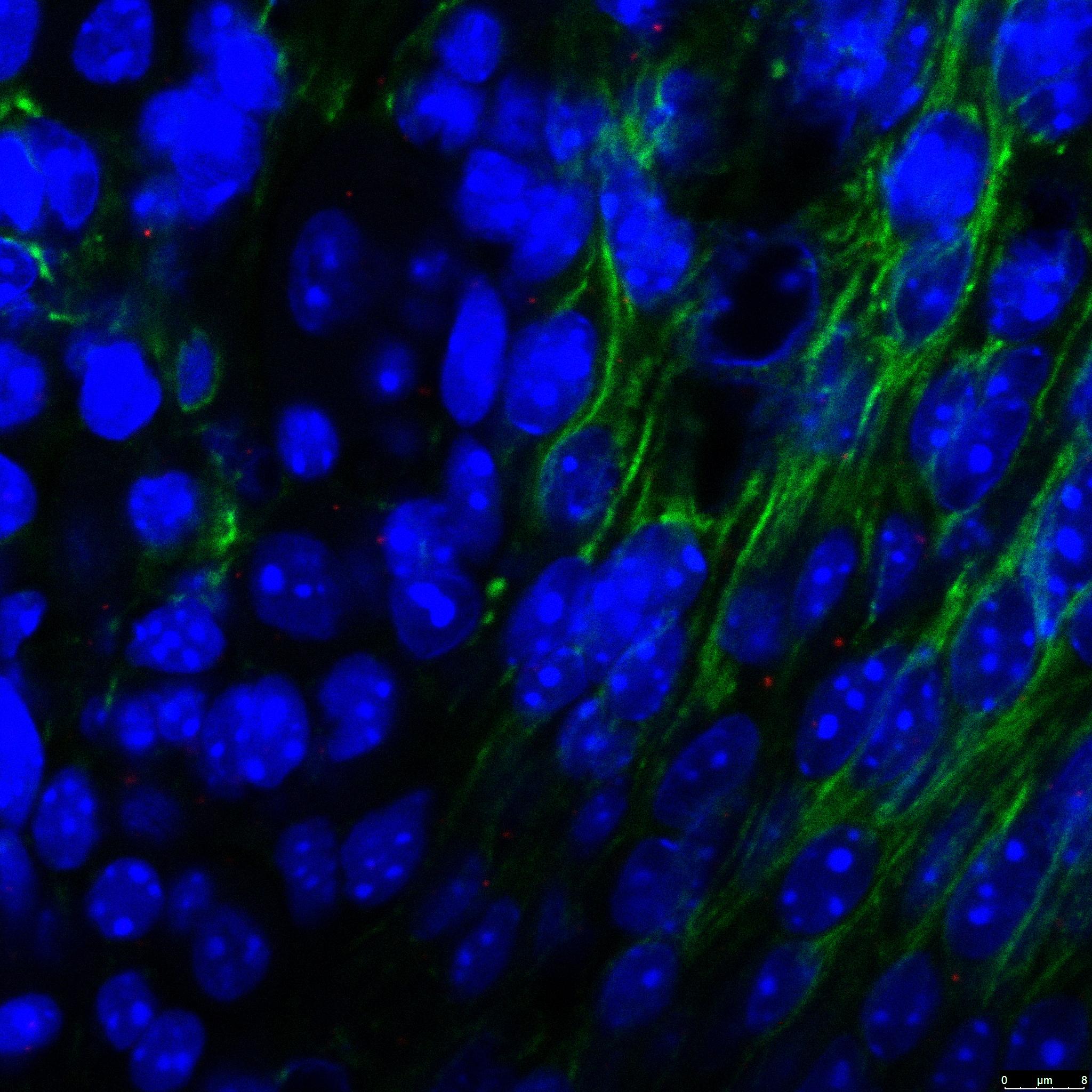

Supplement: Supplementary file 3 — Original pictures for Supplementary Fig. 1. [file 42255_2025_1294_MOESM3_ESM.zip › Original pictures Suppl Fig 1/Nodose ganglion_WT3 NG 63X.jpg]

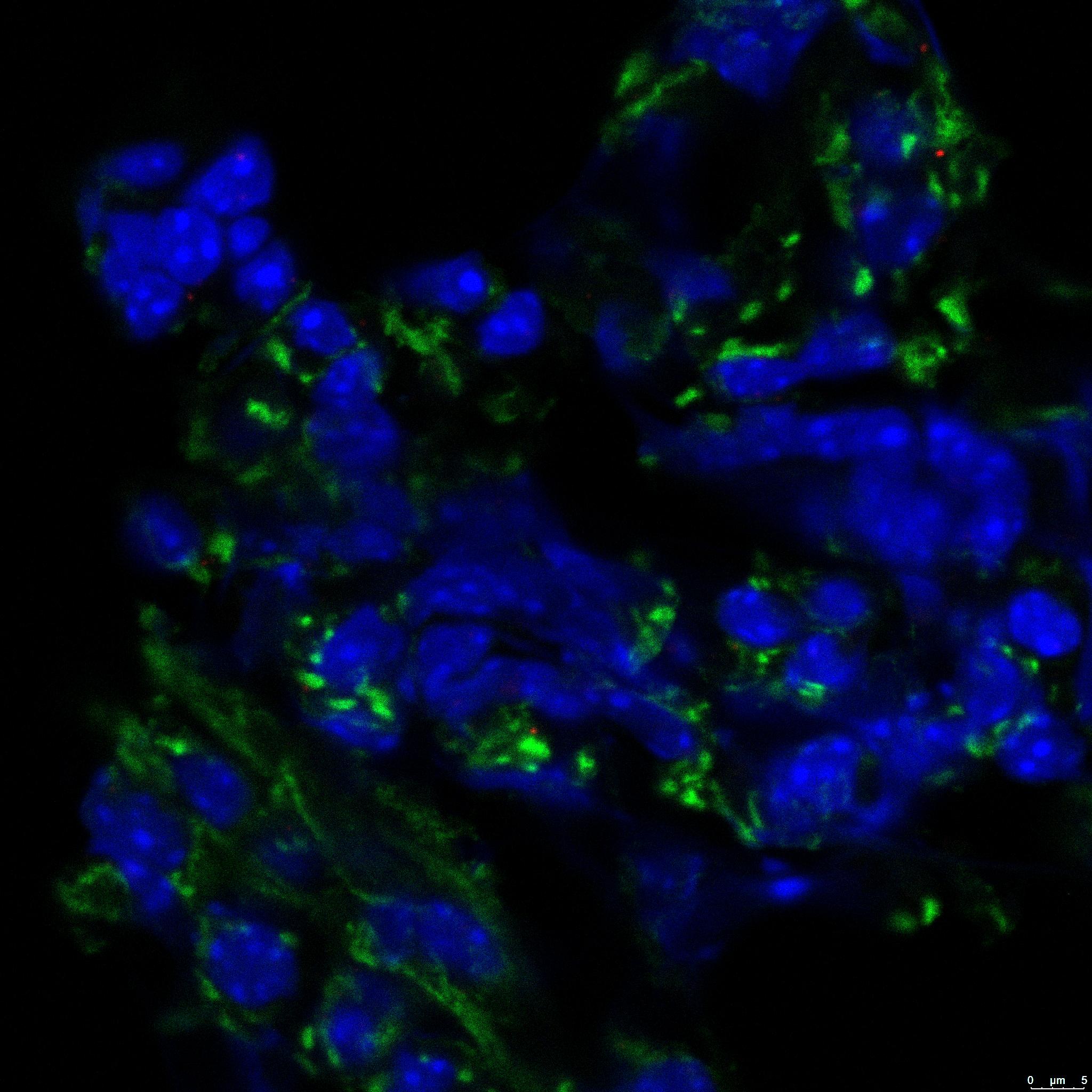

Supplement: Supplementary file 3 — Original pictures for Supplementary Fig. 1. [file 42255_2025_1294_MOESM3_ESM.zip › Original pictures Suppl Fig 1/Nodose ganglion_KO4 NG 63X.jpg]

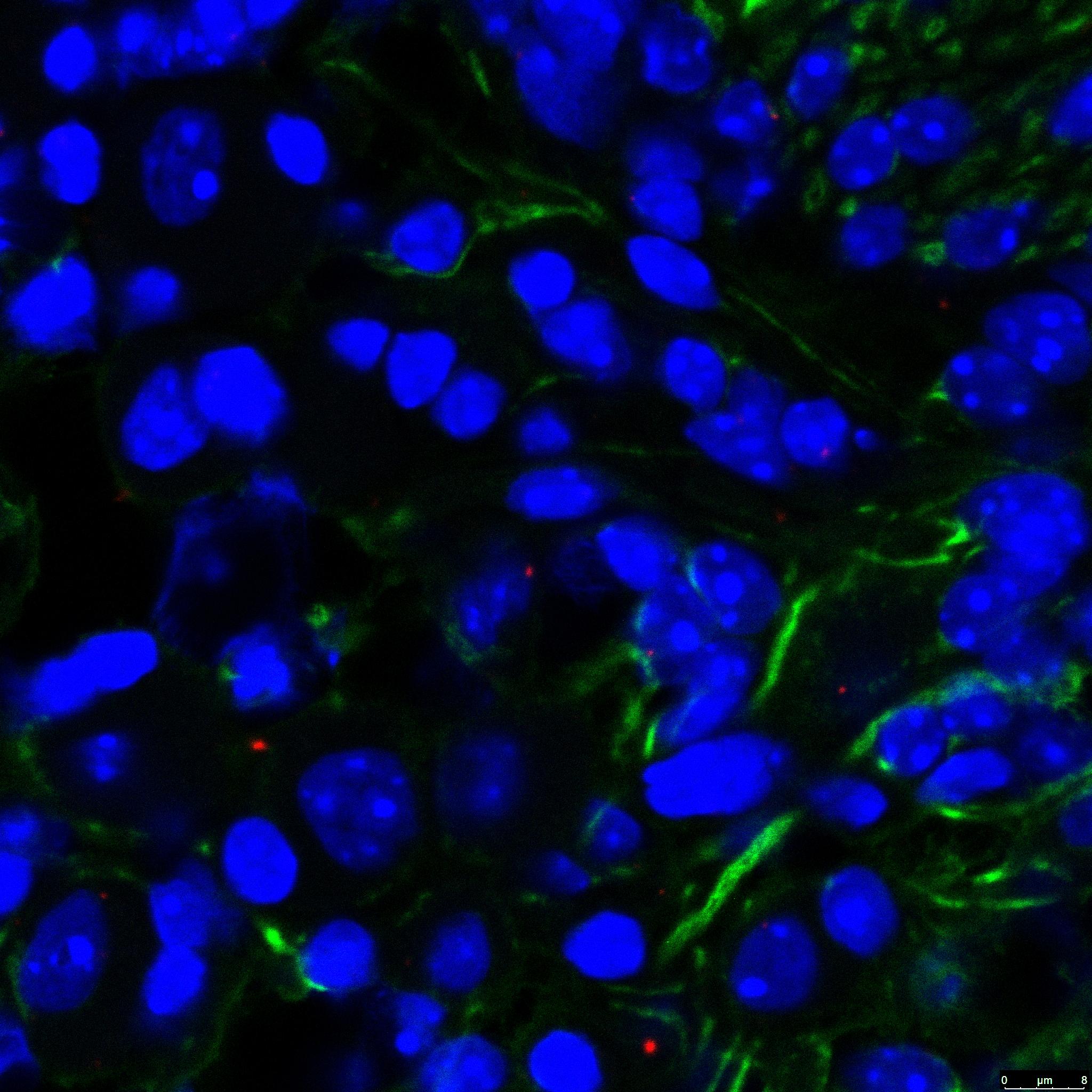

Supplement: Supplementary file 3 — Original pictures for Supplementary Fig. 1. [file 42255_2025_1294_MOESM3_ESM.zip › Original pictures Suppl Fig 1/Nodose ganglion_WT2 NG 63X.jpg]

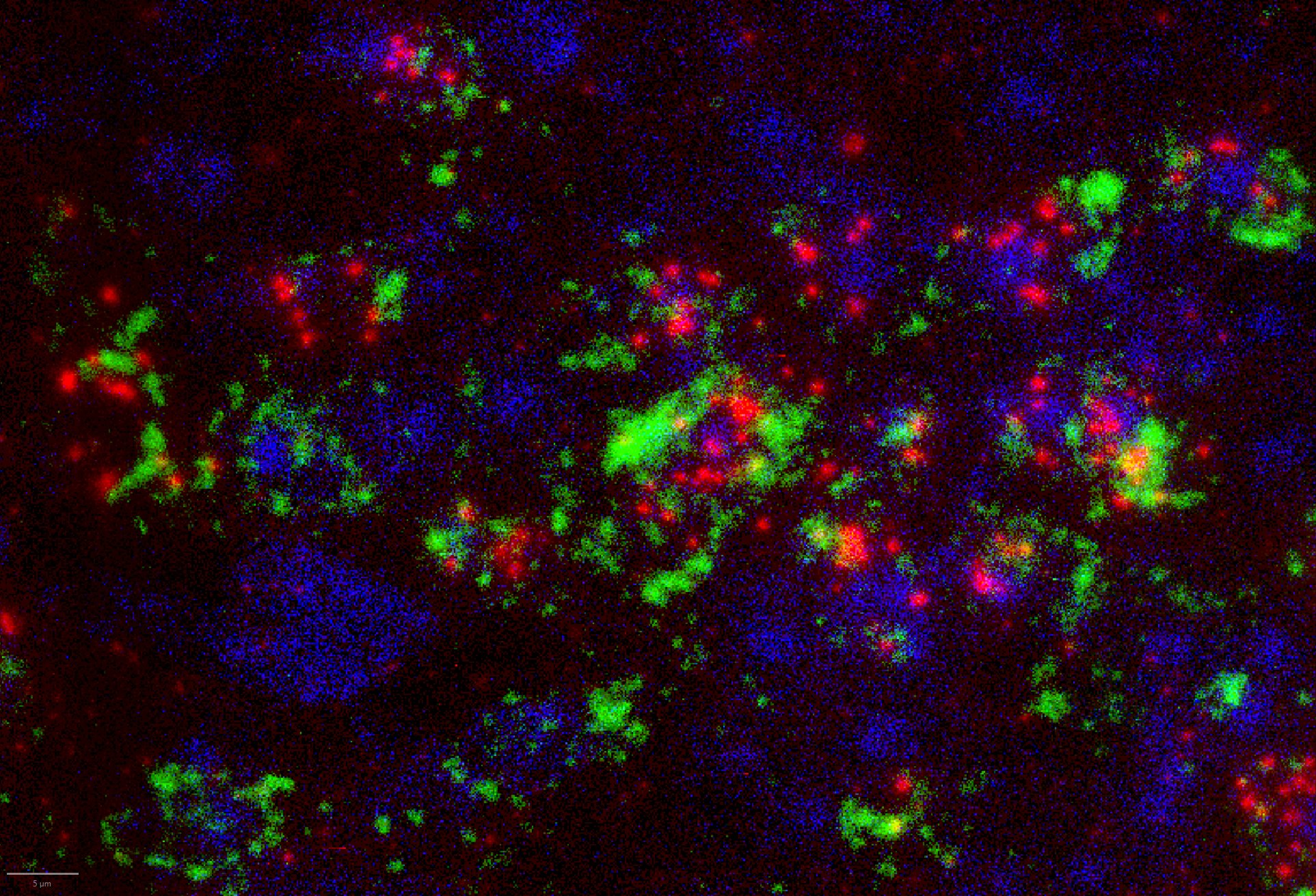

Supplement: Supplementary file 22 — Original pictures for Extended Data Figs. 1a,b and 2q,r. [file 42255_2025_1294_MOESM22_ESM.zip › Original pictures EDF1a/WT-3_Vgat_Gipr_RNAscope_zoom.jpg]

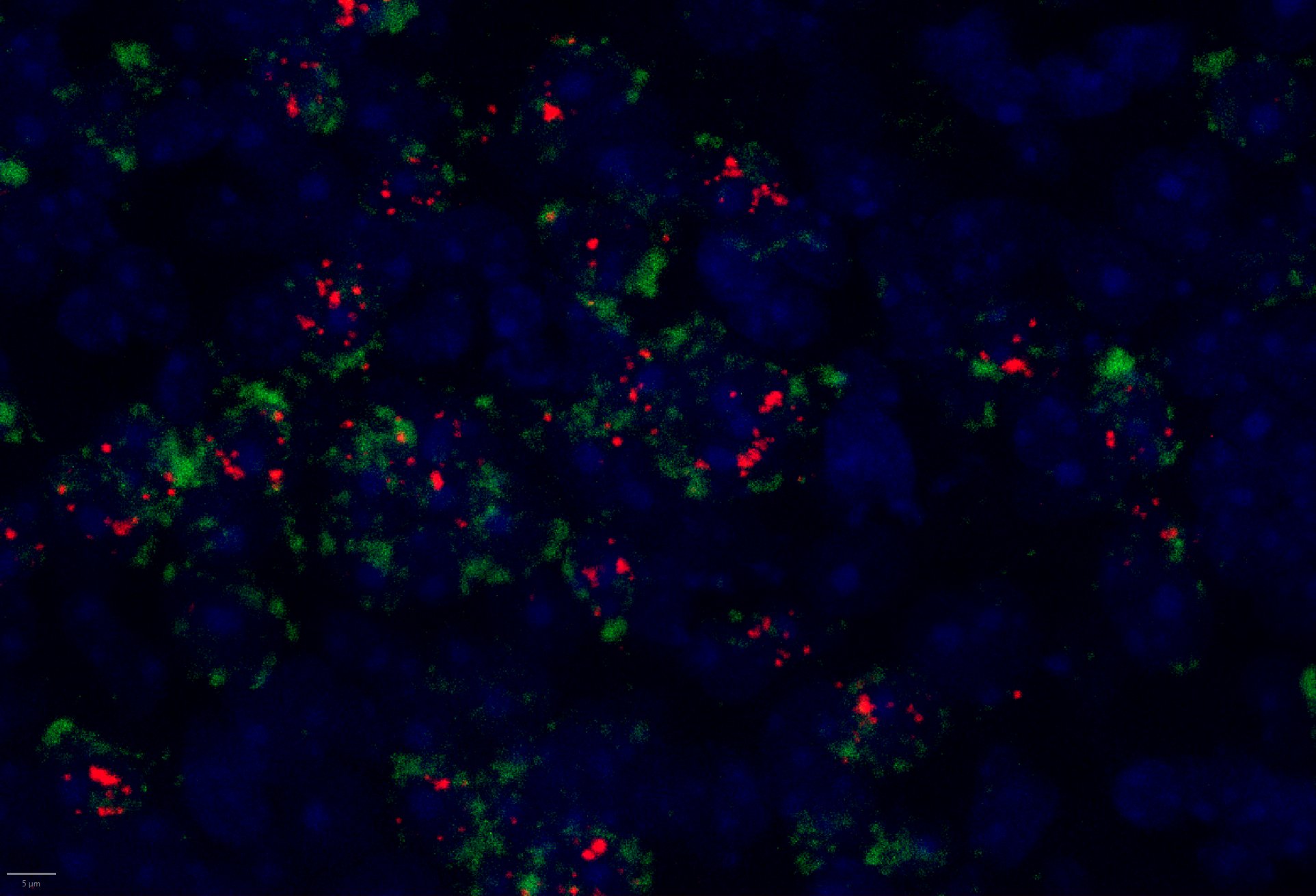

Supplement: Supplementary file 22 — Original pictures for Extended Data Figs. 1a,b and 2q,r. [file 42255_2025_1294_MOESM22_ESM.zip › Original pictures EDF1a/WT-1_Vgat_Gipr_RNAscope_zoom_used in paper.jpg]

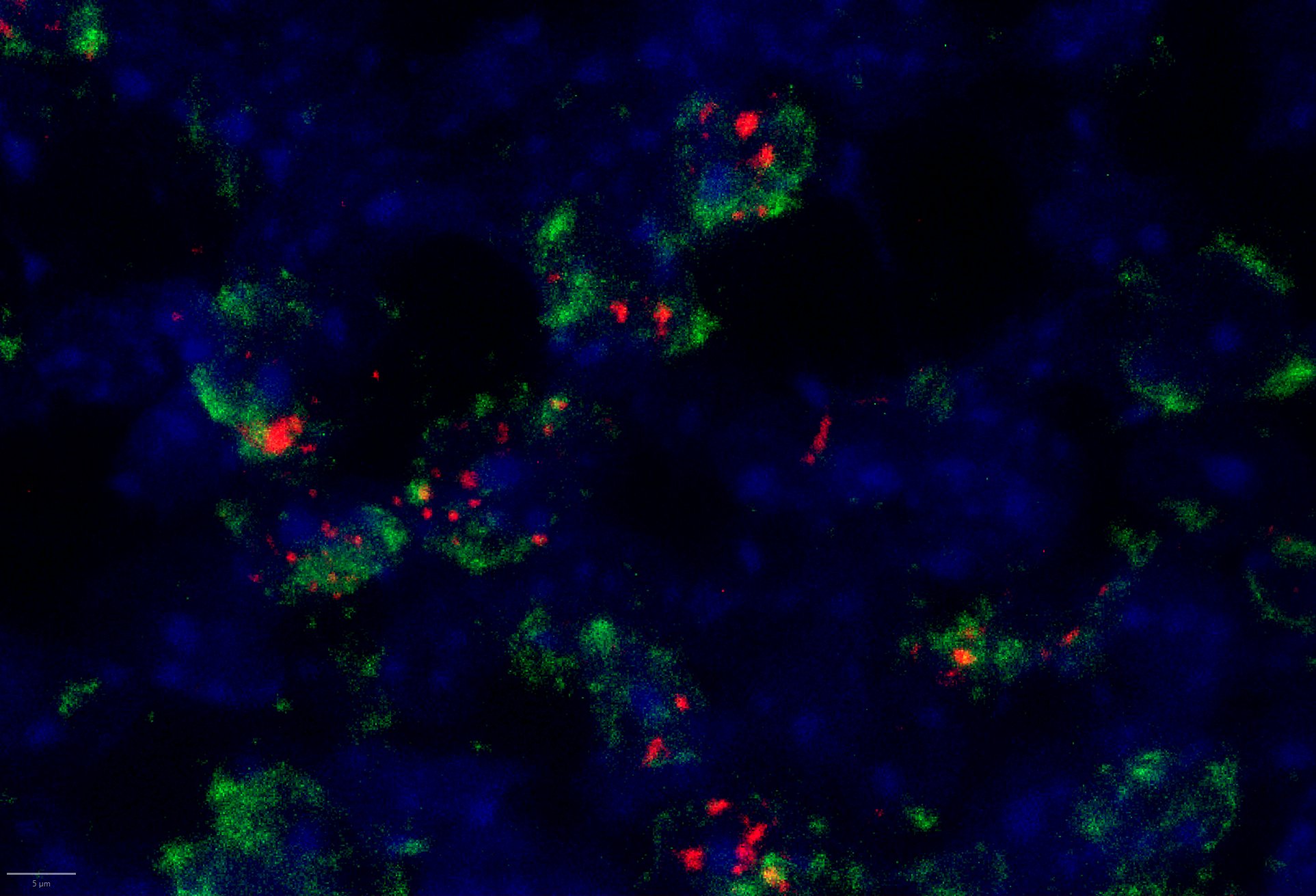

Supplement: Supplementary file 22 — Original pictures for Extended Data Figs. 1a,b and 2q,r. [file 42255_2025_1294_MOESM22_ESM.zip › Original pictures EDF1a/WT-2_Vgat_Gipr_RNAscope_zoom.jpg]

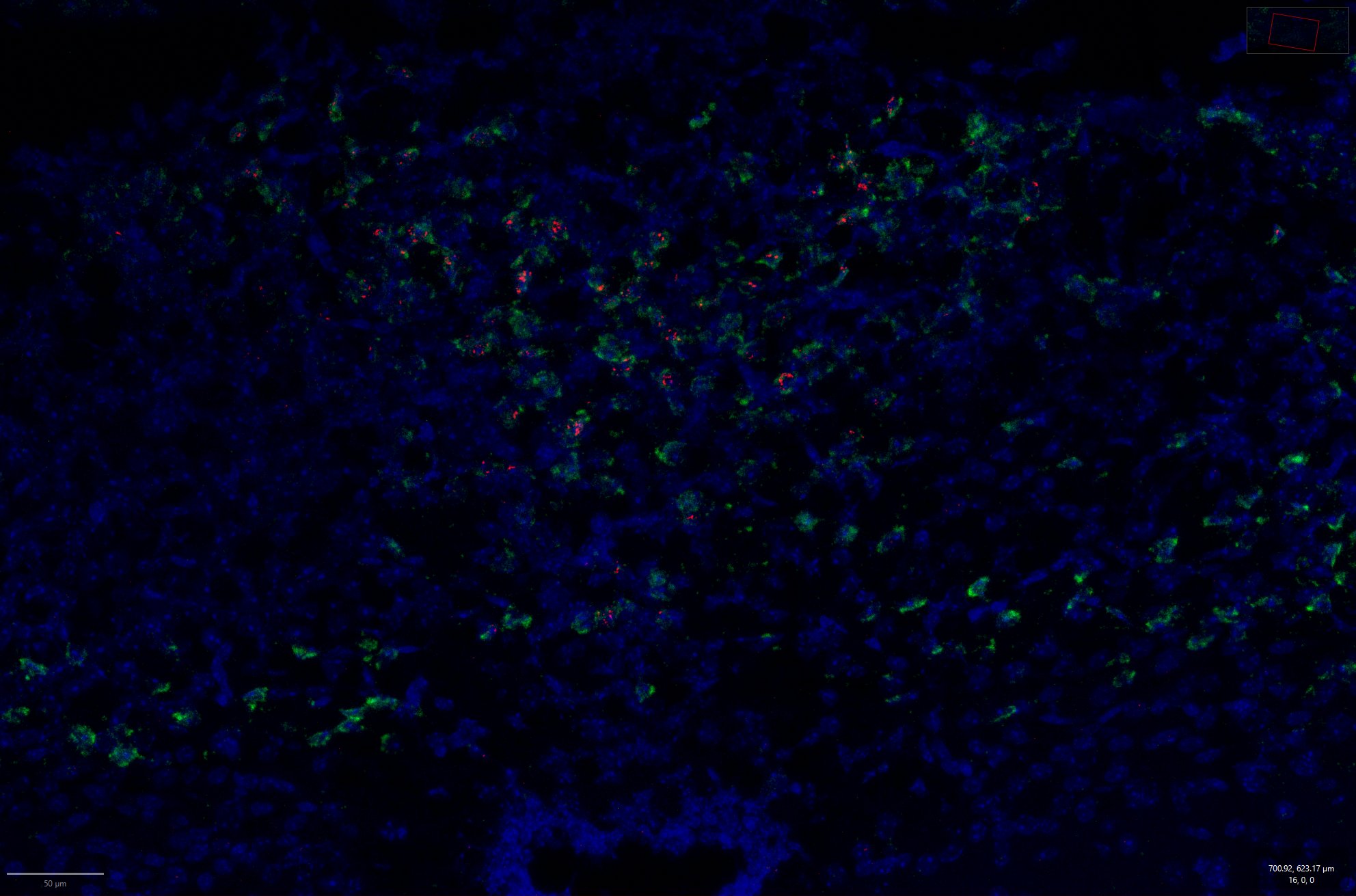

Supplement: Supplementary file 22 — Original pictures for Extended Data Figs. 1a,b and 2q,r. [file 42255_2025_1294_MOESM22_ESM.zip › Original pictures EDF1a/WT-2_Vgat_Gipr_RNAscope.jpg]

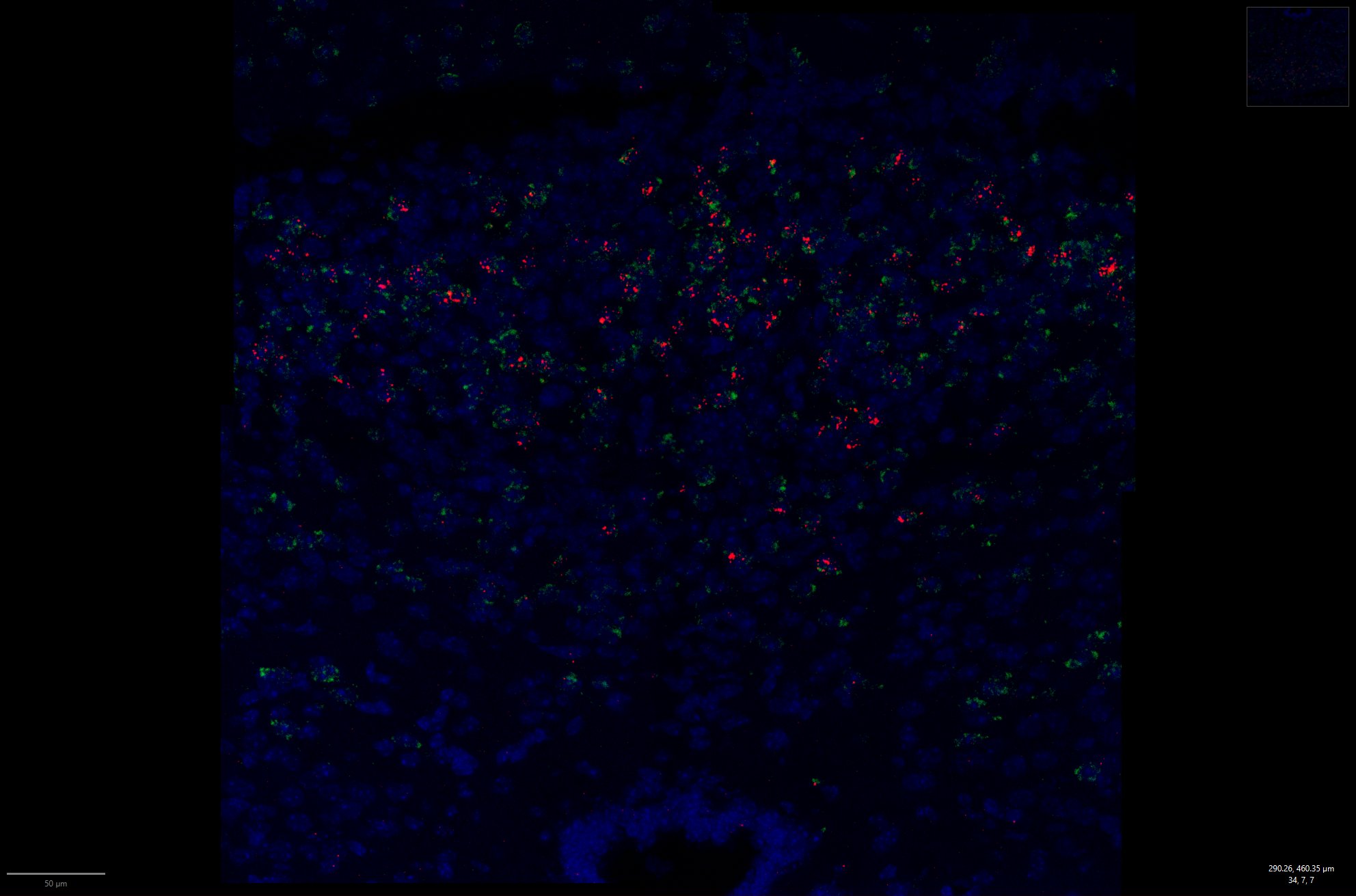

Supplement: Supplementary file 22 — Original pictures for Extended Data Figs. 1a,b and 2q,r. [file 42255_2025_1294_MOESM22_ESM.zip › Original pictures EDF1a/WT-1_Vgat_Gipr_RNAscope_used in paper.jpg]

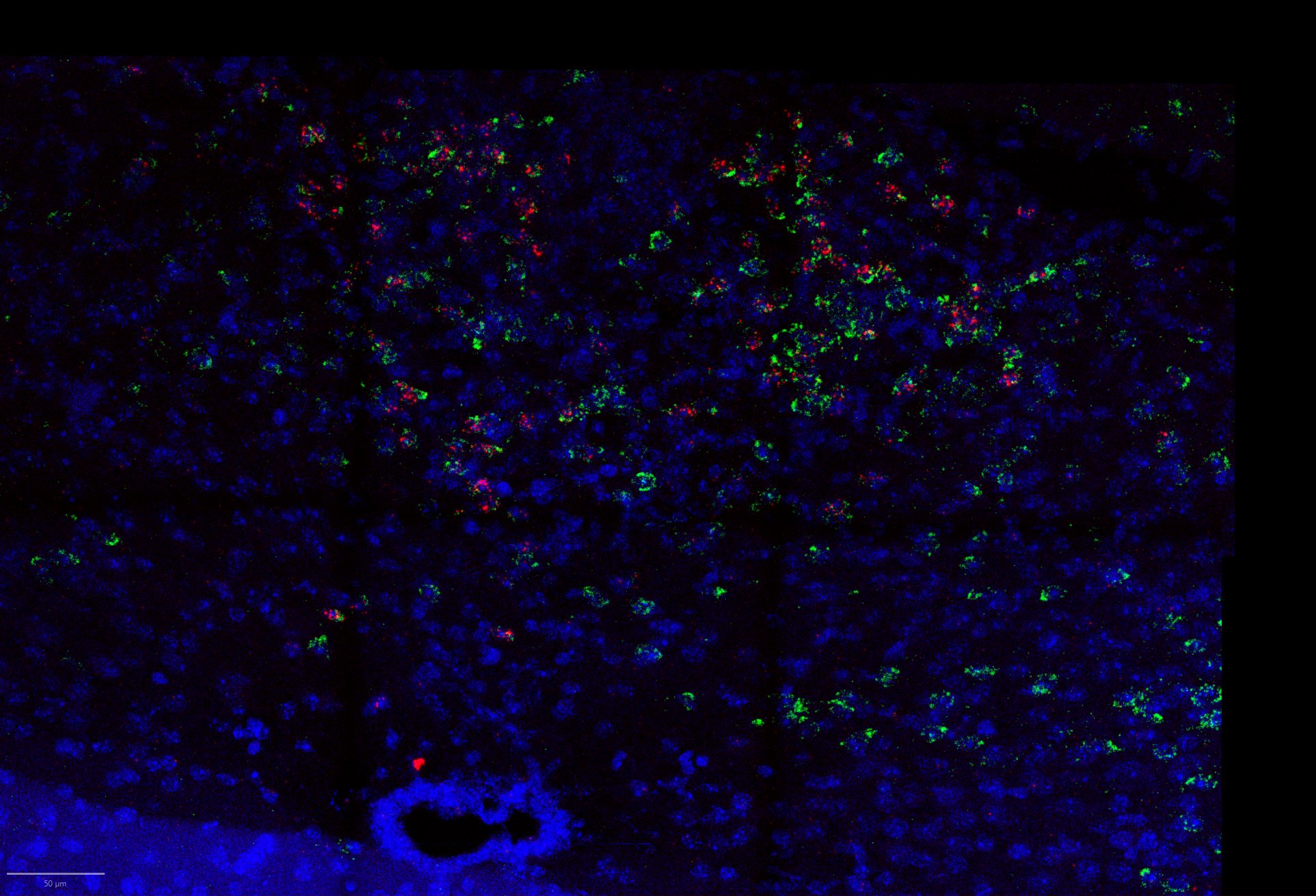

Supplement: Supplementary file 22 — Original pictures for Extended Data Figs. 1a,b and 2q,r. [file 42255_2025_1294_MOESM22_ESM.zip › Original pictures EDF1a/WT-3_Vgat_Gipr_RNAscope.jpg]

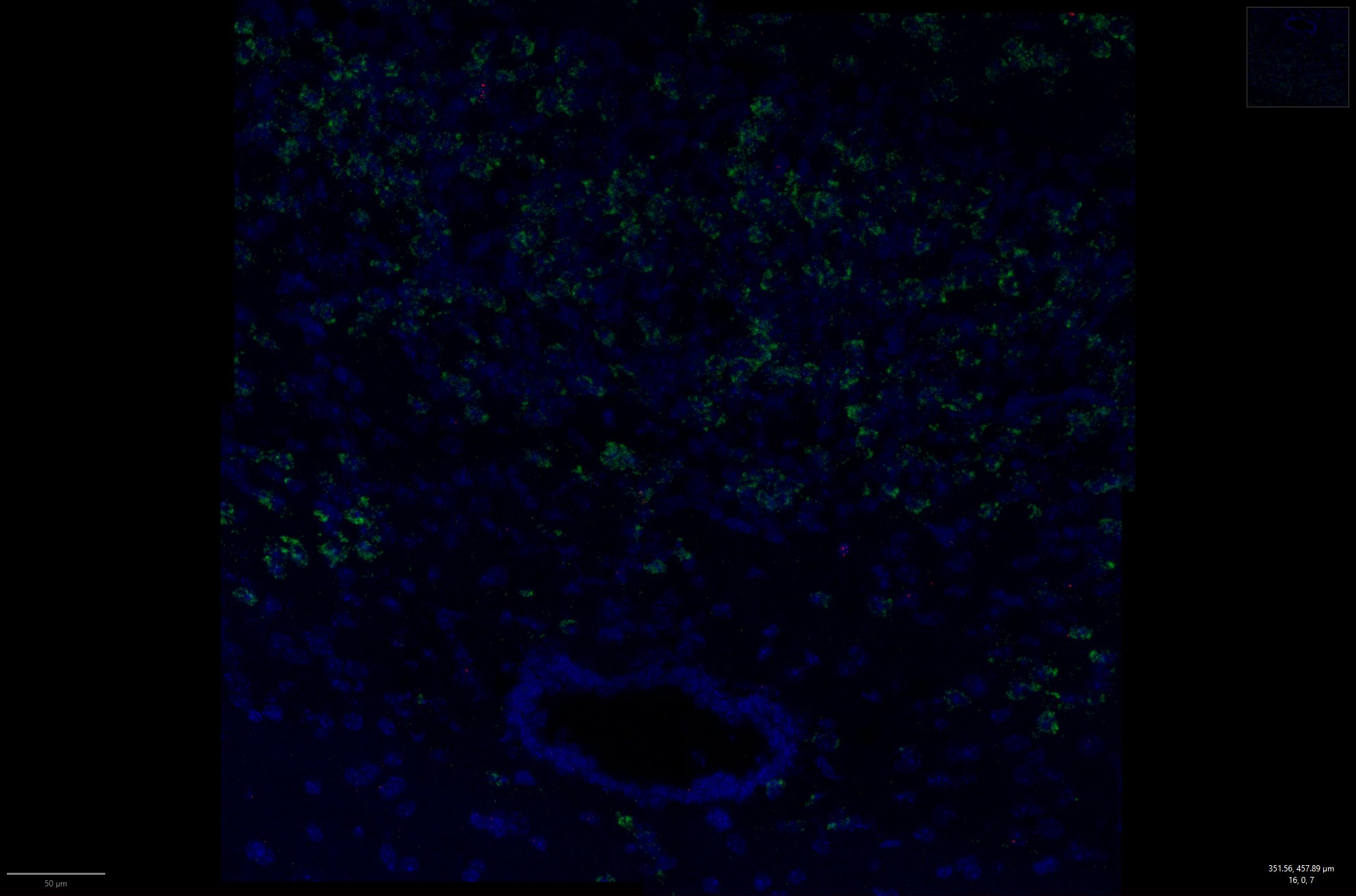

Supplement: Supplementary file 22 — Original pictures for Extended Data Figs. 1a,b and 2q,r. [file 42255_2025_1294_MOESM22_ESM.zip › Original pictures EDF1b/KO-1_Vgat_Gipr_RNAscope.jpg]

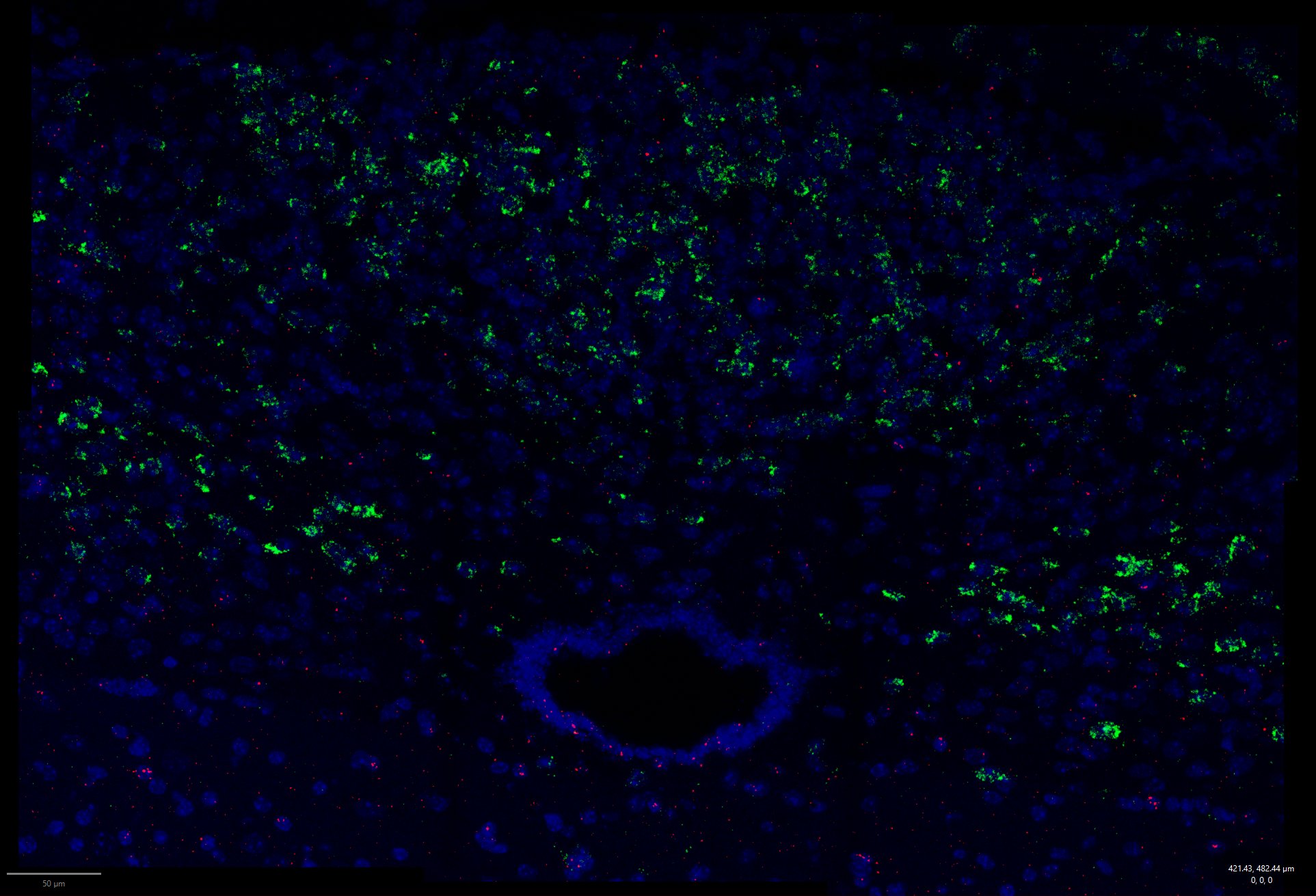

Supplement: Supplementary file 22 — Original pictures for Extended Data Figs. 1a,b and 2q,r. [file 42255_2025_1294_MOESM22_ESM.zip › Original pictures EDF1b/KO-3_Vgat_Gipr_RNAscope_used in paper.jpg]

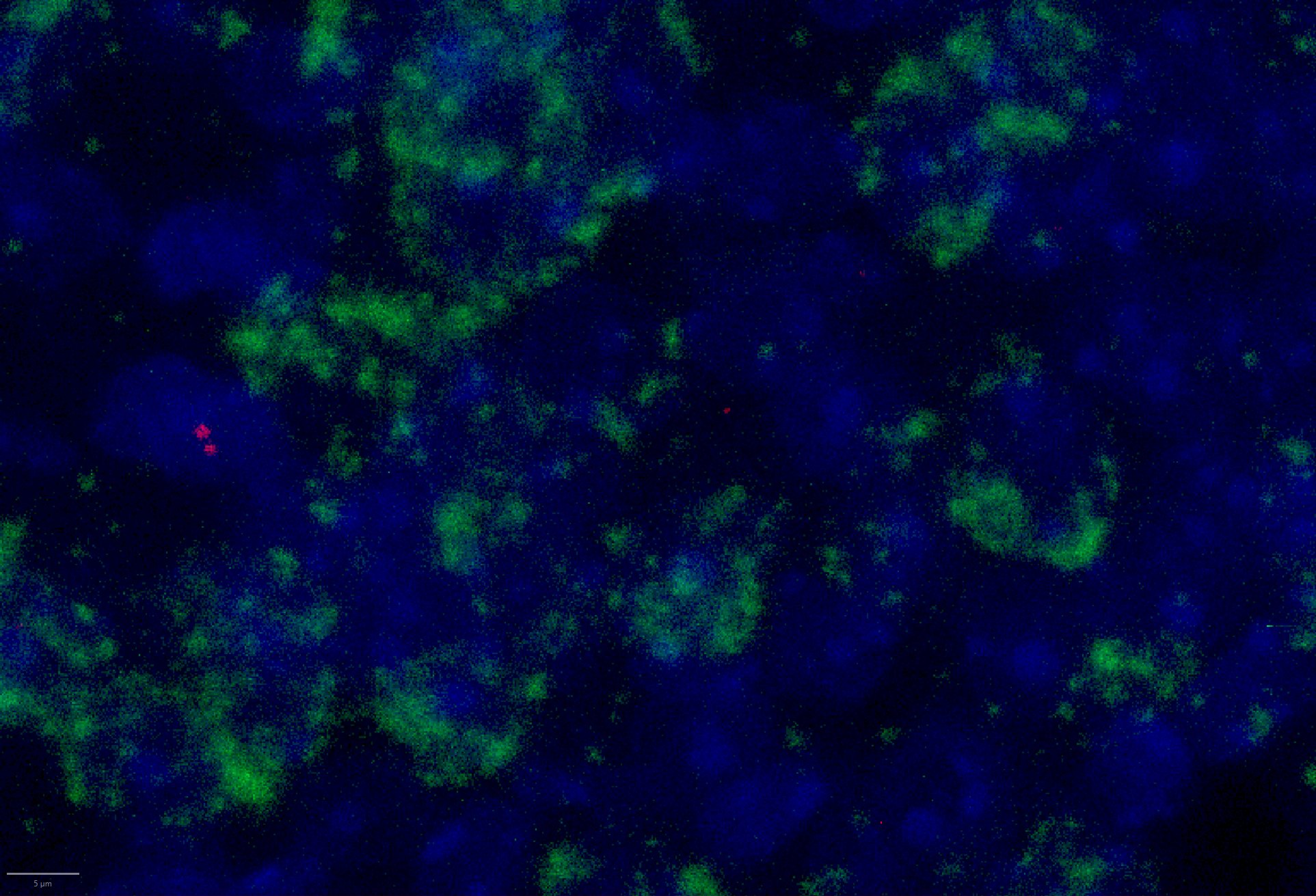

Supplement: Supplementary file 22 — Original pictures for Extended Data Figs. 1a,b and 2q,r. [file 42255_2025_1294_MOESM22_ESM.zip › Original pictures EDF1b/KO_1_Vgat_Gipr_RNAscope_zoom.jpg]

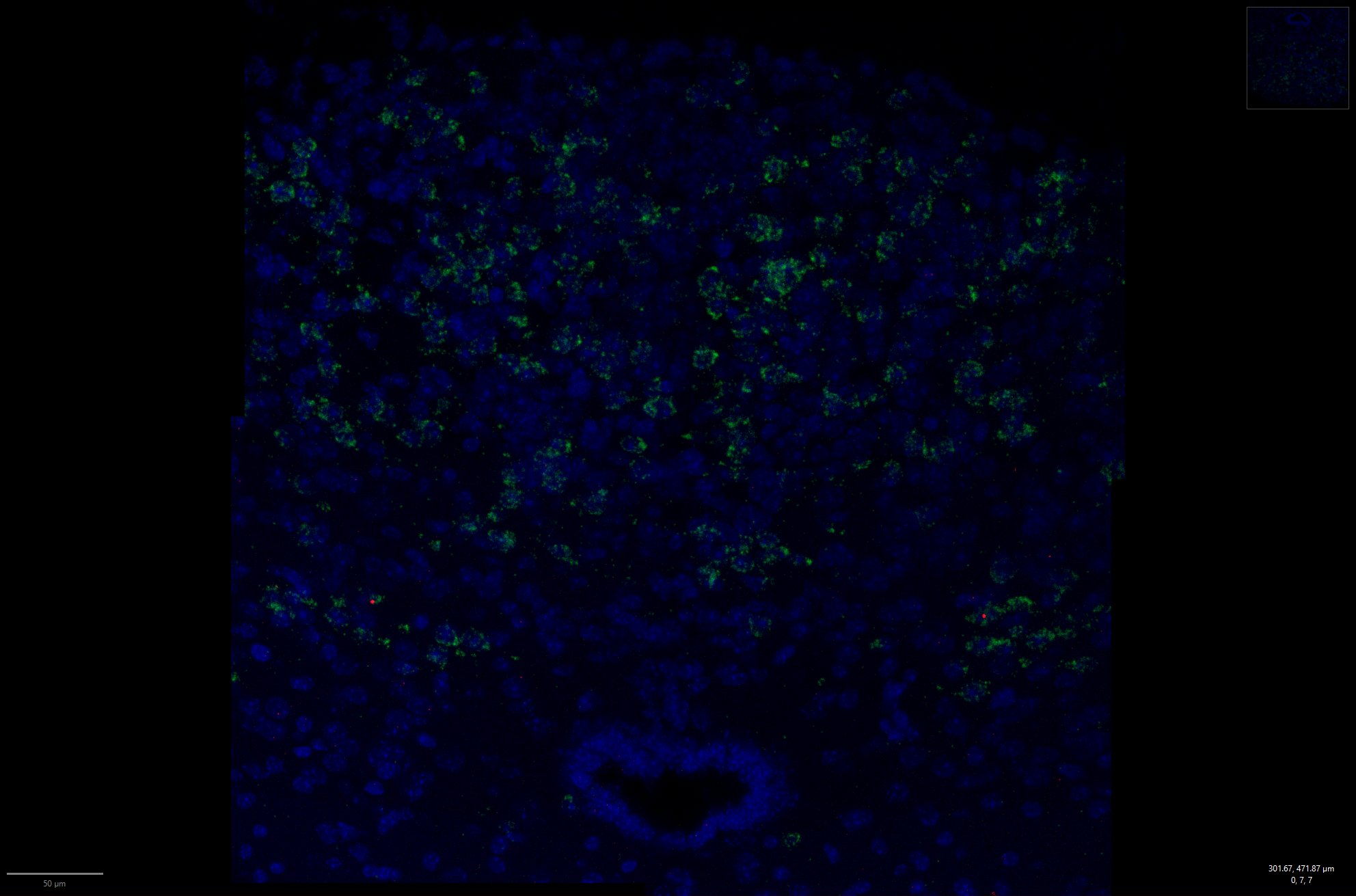

Supplement: Supplementary file 22 — Original pictures for Extended Data Figs. 1a,b and 2q,r. [file 42255_2025_1294_MOESM22_ESM.zip › Original pictures EDF1b/KO-2_Vgat_Gipr_RNAscope.jpg]

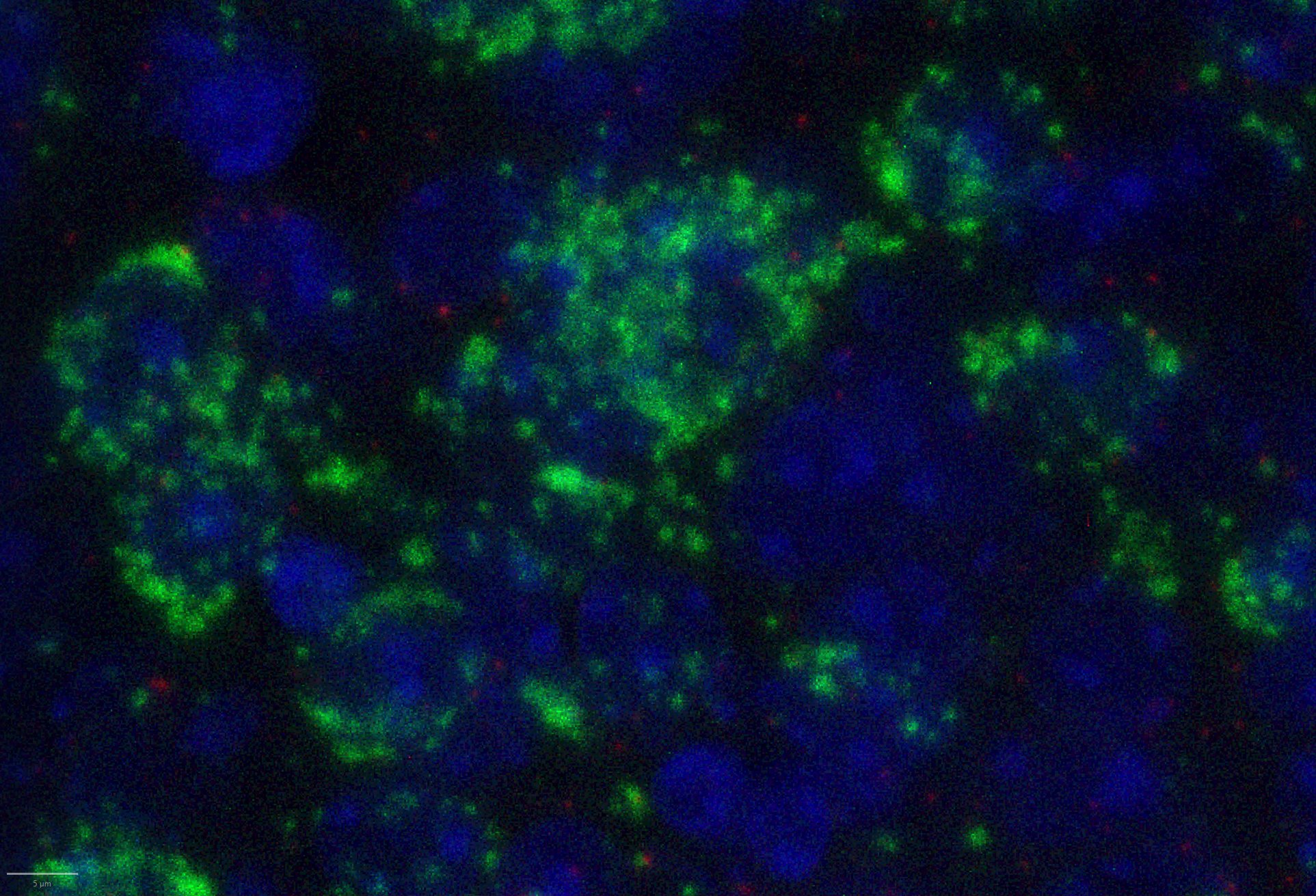

Supplement: Supplementary file 22 — Original pictures for Extended Data Figs. 1a,b and 2q,r. [file 42255_2025_1294_MOESM22_ESM.zip › Original pictures EDF1b/KO-2_Vgat_Gipr_RNAscope_zoom.jpg]

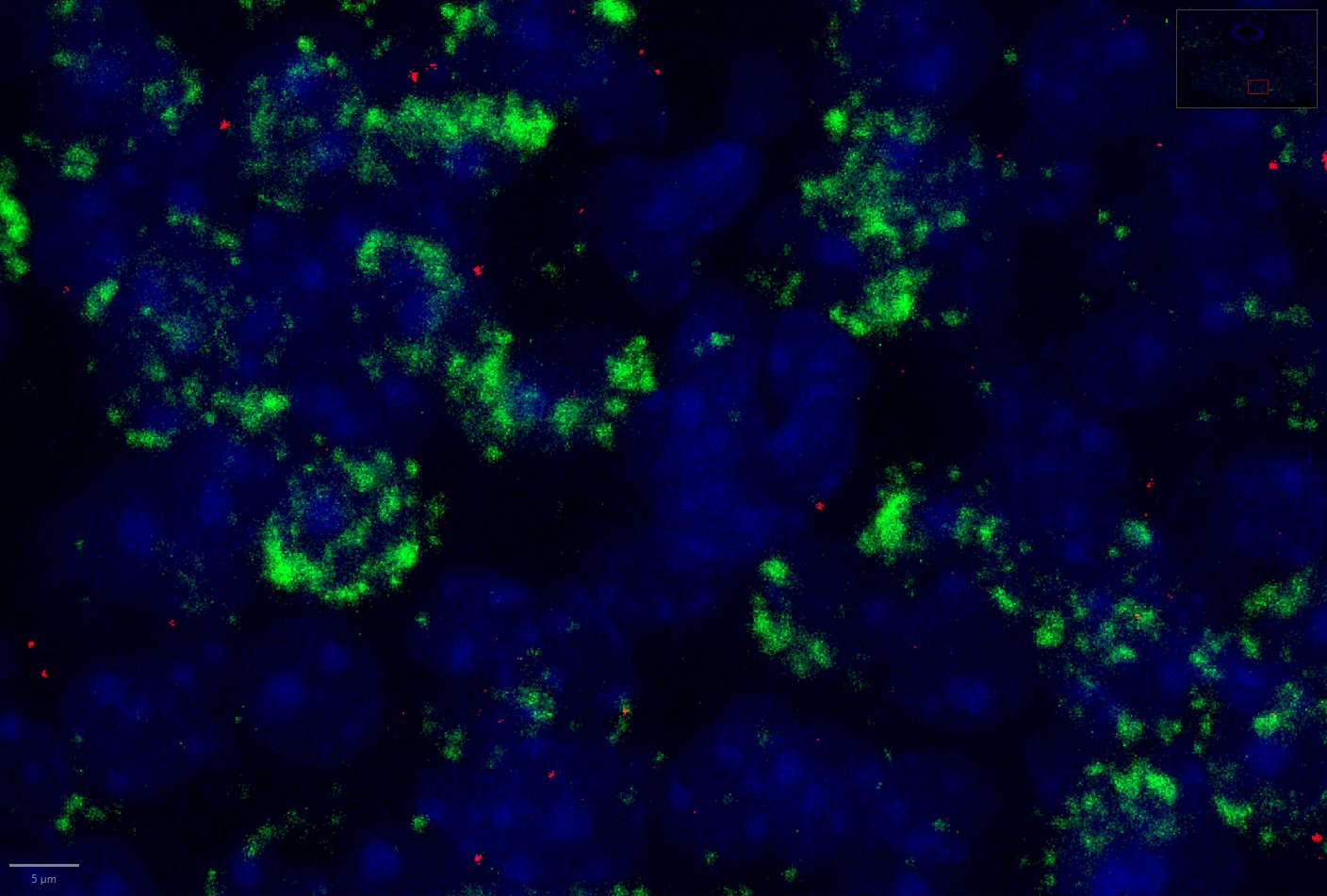

Supplement: Supplementary file 22 — Original pictures for Extended Data Figs. 1a,b and 2q,r. [file 42255_2025_1294_MOESM22_ESM.zip › Original pictures EDF1b/KO-3_Vgat_Gipr_RNAscope_zoom_used in the paper.jpg]

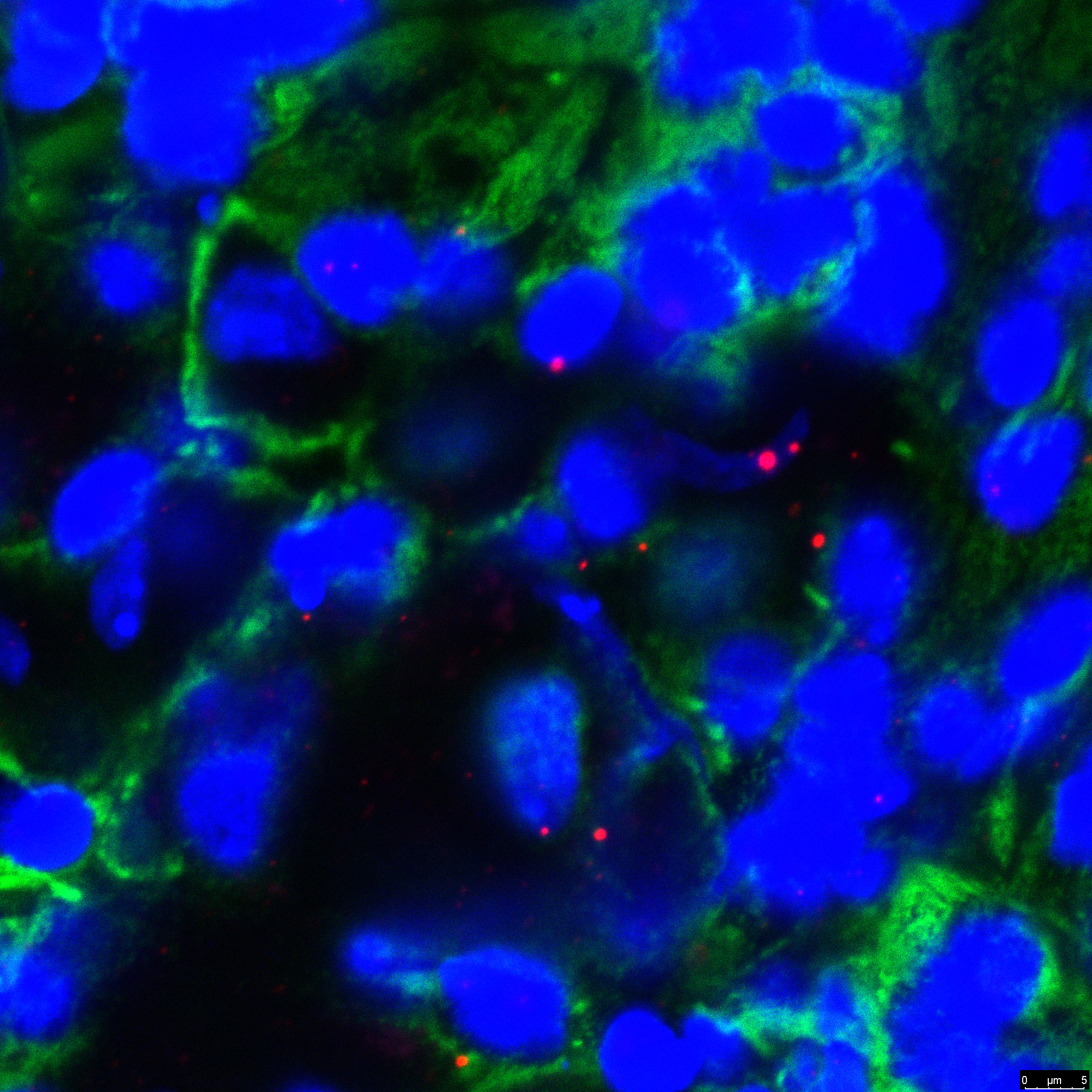

Supplement: Supplementary file 22 — Original pictures for Extended Data Figs. 1a,b and 2q,r. [file 42255_2025_1294_MOESM22_ESM.zip › Original pictures EDF2q/TG_WT2_63x.tif]

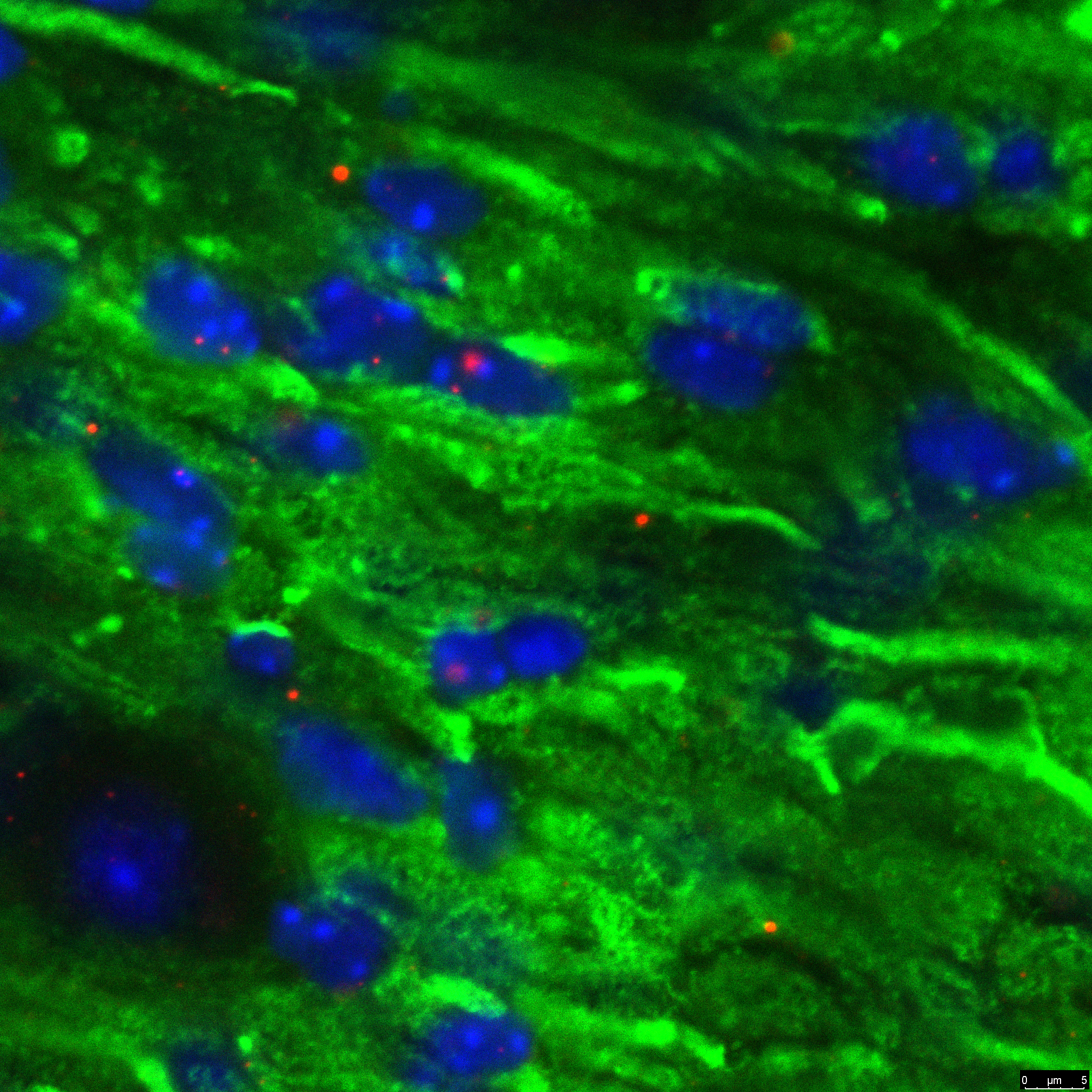

Supplement: Supplementary file 22 — Original pictures for Extended Data Figs. 1a,b and 2q,r. [file 42255_2025_1294_MOESM22_ESM.zip › Original pictures EDF2q/TG_WT1_63x.tif]

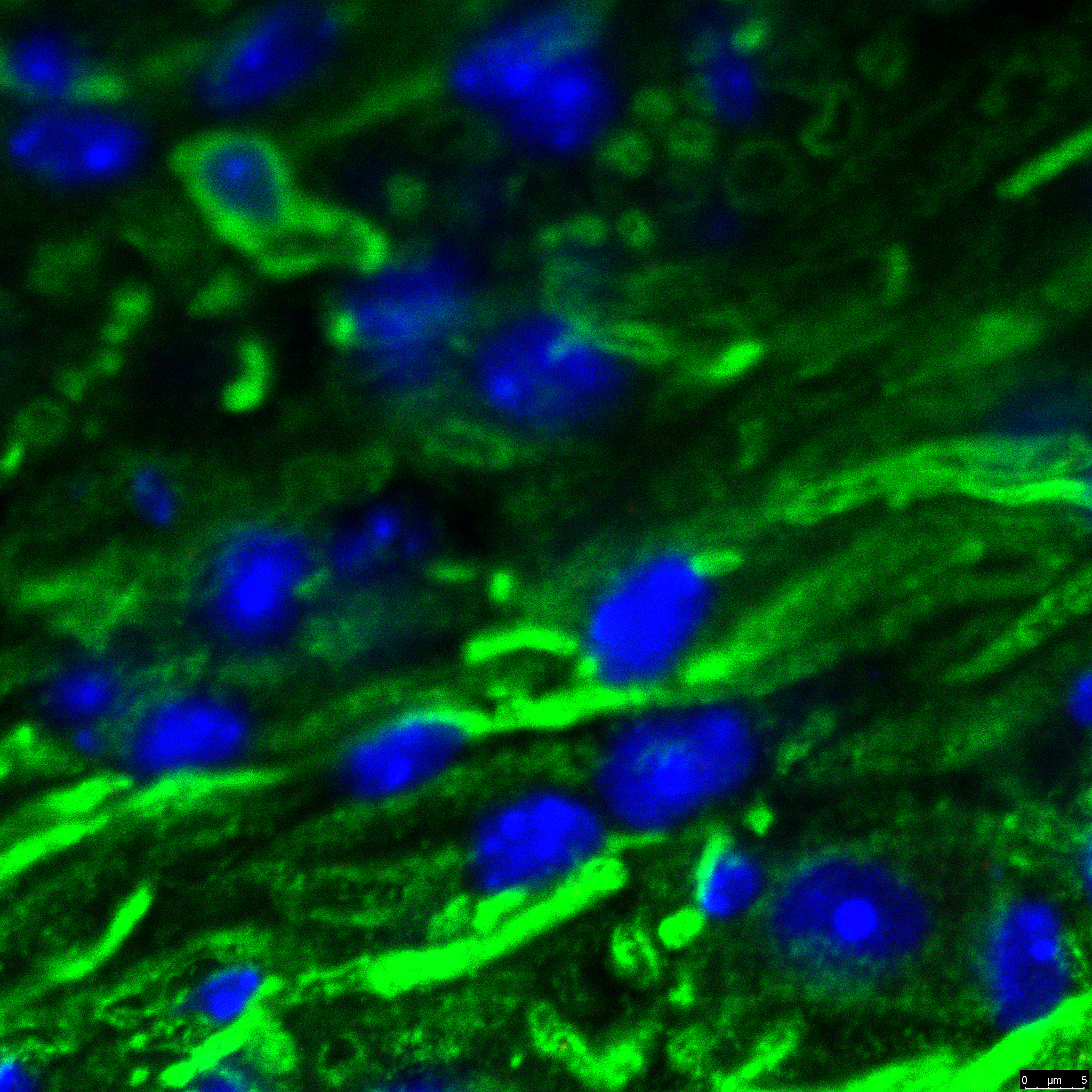

Supplement: Supplementary file 22 — Original pictures for Extended Data Figs. 1a,b and 2q,r. [file 42255_2025_1294_MOESM22_ESM.zip › Original pictures EDF2q/TG_KO2_63x.tif]

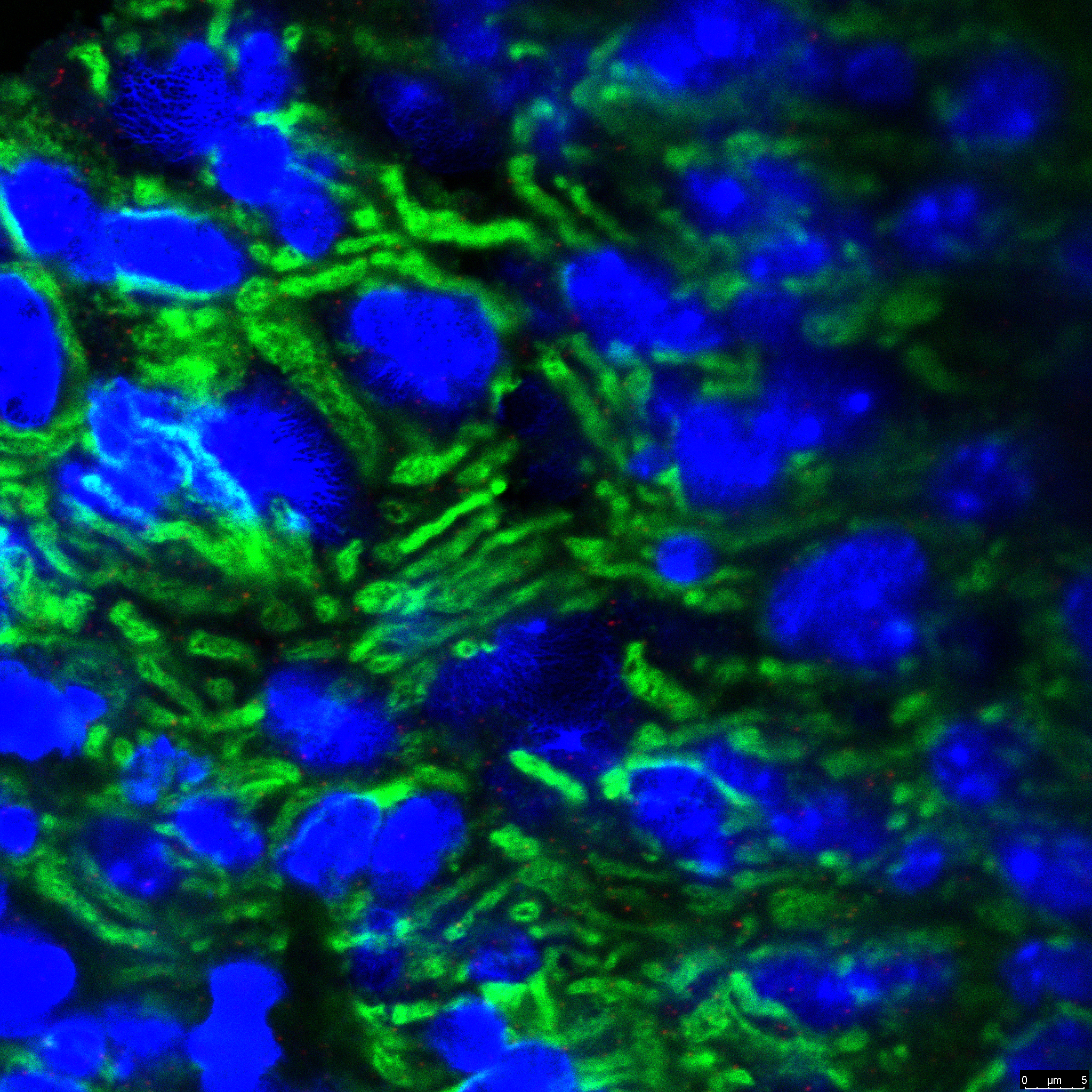

Supplement: Supplementary file 22 — Original pictures for Extended Data Figs. 1a,b and 2q,r. [file 42255_2025_1294_MOESM22_ESM.zip › Original pictures EDF2q/TG_KO3_63x.tif]

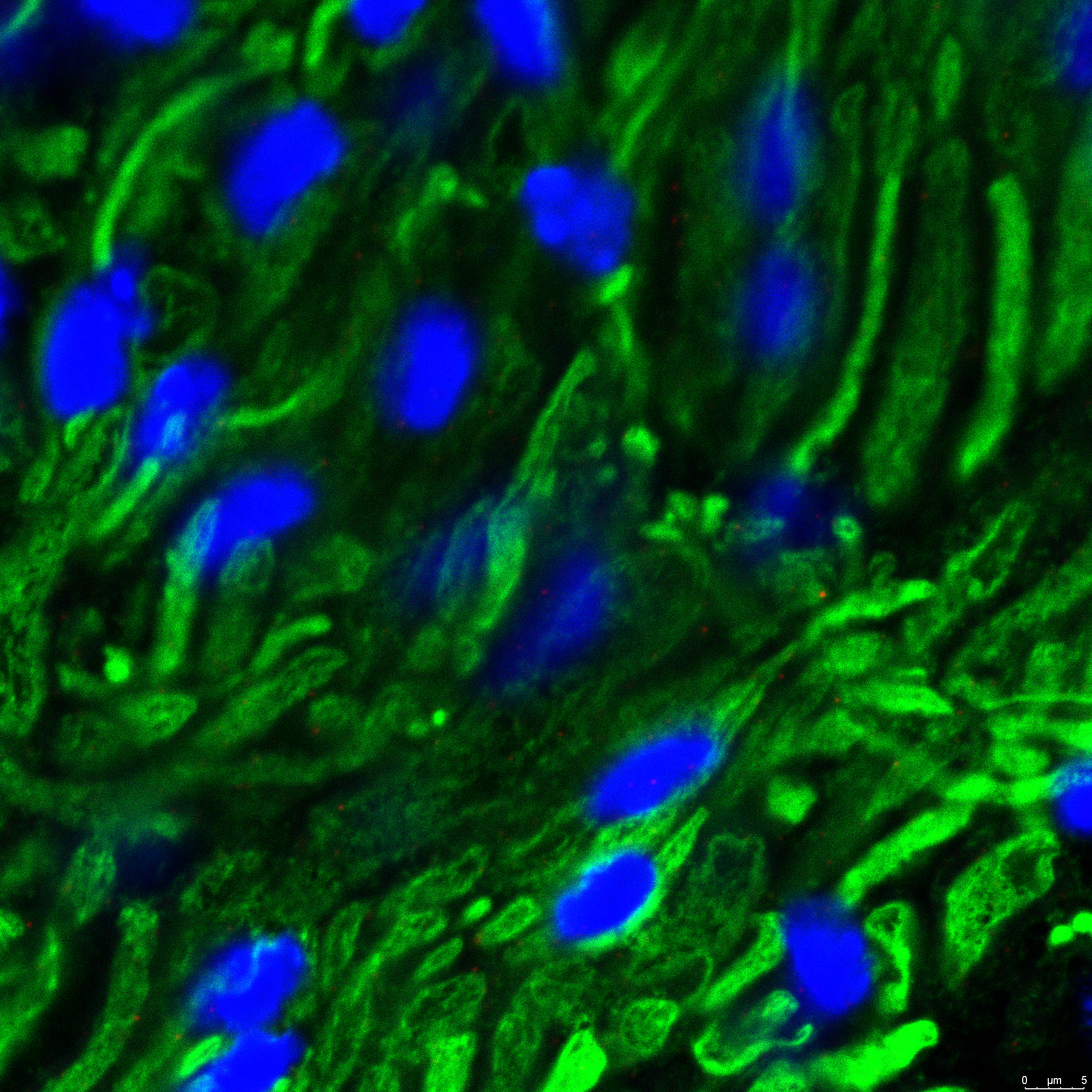

Supplement: Supplementary file 22 — Original pictures for Extended Data Figs. 1a,b and 2q,r. [file 42255_2025_1294_MOESM22_ESM.zip › Original pictures EDF2q/TG_KO1_63x_used in paper.tif]

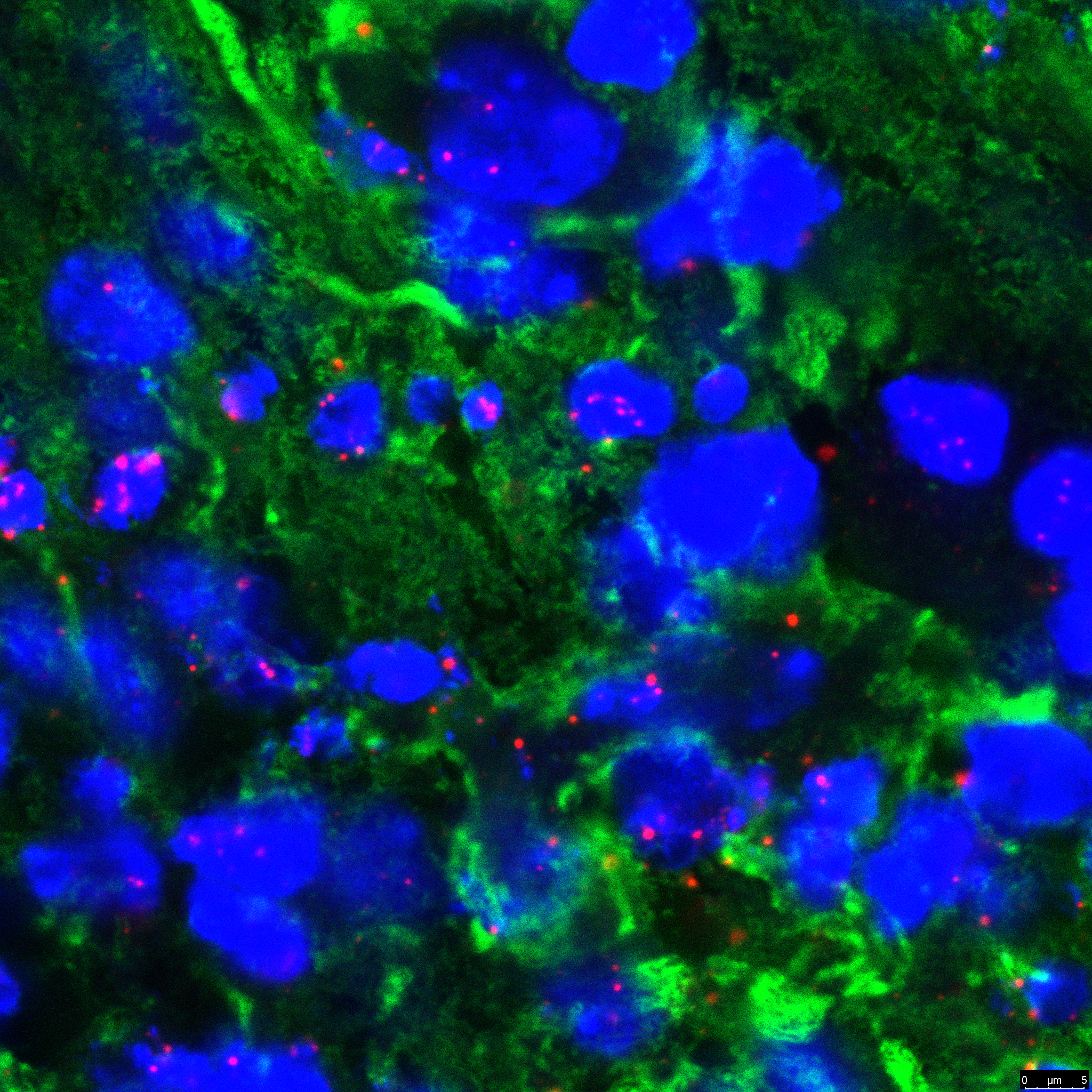

Supplement: Supplementary file 22 — Original pictures for Extended Data Figs. 1a,b and 2q,r. [file 42255_2025_1294_MOESM22_ESM.zip › Original pictures EDF2q/TG_WT3_63x_used in the paper.tif]

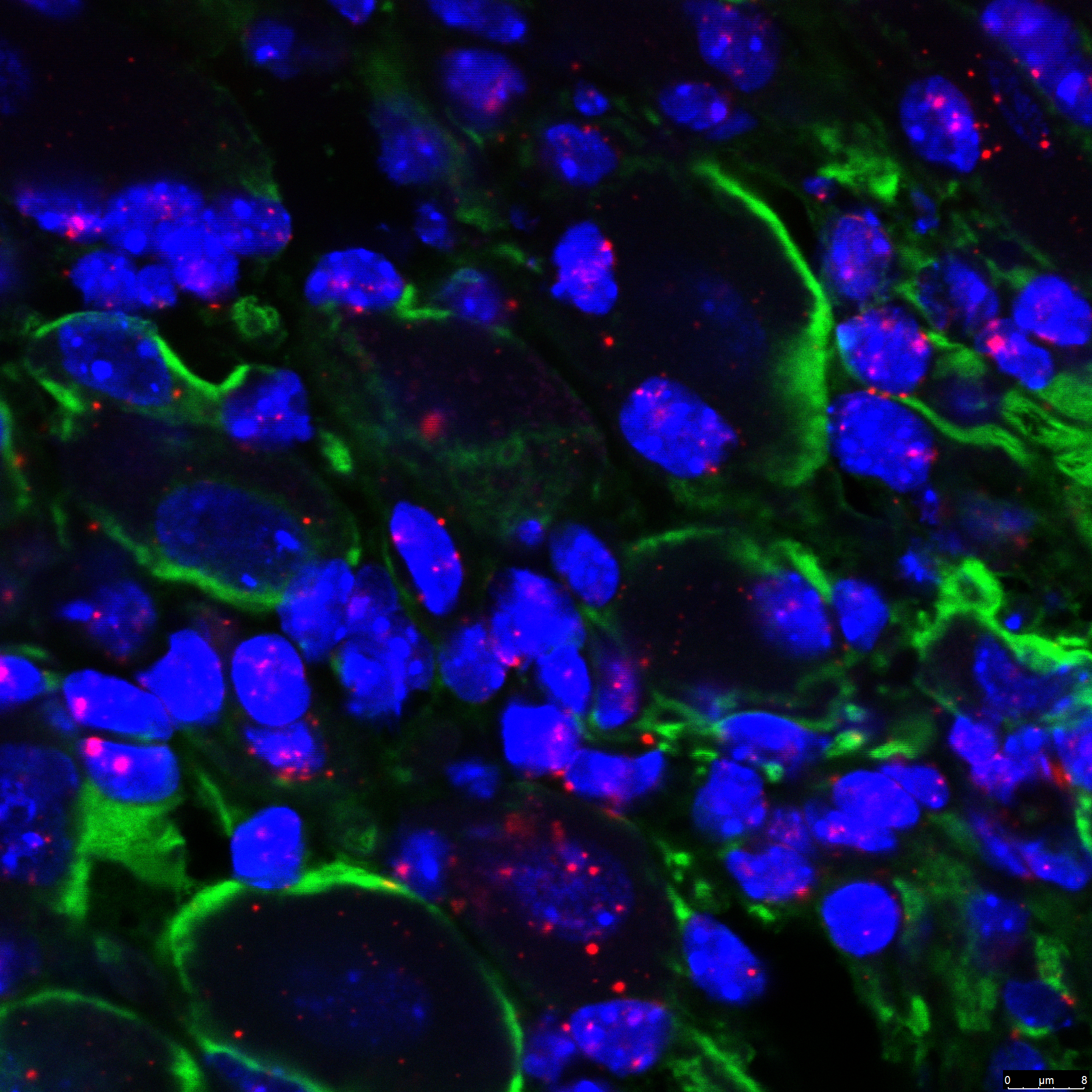

Supplement: Supplementary file 22 — Original pictures for Extended Data Figs. 1a,b and 2q,r. [file 42255_2025_1294_MOESM22_ESM.zip › Original pictures EDF2r/DRG_WT3_63x_used in the paper.tif]

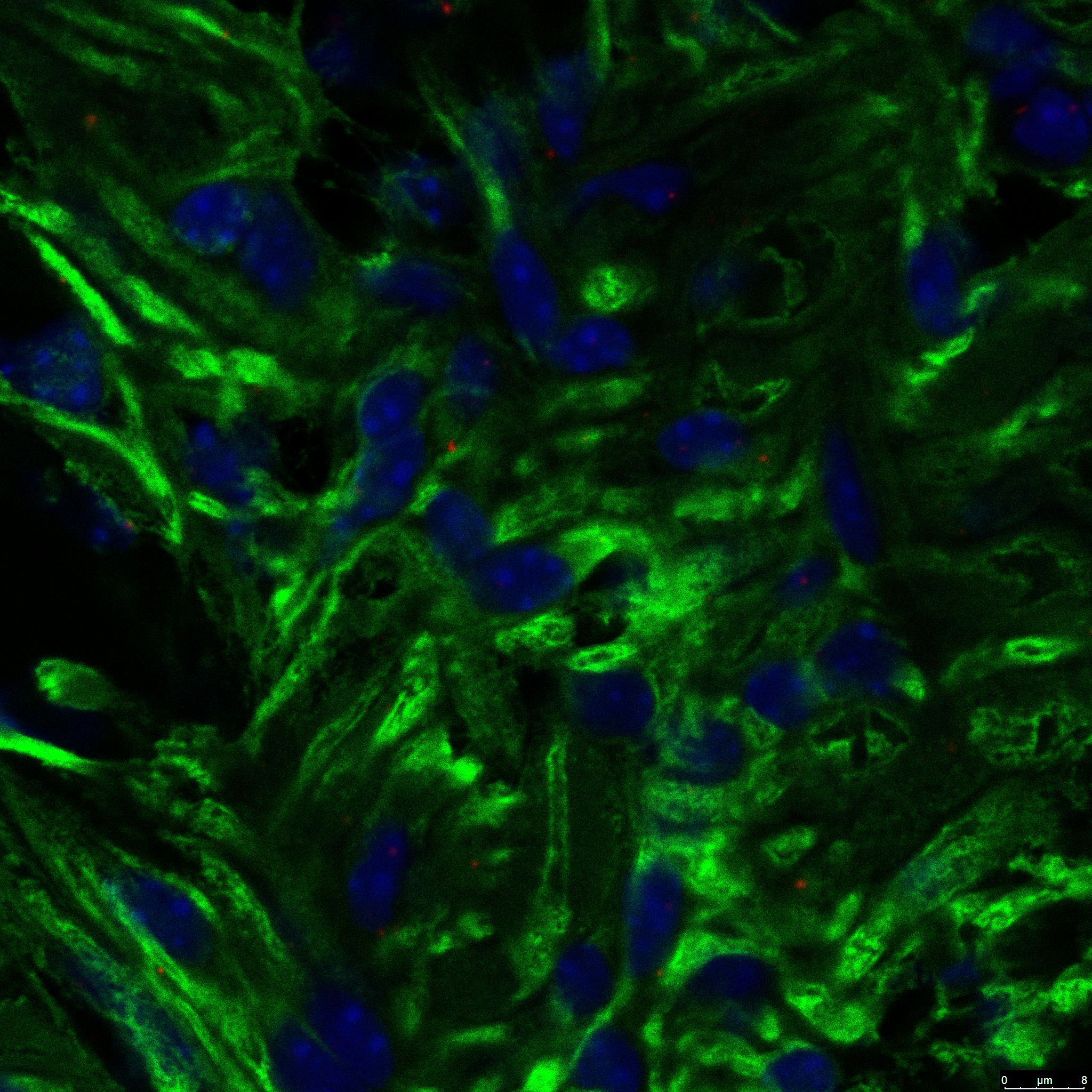

Supplement: Supplementary file 22 — Original pictures for Extended Data Figs. 1a,b and 2q,r. [file 42255_2025_1294_MOESM22_ESM.zip › Original pictures EDF2r/DRG_KO2_63x.jpg]

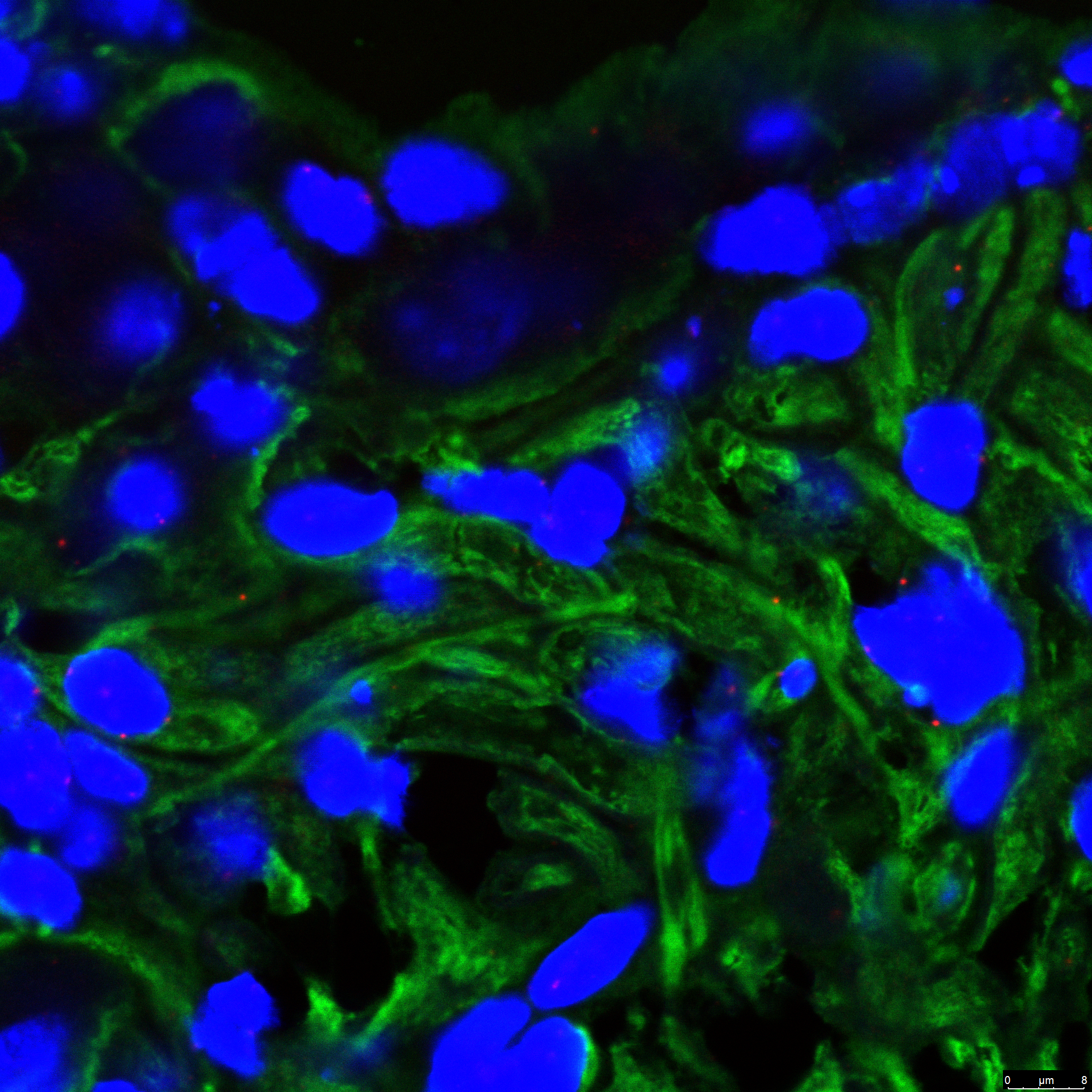

Supplement: Supplementary file 22 — Original pictures for Extended Data Figs. 1a,b and 2q,r. [file 42255_2025_1294_MOESM22_ESM.zip › Original pictures EDF2r/DRG_KO1_63x_used for the paper.tif]

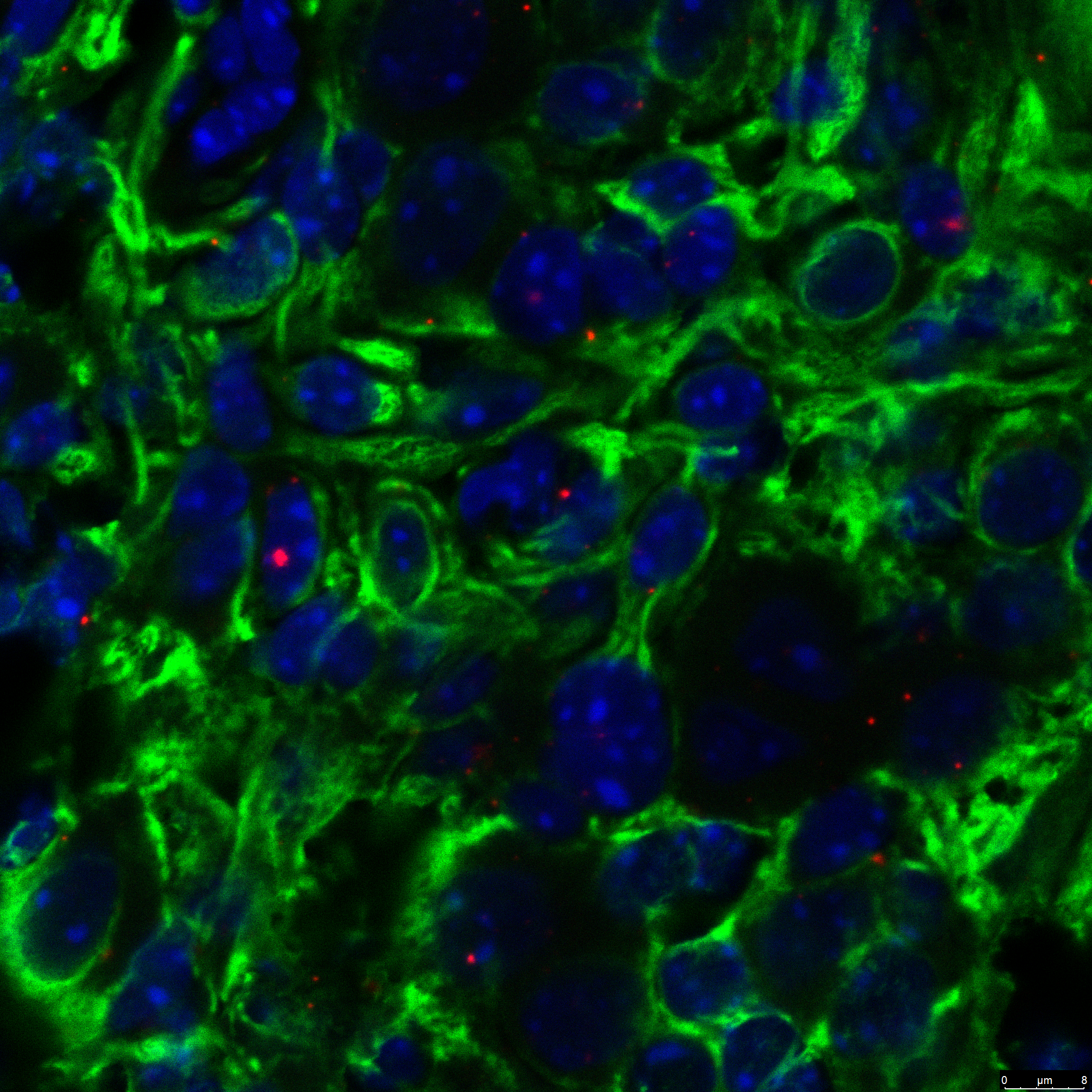

Supplement: Supplementary file 22 — Original pictures for Extended Data Figs. 1a,b and 2q,r. [file 42255_2025_1294_MOESM22_ESM.zip › Original pictures EDF2r/DRG_WT2_63x.tif]

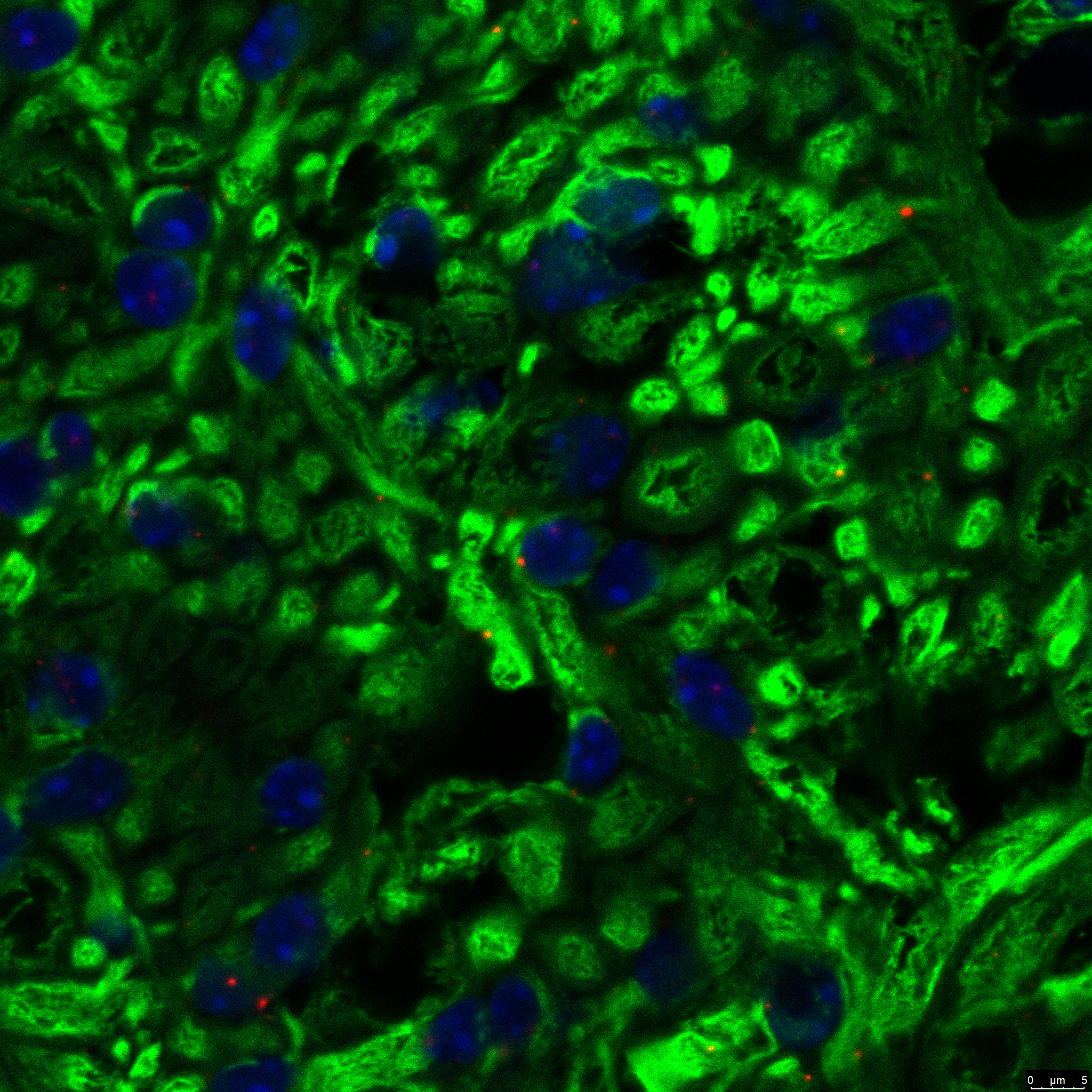

Supplement: Supplementary file 22 — Original pictures for Extended Data Figs. 1a,b and 2q,r. [file 42255_2025_1294_MOESM22_ESM.zip › Original pictures EDF2r/DRG_KO3_63x.tif]

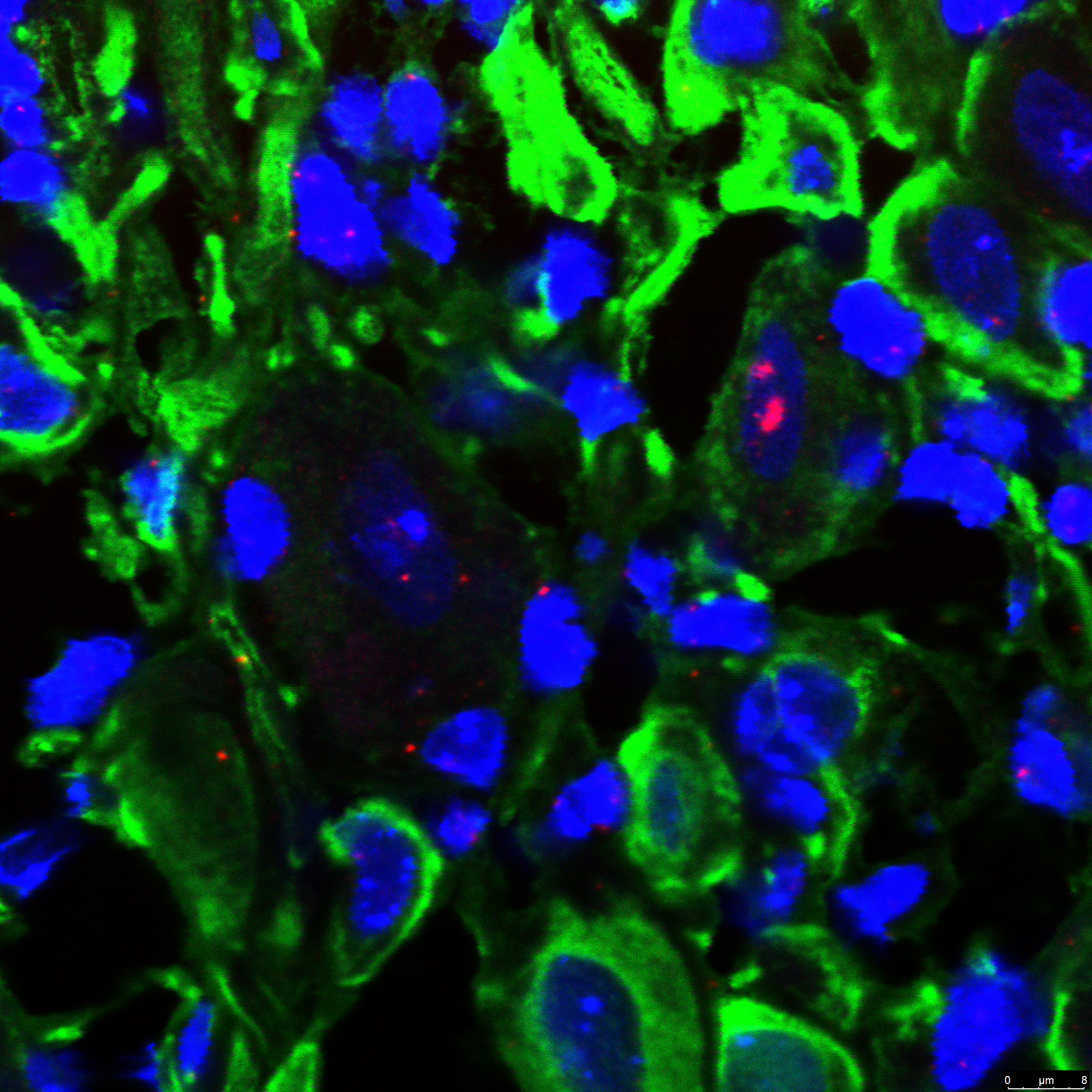

Supplement: Supplementary file 22 — Original pictures for Extended Data Figs. 1a,b and 2q,r. [file 42255_2025_1294_MOESM22_ESM.zip › Original pictures EDF2r/DRG_WT1_63x.tif]
